# Outcomes of Observation vs Stereotactic Ablative Radiation for Oligometastatic Prostate Cancer: The ORIOLE Phase 2 Randomized Clinical Trial

**DOI:** 10.1001/jamaoncol.2020.0147

**Published:** 2020-03-26

**Authors:** Ryan Phillips, William Yue Shi, Matthew Deek, Noura Radwan, Su Jin Lim, Emmanuel S. Antonarakis, Steven P. Rowe, Ashley E. Ross, Michael A. Gorin, Curtiland Deville, Stephen C. Greco, Hailun Wang, Samuel R. Denmeade, Channing J. Paller, Shirl Dipasquale, Theodore L. DeWeese, Daniel Y. Song, Hao Wang, Michael A. Carducci, Kenneth J. Pienta, Martin G. Pomper, Adam P. Dicker, Mario A. Eisenberger, Ash A. Alizadeh, Maximilian Diehn, Phuoc T. Tran

**Affiliations:** 1 Department of Radiation Oncology and Molecular Radiation Sciences, Johns Hopkins University School of Medicine, Baltimore, Maryland; 2 Stanford Cancer Institute, Department of Radiation Oncology, School of Medicine, Stanford University, Stanford, California; 3 Department of Medical Oncology, Johns Hopkins University School of Medicine, Baltimore, Maryland; 4 The Russell H. Morgan Department of Radiology and Radiological Science, Johns Hopkins University School of Medicine, Baltimore, Maryland; 5 The James Buchanan Brady Urological Institute and Department of Urology, Johns Hopkins University School of Medicine, Baltimore, Maryland; 6 Sidney Kimmel Cancer Center, Department of Radiation Oncology, Thomas Jefferson University, Philadelphia, Pennsylvania; 7 Stanford Cancer Institute, Division of Oncology, Department of Medicine, School of Medicine, Stanford University, Stanford, California

## Abstract

**Importance:**

Complete metastatic ablation of oligometastatic prostate cancer may provide an alternative to early initiation of androgen deprivation therapy (ADT).

**Objective:**

To determine if stereotactic ablative radiotherapy (SABR) improves oncologic outcomes in men with oligometastatic prostate cancer.

**Design, Setting, and Participants:**

The Observation vs Stereotactic Ablative Radiation for Oligometastatic Prostate Cancer (ORIOLE) phase 2 randomized study accrued participants from 3 US radiation treatment facilities affiliated with a university hospital from May 2016 to March 2018 with a data cutoff date of May 20, 2019, for analysis. Of 80 men screened, 54 men with recurrent hormone-sensitive prostate cancer and 1 to 3 metastases detectable by conventional imaging who had not received ADT within 6 months of enrollment or 3 or more years total were randomized.

**Interventions:**

Patients were randomized in a 2:1 ratio to receive SABR or observation.

**Main Outcomes and Measures:**

The primary outcome was progression at 6 months by prostate-specific antigen level increase, progression detected by conventional imaging, symptomatic progression, ADT initiation for any reason, or death. Predefined secondary outcomes were toxic effects of SABR, local control at 6 months with SABR, progression-free survival, Brief Pain Inventory (Short Form)–measured quality of life, and concordance between conventional imaging and prostate-specific membrane antigen (PSMA)–targeted positron emission tomography in the identification of metastatic disease.

**Results:**

In the 54 men randomized, the median (range) age was 68 (61-70) years for patients allocated to SABR and 68 (64-76) years for those allocated to observation. Progression at 6 months occurred in 7 of 36 patients (19%) receiving SABR and 11 of 18 patients (61%) undergoing observation (*P* = .005). Treatment with SABR improved median progression-free survival (not reached vs 5.8 months; hazard ratio, 0.30; 95% CI, 0.11-0.81; *P* = .002). Total consolidation of PSMA radiotracer-avid disease decreased the risk of new lesions at 6 months (16% vs 63%; *P* = .006). No toxic effects of grade 3 or greater were observed. T-cell receptor sequencing identified significant increased clonotypic expansion following SABR and correlation between baseline clonality and progression with SABR only (0.082085 vs 0.026051; *P* = .03).

**Conclusions and Relevance:**

Treatment with SABR for oligometastatic prostate cancer improved outcomes and was enhanced by total consolidation of disease identified by PSMA-targeted positron emission tomography. SABR induced a systemic immune response, and baseline immune phenotype and tumor mutation status may predict the benefit from SABR. These results underline the importance of prospective randomized investigation of the oligometastatic state with integrated imaging and biological correlates.

**Trial Registration:**

ClinicalTrials.gov Identifier: NCT02680587

## Key Points

**Question:**

How effectively does stereotactic ablative radiotherapy prevent progression of disease compared with observation in men with recurrent hormone-sensitive prostate cancer with 1 to 3 metastases?

**Findings:**

In this phase 2 randomized clinical trial of 54 men, progression of disease at 6 months occurred in 7 of 36 participants (19%) treated with stereotactic ablative radiotherapy and in 11 of 18 participants (61%) undergoing observation, a statistically significant difference.

**Meaning:**

Stereotactic ablative radiotherapy is a promising treatment approach for men with recurrent hormone-sensitive oligometastatic prostate cancer who wish to delay initiation of androgen deprivation therapy.

## Teaser

This randomized clinical trial assesses whether treatment with stereotactic ablative radiotherapy vs observation improves oncologic outcomes in men with recurrent hormone-sensitive oligometastatic prostate cancer.

## Introduction

In the US, prostate cancer is the third most common cancer overall and the most common in men, accounting for approximately 30 000 deaths per year.^1^ Metastatic prostate cancer remains incurable despite advances in systemic management for hormone-sensitive^2^ and castration-resistant disease.^3^

The oligometastatic state described by Hellman and Weichselbaum^4^ may benefit from localized therapies, and mounting prospective evidence supports the inclusion of radiotherapy in the metastatic paradigm. Two trials^5,6^ have shown that stereotactic ablative radiotherapy (SABR) significantly improves progression-free survival (PFS) and overall survival in patients with oligometastatic non–small cell lung cancer when added to maintenance systemic therapy, and the Stereotactic Ablative Radiotherapy for the Comprehensive Treatment of Oligometastases (SABR-COMET) trial^7^ reported an overall survival benefit with SABR in patients with oligometastases when used in addition to standard-of-care systemic therapy across histologies.

In the treatment of prostate cancer, radiotherapy has demonstrated clinical benefits in both de novo and metachronous low-volume metastatic disease. Parker et al^8^ showed that the addition of prostate radiotherapy to standard systemic treatment improves overall survival for men with de novo metastatic prostate cancer with low metastatic burden. In the Surveillance or Metastasis-Directed Therapy for Oligometastatic Prostate Cancer Recurrence (STOMP) trial, to our knowledge the first phase 2 randomized clinical trial of SABR vs observation in oligometastatic prostate cancer (OMPC), Ost et al^9^ found significantly longer time to initiation of androgen deprivation therapy (ADT) in men treated with SABR. Although the approach is controversial, many men are interested in avoiding the unpleasant adverse effects and potential health risks of ADT for as long as is reasonable. With early clinical data suggesting the existence of an oligometastatic state and the importance of local therapies in management, strategies are now needed to define who may benefit most from metastasis-directed therapy (MDT).^10^

This question is multifaceted, but 2 key components are (1) determining which patients truly have oligometastatic disease and (2) ascertaining who is most likely to experience a meaningful response to local consolidation. To answer the former, advanced imaging and circulating biomarkers, such as microRNA^11,12,13,14^ and circulating tumor DNA (ctDNA),^15,16,17^ may improve our ability to characterize disease burden and behavior. To address the latter requires a more complete understanding of response to radiotherapy^18^ and the complementary role of the immune system.^19,20^

This study reports the findings of a phase 2 randomized clinical trial of observation vs SABR in men with hormone-sensitive OMPC, to our knowledge the first in the western hemisphere. The study also shows the value of the prostate-specific membrane antigen (PSMA)–targeted positron emission tomography (PET) radiotracer ^18^F-DCFPyL and liquid biopsy correlatives in defining patients with oligometastasis who would benefit the most from MDT.

## Methods

### Study Design and Participants

The Observation vs Stereotactic Ablative Radiation for Oligometastatic Prostate Cancer (ORIOLE) 2-arm, phase 2 randomized clinical trial was approved by the Johns Hopkins University Institutional Review Board and performed across 3 affiliated centers in the US. Patients eligible for enrollment had 1 to 3 asymptomatic metastases that had arisen within the prior 6 months and were no larger than 5.0 cm in the largest axis or 250 cm^2^. The number of metastases was assessed by computed tomography (CT), magnetic resonance imaging, and/or radionuclide bone scan. All patients had histologic confirmation of prostate cancer and prior definitive treatment of the primary tumor with surgery or radiotherapy. Salvage radiotherapy to the prostate bed or pelvis was allowed. Patients were allowed to have received ADT or other systemic therapy during initial management or salvage treatment but not within 6 months of enrollment. The trial protocol is available in Supplement 1. Additional inclusion criteria and full exclusion criteria are available in eMethods in Supplement 2. All study participants provided written informed consent approved by the institutional review board. This study followed the Consolidated Standards of Reporting Trials (CONSORT) reporting guideline.

### Randomization and Blinding

Participants were randomized to the SABR or observation arm in a 2:1 ratio using an interactive web response system. Minimization^21^ was applied to balance assignment based on stratification by initial treatment (surgery or radiotherapy), history of prior ADT or lack thereof, and prostate-specific antigen (PSA) doubling time (<6 months vs 6-14.9 months) (eMethods in Supplement 2). Neither the treating physician, the participants, nor the personnel responsible for data analysis were blinded to assignment. The trial radiologist assessing response by CT size criteria and by ^18^F-DCFPyL uptake was blinded to the participant treatment arm and to the treatment fields used (eMethods in Supplement 2).

### Procedures

For assessment of eligibility, patients provided a comprehensive medical history, underwent a physical examination, and had blood drawn for analysis of complete blood count, serum chemistry measurements, and PSA level. Radiographic studies were performed as necessary to complete staging. After randomization, participants underwent routine laboratory testing on days 1, 90, and 180 as well as collection of blood for correlative studies and PSMA-targeted PET-CT (performed at baseline and day 180 for patients randomized to SABR) (eMethods in Supplement 2).

Participants underwent CT-based simulation with customized immobilization. Magnetic resonance imaging–based simulation and 4-dimensional CT were performed at the discretion of the treating physician. Gross tumor volume delineation was performed by the treating radiation oncologist with the addition of a variable expansion of up to 5 mm to generate the planning target volume. Adjacent organs at risk were delineated by the treating radiation oncologist. A stereotactic body radiotherapy plan was then generated with dose and fractionation determined based on the size and location of each lesion, with prescription doses ranging from 19.5 to 48.0 Gy in 3 to 5 fractions (eTable 1 in Supplement 2) and normal tissue constraints per American Association of Physicists in Medicine Task Group 101 recommendations.^22^ Treatment was initiated within 3 weeks of simulation. Image guidance with daily cone beam CT prior to treatment was used for all participants.

### Outcomes

The primary outcome was the proportion of men in each arm with disease progression at 6 months. Progression was a composite end point that included any of the following: a PSA rise of at least 2 ng/dL (to convert to micrograms per liter, multiply by 0.01) and 25% above nadir; concern for radiologic progression by CT, magnetic resonance imaging, or bone scan as determined by the reading radiologist; symptomatic progression of disease; initiation of ADT for any reason; or death. Withdrawal from the study after randomization was considered progression.

Predefined secondary outcomes included the adverse effects of SABR as defined by the Common Terminology Criteria for Adverse Events (version 4.0), local control at 6 months for lesions treated with SABR, PFS, quality of life as measured by the Brief Pain Inventory (Short Form), the concordance between conventional imaging and PSMA-targeted PET in the identification of metastatic disease, and sequencing of T-cell receptor repertoires from peripheral blood mononuclear cells using ImmunoSEQ (Adaptive Biotechnologies).

For radiologic evaluation of lesions that did not meet formal Response Evaluation Criteria in Solid Tumors (RECIST) version 1.1 criteria, progression by imaging was assessed based on the blinded professional assessment of the primary radiologist reading the images combined with application of the RECIST version 1.1 size criteria to all measurable lesions, including those not meeting formal size criteria. To minimize the risk of underestimating local progression, any evidence of progression by size was counted as a progression.

### Statistical Analysis

Briefly, comparisons of progression at 6 months and presence of new metastases at 6 months were performed using the 2-sided Fisher exact test. PFS curves were generated using the Kaplan-Meier method, and *P* values were calculated using the log-rank test. Brief Pain Inventory responses were compared between and within arms across time using the Holm-Sidak method for multiple *t* tests. Differential clonotype abundance and ctDNA allele fraction comparisons between arms were performed using 2-tailed Mann-Whitney tests. Statistical significance was defined as *P* < .05. Statistical analysis was performed using Prism version 8 (GraphPad Software) and Rstudio version 1.2.5 (Rstudio Inc). All analysis was performed on an intention-to-treat basis, and further details are available in eMethods in Supplement 2.

## Results

**Figure 1.**
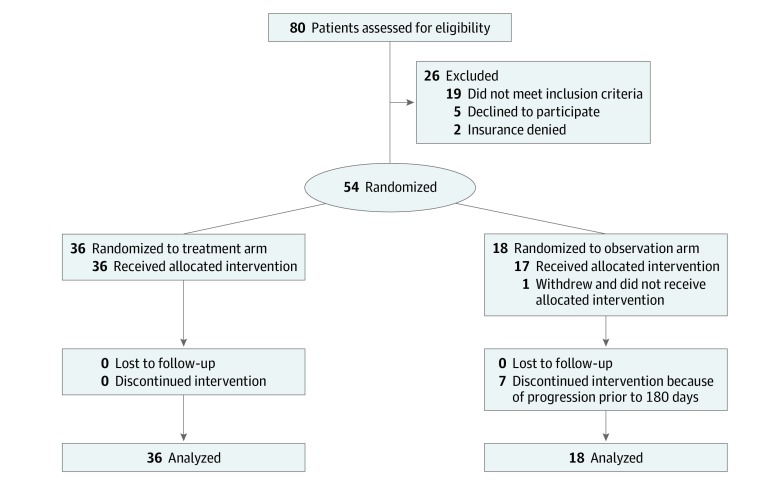
CONSORT Diagram

Between May 25, 2016, and March 5, 2018, a total of 80 men were screened and 54 were randomized in a 2:1 ratio to SABR or observation (Figure 1). Of the 54 men randomized, the median (range) age was 68 (61-70) years for patients allocated to SABR and 68 (64-76) years for those allocated to observation. The follow-up period for each participant extended from the date of randomization to the most recent clinical contact as of May 20, 2019 (median [range] follow-up of 18.8 [5.8-35.0] months), and the trial was completed 6 months after randomization of the final participant. The Table and eTable 2 in Supplement 2 summarize participant and lesion characteristics, respectively. Gleason grade was higher in the observation arm than in the SABR arm with mean values of 8 and 7, respectively. The arms were otherwise well balanced.

**Table.** Baseline Patient Characteristics

Characteristic	No. (%)	
	SABR (n = 36)	Observation (n = 18)
Age, median (range), y	68 (61-70)	68 (64-76)
Initial T stage		
cT1c	3 (8)	1 (6)
cT2a	2 (6)	0
cT2b	0	1 (6)
cT3a	1 (3)	1 (6)
pT2	12 (33)	6 (33)
pT3a	10 (28)	8 (44)
pT3b	8 (22)	1 (6)
Initial N stage		
N0	31 (86)	16 (89)
N1	2 (6)	1 (6)
NX	3 (8)	1 (6)
Margin status		
R0	20 (56)	10 (56)
R1	10 (28)	5 (28)
Gleason grade		
3 + 3 = 6	3 (8)	0
3 + 4 = 7	8 (22)	4 (22)
4 + 3 = 7	14 (39)	4 (22)
4 + 4 = 8	4 (11)	1 (6)
4 + 5 = 9	4 (11)	8 (44)
5 + 4 = 9	3 (8)	0
5 + 5 = 10	0	1 (6)
Initial management		
Surgery	30 (83)	15 (83)
Radiotherapy	6 (17)	3 (17)
Time to first recurrence, median (range), mo	22 (9-42)	22 (9-51)
Had received prior ADT	15 (42)	5 (28)
Baseline, median (range)		
PSA, ng/dL	6 (2-13)	7 (3-17)
PSADT, mo	8 (4-11)	6 (4-11)

Abbreviations: ADT, androgen deprivation therapy; PSA, prostate-specific antigen; PSADT, prostate-specific antigen doubling time; SABR, stereotactic ablative radiotherapy.

SI conversion factor: To convert PSA to μg/L, multiply by 0.01.

**Figure 2.**
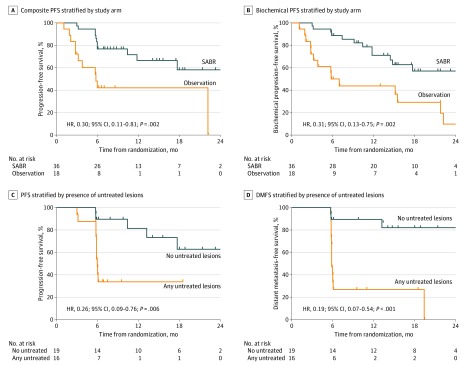
Clinical Outcomes of Stereotactic Ablative Radiotherapy (SABR) Compared With Observation and Benefit of Total Consolidation of Prostate-Specific Membrane Antigen Radiotracer-Avid Lesions A, Composite progression-free survival (PFS) stratified by study arm. B, Biochemical PFS stratified by study arm. C, Composite PFS and (D) distant metastasis–free survival (DMFS) for patients treated by SABR stratified by presence of untreated lesions detected by prostate-specific membrane antigen–positron emission tomography.

The proportion of men with disease progression by composite end point at 6 months was 7 of 36 patients (19%; 95% CI, 9.6-35.4) treated with SABR and 11 of 18 patients (61%; 95% CI, 38.5-79.6) in the observation arm (*P* = .005). The proportion of participants with disease progression by PSA level at 6 months was 4 of 36 patients (11%; 95% CI, 3.9-26.1) treated with SABR and 9 of 18 patients (50%; 95% CI, 29.1-70.9) in the observation arm (*P* = .005). The median PFS for participants treated with SABR was not reached compared with 5.8 months for those undergoing observation (hazard ratio [HR], 0.30; 95% CI, 0.11-0.81; *P* = .002) (Figure 2A). Median biochemical PFS was not reached for patients treated with SABR and was 6.4 months for those undergoing observation (HR, 0.31; 95% CI, 0.13-0.75; *P* = .002) (Figure 2B). Local control was excellent as expected (98.9%) at 6 months (eResults, eFigure 1, and eTable 1 in Supplement 2).

Because of blinding of the investigative team to the PSMA-targeted PET data during treatment planning, 16 of 36 participants treated with SABR had baseline PET-avid lesions that were not included in the treatment fields. The proportion of men with no untreated lesions with progression at 6 months was 1 of 19 (5%; 95% CI, 0-26.8) compared with 6 of 16 (38%; 95% CI, 18.5-61.5) for those with any untreated lesions (*P* = .03). The median PFS was unreached among participants with no untreated lesions vs 11.8 months among participants with any untreated lesions (HR, 0.26; 95% CI, 0.09-0.76; *P* = .006) (Figure 2C). The proportion of men who developed new metastatic lesions at 180 days was 3 of 19 (15.8%; 95% CI, 4.9-38.6) with no untreated lesions and 10 of 16 (62.5%; 95% CI, 38.5-81.5) with any untreated lesions (*P* = .006). Median distant metastasis–free survival was 29.0 months in men with no untreated lesions at baseline and 6.0 months in men with any untreated lesions at baseline (HR, 0.19; 95% CI, 0.07-0.54; *P* < .001) (Figure 2D; eResults in Supplement 2).

No grade 3 or higher adverse events were identified (eTables 3 and 4 in Supplement 2). No differences in Brief Pain Inventory (Short Form) scores were observed between arms or within either arm across time.

**Figure 3.**
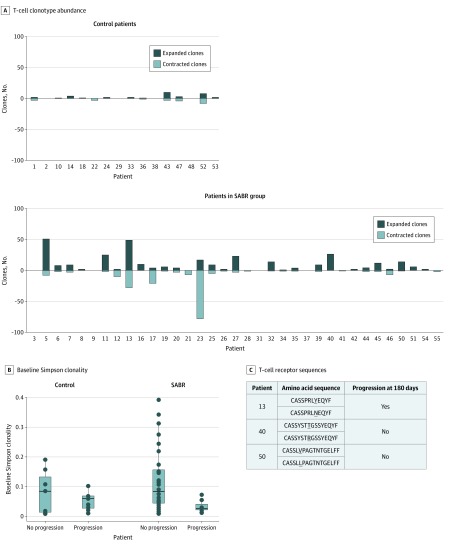
Baseline and Dynamic Immunologic Features Suggesting Interplay Between Stereotactic Ablative Radiotherapy (SABR) and the Immune System A, Changes in T-cell clonotype abundance at day 90 from baseline. B, Baseline Simpson clonality stratified by progression at 180 days. C, Clustered T-cell receptor sequences identified at day 90 in 3 patients treated with SABR.

Peripheral blood mononuclear cells were collected at baseline and day 90 from participants in both arms for deep sequencing of T-cell receptor DNA. Differential clonotype abundance appeared more pronounced in the SABR arm (Figure 3A), with significantly more expanded clones and a nonsignificantly greater amount of contracted clones at 90 days compared with observation. Greater peripheral baseline clonality was associated with composite end point progression at 180 days in participants receiving SABR (0.082085 vs 0.026051; *P* = .03) but not with observation (0.084299 vs 0.060002; *P* = .68) (Figure 3B). At baseline, no participant had clusters of similar expanded T-cell receptors within their repertoire, but at day 90, clusters of similar expanded T-cell receptors were identified in 3 participants, all in the SABR arm (Figure 3C).

Plasma and matched leukocyte DNA samples collected at baseline from 54 participants were profiled by the CAPP-Seq (cancer personalized profiling by deep sequencing) method for analysis of ctDNA. Nonsynonymous mutations were present in 20 participants (37%) with a mean of 1.3 mutations per participant and a median allele fraction of 0.25%. No significant differences in ctDNA concentration were noted between participants whose disease did or did not progress in either the SABR or observation arm (eFigure 2 in Supplement 2).

**Figure 4.**
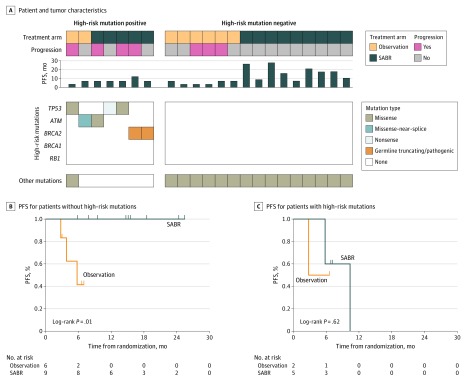
Association of High-Risk Mutation Status With Progression-Free Survival (PFS) After Stereotactic Ablative Radiotherapy (SABR) A, Patient characteristics and tumor mutations for patients with detectable circulating tumor DNA via CAPP-Seq or pathogenic germline mutations. B, PFS stratified by treatment arm for patients without high-risk mutations (n = 15). C, PFS stratified by treatment arm for patients with high-risk mutations (n = 7).

Based on prior sequencing studies^23,24,25^ that identified mutations associated with outcomes in metastatic prostate cancer, we defined a high-risk mutation signature with truncating/pathogenic germline mutations identified via a Color Genomics assay and confirmed by CAPP-Seq (Figure 4A; eTables 5 and 6 in Supplement 2). To avoid false negatives owing to undetectable ctDNA, we limited our analyses to participants with detectable ctDNA or truncating/pathogenic germline mutations in high-risk genes (n = 22). PFS was significantly longer among participants receiving SABR than among those in the observation arm in the high-risk mutation–negative subgroup (Figure 4B) but not in the high-risk mutation–positive subgroup (Figure 4C).

## Discussion

This phase 2 randomized clinical trial showed that among men with OMPC, those treated with SABR were significantly less likely to have disease progression than those undergoing observation alone. Local control for SABR-treated lesions was excellent, and the adverse effects associated with SABR were mild and did not appear to affect quality of life. These results are consistent with prior reports validating the existence of the oligometastatic state in prostate cancer and the utility of SABR as MDT in this condition.

With a median (interquartile range) follow-up of 3.0 (2.3-3.8) years, Ost et al^9^ reported a median ADT-free survival of 21 months (80% CI, 14-29 months) with SABR compared with 13 months (80% CI, 12-17 months) with observation (HR, 0.60; 80% CI, 0.40-0.90; log-rank *P* = .11). Criteria for initiation of ADT were defined as “symptomatic progression, progression to more than three metastases, or local progression of baseline-detected metastases.”^9^^(p448)^ Importantly, progression by PSA increase alone was not an indication to start ADT, nor was development of additional metastases amenable to MDT as long as the patient still had 3 or fewer total metastases.^9^ In the present cohort, 2 of 7 men with disease progression in the SABR arm and 7 of 11 men with disease progression in the observation arm experienced biochemical progression alone. Furthermore, additional SABR was the next intervention in 14 of 15 men in the observation arm who ultimately received subsequent treatment and 6 of 14 men in the SABR arm. These differences inform the limitations of direct comparison of these trials.

Another important consideration is that SABR in the STOMP trial^9^ included all concerning lesions identified by choline PET-CT. The ORIOLE trial enrolled participants with less-sensitive conventional imaging and still demonstrated a positive benefit for MDT, suggesting that the oligometastatic state is heterogeneous and that better biomarkers are needed to define participants who would benefit most from MDT. Post hoc analysis of PFS based on extent of disease appreciable by PSMA-targeted PET-CT found significant PFS and distant metastasis–free survival advantages among men who received consolidation of all detectable disease. These data support the use of molecular imaging in conjunction with MDT for patients with OMPC.

The key question that remains incompletely answered is whether we can alter the natural history of OMPC with MDT. Clearly, SABR is a safe and effective way to forestall progression of treated metastases and improves oncologic outcomes in certain patients.^6,7^ Furthermore, complete consolidation of detectable metastases improves time to progression. Most men with oligometastatic disease do not experience a complete PSA response after SABR, which suggests that residual micrometastases are present but undetectable. The consolidation of macroscopic disease may simply reset the clock on time to detectable metastases, and micrometastatic disease may continue to grow unchecked until it reaches sufficient size to become clinically actionable. Alternatively, consolidation of macroscopic metastases may remove or significantly affect signals that promote the development of remaining micrometastases. Our finding that total consolidation of disease detectable by PSMA-targeted PET-CT was associated with lower risk of new metastases at 6 months is consistent with this latter explanation, as is the recent overall survival improvement observed in the SABR-COMET trial.^7^ A deeper understanding of this process may be obtained through sequencing of biopsy or liquid biopsy specimens to explore the relationships and lineages of specific metastases in these patients^14,26^ or through advances in analysis of circulating readouts, such as circulating tumor cells, ctDNA, and exosomes.

Our analysis of ctDNA revealed several key findings. First, ctDNA concentrations in patients with OMPC were significantly lower than those reported in prior studies^17,27^ of more advanced metastatic castration-resistant or hormone-sensitive prostate cancer. This suggests that ultrasensitive strategies, such as tumor-informed ctDNA monitoring, will be required for reliable detection and monitoring of ctDNA in patients with OMPC. Second, we did not find an association of baseline ctDNA concentration with outcome. However, our analysis was limited by the small fraction of participants with detectable ctDNA, so further exploration in future cohorts using tumor-informed monitoring or alternative methods is warranted. Third, the results of the study suggest that the presence of mutations associated with worse prognosis may identify a subset of patients who do not benefit from MDT. If these findings are confirmed in independent cohorts, the absence of high-risk mutations could potentially serve as a predictive biomarker for benefit from MDT.

The benefit of early ADT initiation remains a controversial question,^28,29,30^ and rigorous evaluation of men who undergo multiple rounds of MDT rather than proceeding to systemic therapy at first progression may shed light on the effect of SABR on the natural history of this disease. If a single round of MDT arrests the progression of some but not all lesions, subsequent rounds of MDT might salvage the remaining disease until what remains is inadequate to support a metastatic phenotype. The utility of repeated MDT may also vary by patient and the response of individual; therefore, well-selected patients for MDT may have intrinsic predictive value for guiding subsequent management.

The effect of radiotherapy on the immune system is also an area of interest with the promise of using SABR to induce an in situ vaccine response.^20,31^ We observed enhanced differential clonotype expansion, clusters of similar expanded T-cell receptors, and a clinical benefit to greater baseline clonality seen only in participants treated with SABR. Future studies assessing the association of these findings with T-cell characteristics or relatedness to tumor-infiltrating lymphocytes may help further characterize this systemic immune response.

Soldatov et al^32^ described patterns of failure following PSMA-ligand–based, conventionally fractionated radiotherapy for OMPC and found that recurrences are bone trophic. This suggests a role for aggressive management of micrometastatic osseous disease with ADT and/or radium 223, the latter of which will be the center of investigation for the Radium-223 and SABR vs SABR for Oligometastatic Prostate Cancers (RAVENS) trial (ClinicalTrials.gov identifier: NCT04037358). Soldatov et al^32^ also found that 17% of recurrences after MDT were in pelvic nodes. The best management approach for pelvic recurrences is currently being studied in the Salvage Treatment of Oligorecurrent Nodal Prostate Cancer Metastases (STORM) trial (ClinicalTrials.gov identifier: NCT03569241).

### Limitations

While these results are promising, this trial is limited by its relatively small sample size; subsequent phase 3 validation would strengthen the argument in favor of this approach. Additionally, our ability to study the long-term implications of this treatment approach was limited by high rates of crossover occurring after the predefined 6-month primary end point, with 15 of 18 men randomized to observation ultimately seeking SABR.

It should also be noted that the correlative data presented herein are hypothesis generating and require further prospective validation. Although we have identified a systemic immune response to SABR, we do not yet understand the nature of this response, and additional studies are needed to better characterize the interactions between immune cells, tumor, and the microenvironment. A limitation of our ctDNA analysis was the lack of available biopsy specimens to confirm the presence or absence of mutations. Thus, although we sequenced matched leukocyte DNA to identify mutations owing to clonal hematopoiesis, it is possible that some of the mutations we detected did not originate from tumor cells. Future studies in this area should prioritize acquisition of tissue samples for molecular analysis.

## Conclusions

In conclusion, SABR is a safe and effective modality for MDT in OMPC that improves PFS compared with observation and results in a systemic adaptive immune response. Complete consolidation of metastatic disease detectable by molecular imaging decreases the risk of subsequent metastases, suggesting an alteration in the natural history. Finally, baseline immune phenotype and a tumor mutation signature may predict clinical response to SABR, pending validation in independent cohorts. Although SABR alone may or may not be sufficient as curative management, the combination of SABR with systemic therapies may provide the multipronged attack required to cure this disease.

## Notes

Supplement 1. Trial Protocol

Supplement 2. **eMethods.** **eResults.** **eFigure 1. **Total gross tumor volume in patients treated with SABR was not different in patients with or without progression at 180 days **eFigure 2. **Baseline circulating tumor DNA concentration is not associated with outcome **eTable 1. **Treatment volume characteristics for SABR arm **eTable 2. **Distribution of lesions by treatment arm **eTable 3. **CTCAE v4.0 grade 1 adverse events **eTable 4. **CTCAE v4.0 grade 2 adverse events **eTable 5. **Genes fully or partially covered in prostate cancer focused CAPP-Seq selector **eTable 6. **Mutations identified by CAPP-Seq in baseline plasma samples **eReferences.**

Supplement 3. Data Sharing Statement
