# Supplementary material for: Outcomes of Observation vs Stereotactic Ablative Radiation for Oligometastatic Prostate Cancer: The ORIOLE Phase 2 Randomized Clinical Trial
Source: JAMA Oncol. 2020 Mar 26;6(5):650–9. doi: 10.1001/jamaoncol.2020.0147 (PMC7225913; doi:10.1001/jamaoncol.2020.0147)
Supplement: Supplement 1. — Trial Protocol [file jamaoncol-6-650-s001.pdf]

Summary of IRB-approved amendments to ORIOLE protocol

April 29, 2016

- Added rectal swab for microbiome analysis
- Updated dates of correlative blood draws
- Moved testosterone from day 1 to pre-study (within 6 months prior to registration)
- Increased allowed time frame for previous ADT
- Corrected pregnancy statement
- Added language to allow replacement if patients drop out
- Changed title of protocol

July 6, 2016

- editing cover page; clarifying dates to be from randomization; edits to the study calendar to be consistent with protocol; sections 6.8, 6.10, 6.11, and 6.13 of the protocol
- changing version and date of the protocol
- edited section 15.9 of the application
- adding Sibley and Suburban as participating sites.

Nov 30, 2016

- Changes in Table of Contents (addition of Color Genomics and formatting)
- Removal of Cell Search from Schema and Calendar
- Separation of SBRT and Observation Arm Calendars
- The Removal of CTC-Cell Search information from entire Protocol
- Addition of Color Genomics Testing/Color saliva test (Rational, Collection and processing)
- Color Saliva Collection step-by-step in appendix
- Spelling and Grammatical Changes
- Correction and clarification in therapeutic Procedures
- Addition of details into follow-up procedures as per IRB
- Change in including patients with systemic therapy (inclusion)
- Change in having previous DCFPyL PET/MRI and/or DCFPyL PET/CT prior to enrollment (exclusion criteria)

March 13, 2017

- Removed Charles Drake from cover page
- Clarified Study Calendars regarding labs--pre-study timing

June 21, 2017

- Added 90 Day Rectal Swab on Schema and Calendar for both SBRT and Observation Arms

August 9, 2017

- Edited Schema: Making it easier to read and incorporating the changes below
- Added DCFPyL-PET Day 1 and Day 180 to observation arm (Schema, Protocol, and Calendar) - last 10 enrolled
- Added ImmunoSEQ to Day 1 and Day 90 of Calendar and Schema - first ten enrolled

Phase II Randomized Study of Stereotactic Body Radiation Therapy for Prostate Cancer Oligometastases

Protocol Number	J15180
IND Number	# 121,064

Principal Investigator	Phuoc T. Tran, M.D., PhD<br>Sidney Kimmel Comprehensive Cancer Center<br>Johns Hopkins University<br>Weinberg Building<br>1150 Orleans St., CRB 2 Rm 406<br>Baltimore, MD 21231<br>Phone: 410-614-3880<br>Fax: 410-502-1419<br>Email: tranp@jhmi.edu
Co-Investigators	Theodore DeWeese, M.D.<br>Danny Song, M.D.<br>Mario Eisenberger, MD<br>Ashley Ross, MD, PhD<br>Michael Gorin, MD<br>Michael Carducci, MD<br>Charles Drake, MD, PhD<br>Emmanuel Antonarakis, MD<br>Channing Paller, MD<br>Kenneth Pienta, MD<br>Lawrence Kleinberg, M.D.<br>Kristin Redmond, M.D.<br>Steve Rowe, MD, PhD<br>Martin Pomper, MD, PhD
Statisticians	Hao Wang, PhD<br>Bruce Trock, PhD
Research Nurse	Kelly Szanjna
Research Managers:	Helen Kim<br>Terry Caldwell
Study Coordinators:	Megan Kummerlove

Funding:	NCI 1U01CA183031-01A1 (Pomper/DeWeese)<br>Movember-PCF Challenge Award (Tran/Ross/Dicker)

Table of Contents

SCHEMA......4

1. PROTOCOL SYNOPSIS......5

2. ABBREVIATIONS AND DEFINITIONS OF TERMS......7

3. STUDY DESIGN AND OBJECTIVES......8

3.1 STUDY DESIGN......8
3.2 PRIMARY OBJECTIVE......8
3.3 SECONDARY OBJECTIVES......8
3.4 CORRELATIVE OBJECTIVES......8

4. BACKGROUND......9

4.1 OLIGOMETASTATIC DISEASE......9
4.2 STEREOTACTIC BODY RADIATION THERAPY (SBRT/SABR)......9
4.3 RATIONALE FOR USE OF SBRT/SABR IN OLIGOMETASTATIC DISEASE......10
4.4 RATIONALE FOR SBRT/SABR FOR BONE OLIGOMETASTATATASES IN PROSTATE CANCER......11
4.5 RATIONALE FOR PSMA FUNCTIONAL IMAGING TO REFINE SBRT IN PROSTATE CANCER......11
4.6 RATIONALE FOR CORRELATIVE SCIENCE......14

5. PARTICIPANT SELECTION AND ENROLLMENT PROCEDURES......18

5.1 INCLUSION CRITERIA......18
5.2 EXCLUSION CRITERIA......19

6. TREATMENT PLAN......19

6.1 RANDOMIZATION......19
6.2 DIAGNOSTIC PROCEDURES......20
6.3 THERAPEUTIC PROCEDURES......20
6.4 FOLLOW-UP PROCEDURES......23
6.5 DURATION OF THERAPY......23
6.6 DURATION OF FOLLOW-UP......23
6.7 CRITERIA FOR PATIENT REMOVAL......23
6.8 ALTERNATIVES......24
6.9 COSTS......24
6.10 COMPENSATION......24
6.11 WITHDRAWAL FROM STUDY......24
6.12 CROSS-OVER......24

7. INVESTIGATIONAL AGENT/DEVICE/PROCEDURE INFORMATION......24

7.1 INVESTIGATIONAL AGENT......24

8. STUDY CALENDAR......25

9. MEASUREMENT OF EFFECT......27

9.1 ANTITUMOR EFFECT-SOLID TUMORS......28
9.2 DEFINITIONS......28
9.3 DISEASE PARAMETERS......28
9.4 METHODS FOR EVALUATION OF MEASURABLE DISEASE......29
9.5 RESPONSE CRITERIA......29
9.6 IMAGING REFERENCE STANDARD......29

10. ADVERSE EVENT REPORTING REQUIREMENTS......30

10.1 GENERAL......30
10.2 DEFINITIONS......31

10.3 POTENTIAL ADVERSE EVENTS 32
10.4 SBRT/SABR 32
10.5 DCFPYL 33
10.6 REPORTING PROCEDURES 36

11. DATA AND SAFETY MONITORING PLAN 39

12. REGULATORY CONSIDERATIONS 40

12.1 PROTOCOL REVIEW AND AMENDMENTS 40
12.2 INFORMED CONSENT 40
12.3 ETHICS AND GCP 41

13. STATISTICAL CONSIDERATIONS 40

13.1 ENDPOINTS 40
13.2 SAMPLE SIZE/ACCRUAL RATE 41
13.3 EARLY STOPPING GUIDELINES: 41
13.4 ANALYSIS OF PRIMARY OBJECTIVE 42
13.5 ANALYSIS OF SECONDARY OBJECTIVES 42
13.6 EVALUATION OF TOXICITY 43
13.7 CORRELATIVE SCIENCE 43

REFERENCES: 44

APPENDIX I: BRIEF PAIN INVENTORY FORM 48
APPENDIX II: PERFORMANCE STATUS 50
APPENDIX III: CAPP-SEQ SAMPLE COLLECTION & PROCESSING 51
APPENDIX IV: EPIC HD-CTC SAMPLE COLLECTION & PROCESSING 52
APPENDIX V: IMMUNOSEQ SAMPLE COLLECTION & PROCESSING 54
APPENDIX VI: SKCCC DSMP 56

SCHEMA

Eligibility
Men with histologically confirmed oligometastatic (≤3 mets) hormone-sensitive prostate cancer and the following:
PSADT <15 mos, tumors ≤ 5.0 cm or <250 cm^3, Age ≥18 yrs & ECOG ≤ 2

↓

Randomize 1:2
Observation (n=18) *versus* SBRT (n=36)

↓

Day 1: DCFPyL-PET/MRI or -PT/CT

↓

Day 1: Blood draws for PSA, LDH, AP & testosterone *versus*
PSA, LDH, AP, testosterone & CTCs/ctDNA assays, PBMCs (ImmunoSEQ)

↓

Day 1-30: Observation *versus* **SABR to 1-3 mets**

↓

Day 90: Blood draws for PSA, LDH, AP & testosterone *versus*
PSA, LDH, AP, testosterone & CTCs/ctDNA assays, PBMCs (ImmunoSEQ)

↓

Day 180: Bone Scan & CT *versus*
Bone Scan, CT & DCFPyL-PET/MRI or -PT/CT

↓

Day 180: Blood draws for PSA, LDH, AP & testosterone *versus*
PSA, LDH, AP, testosterone & CTCs/ctDNA assays

↓

Patients that progress in the Observation arm can be crossed over off-protocol to receive SBRT.
All other patients can continue with standard of care.

1. PROTOCOL SYNOPSIS

TITLE	Phase II Randomized Study of Stereotactic Body Radiation Therapy for Prostate Cancer Oligometastases
STUDY PHASE	Phase II
INDICATION	Men with oligometastatic disease ($\leq$3 mets) and PSADT <15 months
PRIMARY OBJECTVES	• Proportion of men who have progressed after 6 months from randomization to observation *versus* stereotactic body radiation therapy (SBRT) who have oligometastatic prostate cancer
SECONDARY OBJECTIVES	• To assess the toxicity of SBRT in patients with oligometastatic disease<br>• To determine local control at 6-months after SBRT in patients with oligometastatic disease<br>• To assess progression free survival after randomization to observation *versus* SBRT<br>• To assess ADT-free survival after randomization to observation *versus* SBRT<br>• To assess quality of life following control *versus* SBRT arm<br>• Estimate the proportion of metastatic lesions found on bone scan that are DCFPyL-PET/MRI or –PET/CT positive and vice versa.<br>• Estimate the proportion of DCFPyL-PET/MRI or –PET/CT positive sites that are positive for new or progressive metastatic disease by bone scan at 6-months from SBRT and vice versa
CORRELATIVE SCIENCE	• To enumerate circulating tumor cells (CTC) using CellSearch CTC™ and EPIC HD-CTC platforms at baseline, day 90 and day 180 from SBRT.<br>• To enumerate circulating tumor DNA (ctDNA) using Cancer

	Personalized Profiling by deep sequencing (CAPP-Seq) at baseline, day 90 and day 180 from SBRT.<br>• To quantitatively sequence T-cell receptor (TCR) repertoires using peripheral blood monocytes and the ImmunoSEQ platform at baseline and day 90 from SBRT.<br>• Descriptive statistics will be performed to correlate the metrics above with clinical outcomes.
HYPOTHESES	SBRT will reduce progression at 6-months from ≥80% in the control arm to ≤40% in the SBRT arm
STUDY DESIGN	Men with oligometastatic prostate cancer lesions will be randomized (1:2) to observation versus SBRT. The study will NOT be blinded. Within three weeks of the initial treatment planning, SBRT (1-5 fractions) will be administered.
SAMPLE SIZE BY TREATMENT GROUP	54 patients total
SUMMARY OF SUBJECT ELIGIBILITY CRITERIA	1.Metastatic bone or nodal sites ≤ 3.<br>2.PSADT <15 months<br>3.Tumor ≤ 5.0 cm or <250 cm3.<br>4.Age ≥ 18 years.<br>5.ECOG perfomance status ≤ 2.<br>6.Histologic confirmation of malignancy (primary or metastatic tumor).
CONTROL GROUP	N/A
PROCEDURES	1. Physical exam<br>2. CT/MRI scan of Involved Site<br>3. Bone scan<br>4. Randomization<br>5. DCFPyL-PET/MRI or –PET/CT<br>6. Blood draws<br>7. Observation *versus* SBRT<br>8. Blood draws<br>9. DCFPyL-PET/MRI or –PET/CT, bone scan and CT/MRI of Involved Site

2. ABBREVIATIONS AND DEFINITIONS OF TERMS

ADL	Activities of daily living
AE	Adverse event
BID	Twice daily
BSA	Body surface area
CBC	Complete blood count
CI	Confidence interval
CMAX	Maximum concentration of drug
CNS	Central nervous system
CRF	Case report/Record form
CR	Complete response
CRPC	Castration-Resistant Prostate Cancer
CTC	Circulating Tumor Cell
CTCAE	Common Terminology Criteria for Adverse Events
DLT	Dose Limiting Toxicity
DSMB	Data Safety Monitoring Board
ECG	Electrocardiogram
GI	Gastrointestinal
Hgb	Hemoglobin
HIV	Human Immunodeficiency Virus
HPF	High-power field
HTN	Hypertensions
IRB	Institutional Review Board
IV	Intravenous
LLN	Lower limit of normal
MTD	Maximum tolerated dose
OS	Overall survival
PLT	Platelet
PD	Progressive diseased
PFS	Progression free survival
PR	Partial response
PSADT	PSA Doubling Time
QD	Once daily
RECIST	Response evaluation criteria in solid tumors
RR	Response rate
SABR	Stereotactic ablative radiation therapy
SAE	Serious adverse event
SBRT	Stereotactic body radiation therapy
SD	Stable disease
TTP	Time to progression
ULN	Upper limit of normal
UNK	Unknown
WBC	White blood cell

3. STUDY DESIGN AND OBJECTIVES

3.1 Study Design

Phase II non-blinded randomized study evaluating men with oligometastatic prostate cancer lesions randomized (1:2) to observation versus stereotactic body radiation therapy (SBRT).

3.2 Primary Objective

To determine the proportion of men who have progressed after 6 months from randomization to observation *versus* SBRT who have oligometastatic prostate cancer.

3.3 Secondary Objectives

To assess the toxicity of SBRT in patients with oligometastatic disease.

To determine local control at 6-months after SBRT in patients with oligometastatic disease.

To assess progression free survival (PFS) after randomization to observation *versus* SBRT.

To assess ADT-free survival after randomization to observation *versus* SBRT.

To assess quality of life following completion of SBRT.

Estimate the proportion of metastatic lesions found on bone scan/CT that are DCFPyL-PET/MRI positive and vice versa.

Estimate the proportion of DCFPyL-PET/MRI or –PET/CT positive sites that are positive for new or progressive metastatic disease by bone scan/CT at 6-months following SBRT and vice versa.

3.4 Correlative Objectives

To enumerate circulating tumor cells (CTC) using CellSearch CTC™ and EPIC HD-CTC platforms at baseline, day 90 and day 180 from SBRT.

To enumerate circulating tumor DNA (ctDNA) using Cancer Personalized Profiling by deep sequencing (CAPP-Seq) at baseline, day 90 and day 180 from SBRT.

To quantitatively sequence T-cell receptor (TCR) repertoires using peripheral blood monocytes and the ImmunoSEQ platform at baseline and day 90 from SBRT.

Descriptive statistics will be performed to correlate the metrics above with clinical outcomes

4. BACKGROUND

4.1 Oligometastatic Disease

Cancer is the second leading cause of death in the United States, chiefly from an inability to control metastatic disease. Systemic therapy alone is not curative for patients with most metastatic solid tumors.(1) The metastatic capacity of cancers behaves along a spectrum of disease progression, such that some tumors have spread widely before clinical detectability and others never metastasize. Contained within this spectrum, is an oligometastatic state where metastases are limited in number and location. The presence of an oligometastatic state was originally proposed by Hellman who suggested that these oligometastatic patients would benefit from effective local therapy in addition to systemic therapy.(1) In agreement with this hypothesis, surgery and chemotherapy for isolated pulmonary metastases can result in long term disease-free periods. (2) Additionally, some 25% of patients following resection and chemotherapy for colorectal cancer and isolated liver metastases can similarly have long-term disease free survival.(3)(4)(5)

The treatment of metastases depends on multiple factors including 1) the location of the primary tumor, 2) the presence or absence of other metastatic foci, 3) the size, number and location of metastases, 4) the effectiveness of various forms of therapy (such as surgery, radiation and chemotherapy), and 5) patient's functional status. Extracranial metastatic tumors most often referred for local therapy in the form of resection include colon cancer, sarcomas, germ cell tumors, and melanoma metastatic to the lung. For the more common primary tumors such as those of breast and prostate, metastases are rarely referred for resection as chemotherapy (or hormonal manipulation) is generally considered to be the primary mode of treatment.

4.2 Stereotactic Body Radiation Therapy (SBRT) or Stereotactic Ablative Radiation Therapy (SABR)

Conventional moderate dose radiation for metastatic disease is given primarily for palliation. Recent advancements in radiation delivery now make it possible to image and treat precisely within any anatomical region of the body.(6, 7) As a result, the capacity to deliver tumor killing radiation doses in a single or few (1-5) outpatient radiation treatments is now possible.(8-12) In addition, by minimizing the irradiation of surrounding healthy tissue, it should also be possible to decrease the rate of complications. Intracranial stereotactic body radiation therapy (SBRT) [also known has stereotactic ablative radiation therapy (SABR)] has been shown to be a highly effective treatment for brain metastases.(13) Data suggests that selective small extracranial tumors (either primary or metastatic tumors) may be effectively controlled by similar focal high-dose SBRT/SABR. There is an increasing experience with extracranial SBRT as effective local therapy for metastatic lesions. Local control in excess of 75% has been reported for metastatic tumors of the spine, lung and liver, which is significantly higher than standard conventional moderate dose radiation.(9, 11,

12, 14)(15-27) Toxicity has been minimal in multiple U.S., European and Japanese trials of extracranial stereotactic radiotherapy to the lung, liver, spine, pelvis and abdomen despite the use of very high biological equivalent doses for patients with both organ confined and metastatic cancer.

4.3 Rationale for Use of SBRT/SABR in Oligometastatic Disease

Historically, aggressive local therapy of metastases has not been fruitful secondary to further progression from microscopic metastatic deposits. Chemotherapy and even molecular-targeted agents rarely eradicate macroscopic metastases permanently. However, as systemic treatments for microscopic metastatic disease have improved the importance of local therapy in metastatic disease has been re-examined. It is now recognized that some patients with “oligo,” or few sites of metastases, may have isolated sites of metastases that can be potentially eradicated with aggressive local therapy. The term “oligometastases” was coined to refer to this stage of distant metastases. Typically, the entire burden of disease can be recognized as a finite number of discrete lesions. Although there is no strict definition of oligometastases, it is commonly interpreted to be no more than 5 or 6 metastatic sites.

Milano et al. (9)from the University of Rochester have also shown that the net tumor burden for patients with 5 or fewer sites of macrometastatic disease, treated on 2 prospective SBRT protocols, was an independent predictor of overall outcome. Whether SBRT, as an adjunct to systemic therapy, to selected macroscopic metastases can influence overall survival by keeping the burden of disease below such a “lethal threshold” is being investigated.

Colorectal carcinoma metastatic to the liver, selected modern resection series have yielded 5-year survival rates of approximately 50% showing that improved systemic treatments and local therapy has the potential to cure “oligo” or isolated liver metastases(5, 9, 28-31). The benefit of local therapy in non-colorectal liver metastases is less clearly defined, but long-term survival has been reported after the resection of liver metastases from sarcoma, breast cancer, and other tumor sites(32).

Goodman et al.(15) reported a phase I dose-escalation single-fraction trial for patients with liver metastases or intrahepatic cholangiocarcinoma. Doses were escalated in 4-Gy cohorts from 18 Gy up to 30 Gy in 1 fraction. Twenty-six patients with 40 lesions were treated. There was no dose-limiting toxicity. The median follow-up was 17 months, and this corresponded to a 12-month local control rate of 77%. The 2-year actuarial survival rate was 50.4%.

4.4 Rationale for SBRT/SABR for Bone Oligometastases in Prostate Cancer

Major advancements in radiation treatment planning and delivery have resulted in resurgence in the use of radiation therapy (RT) as a treatment for bone metastases. SBRT/SABR is defined as highly focused, stereotactic localized,

high-dose RT delivered in a hypofractionated course. In selected patients, very high local control rates have been observed, with minimal toxicity. Bone metastases represent the major metastatic site (>90%) in men with rising PSA following primary treatment for their prostate cancer.

The primary management of metastatic prostate cancer is systemic therapy in the form of androgen deprivation therapy (ADT). Many men can remain on ADT treatment for years before progression or failure of ADT. However, similar to chemotherapy for other metastatic malignancies, ADT and even newer androgen receptor signaling inhibitory agents rarely eradicate metastatic disease permanently. In addition, ADT has been shown conclusively to adversely affect patient quality of life. Thus, even the ability to defer ADT initiation in men with oligometastatic prostate cancer represents a considerable clinical advance.

On the basis of this emerging clinical evidence and because SBRT for bone metastases is known to be safe, we propose a phase II study of SBRT in patients with oligometastatic prostate cancer. Abundant experience with SBRT for bone metastases provides useful safety information for our trial. We are also building off of our Phase II SBRT trial in diverse histologies here at Johns Hopkins - J12137. This trial has almost completed accural to the target of 42 patients, but we have already finished accuring the 20 prostate cancer patient limit. Although very preliminary we have seen only low G1-G2 toxicites and no local failures to date in our prostate cancer patients. Thus the proposed study represents an informed estimate based on current knowledge of SBRT doses and those administered in currently approved image-guided protocols including our own JHU protocol (brain, base of skull, cervico-thoracic spine, pancreas and liver).

In general metastatic disease carries an extremely high mortality rate. Current therapies provide only partial palliation of symptoms and mild to moderate prolongation of survival. Patients are rarely cured of this disease; consequently, better treatment is clearly needed. The proposed treatment represents a logical extension of the current state-of-the-art radiation therapy. It has the potential to translate into more effective palliation and longer patient survival free of intiation of systemic treatment.

4.5 Rationale for Prostate specific membrane antigen (PSMA) Functional Imaging to Help Refine Selection of Bone Lesions to Target with SBRT/SABR in Oligometastatic Prostate Cancer

Conventional imaging modalities, *i.e.,* bone scintigraphy, CT and MR imaging, are currently used to detect metastatic prostate cancer for staging.(4) Positron emission tomography (PET) imaging, particularly [^{18}F]fluorodeoxyglucose positron emission tomography ([^{18}F]-FDG PET), has gained an important role in the clinical management of cancer patients, more notably for staging and

assessment of response to therapy.(32, 33) However, studies employing [^{18}F]-FDG PET have demonstrated low uptake in prostate cancer except for advanced metastatic disease.(34-37) A number of novel PET radiotracer are being investigated for use in prostate cancer but have yielded mixed results and have yet to gain widespread clinical use.(38, 39) [^{11}C]Choline is the most widely studied PET radiotracer for prostate cancer detection and has demonstrated the ability to detect lymph node and bone metastases.(40-42) [^{11}C]Acetate is another emerging radiotracer, which has been evaluated in a limited number of studies and appears to demonstrates comparable uptake in comparison to [^{11}C]Choline for detection of prostate tumor and metastases.(43) One limitation of these radiotracers include nonspecific uptake at sites of inflammation. ^{18}F-Fluoride-PET (NaF-PET) for identifying bone metastases has proven very sensitive but is unable to differentiate between viable metastatic prostate tumor from chronic reactive bone changes.(44, 45) Other promising radiopharmaceuticals for prostate cancer include anti-1-amino-3-^{18}F-fluorocyclobutane-1-carboxylic acid (^{18}F-FACBC) and ^{18}F-fluorodihydrotestosterone (^{18}F-FDHT), which are also actively undergoing clinical evaluation in a variety of settings.(46-48) Further work needs to be done to compare the merits of [^{11}C]Choline and other emerging PET radiotracers in the detection and management of prostate cancer.(49)

Prostate specific membrane antigen (PSMA is a promising well-characterized biomarker specific for prostate cancer which has also been associated with prostate tumor aggressiveness. Histologic studies have associated high PSMA expression with metastatic spread (50-52), androgen independence (53), and expression levels have be found to be predictive of prostate cancer progression(54, 55). However, previous attempts to image PSMA by single-photon-emission computed tomography (SPECT) (Prostacint^{TM}) demonstrated poor performance due to inherent limited antibody and imaging characteristics (poor tumor penetration, slow blood pool clearance, low SPECT resolution).(56, 57)

[^{18}F]DCFBC (N-[N-[(S)-1,3-Dicarboxypropyl]Carbamoyl]-4-[18F]Fluorobenzyl-L-Cysteine) (DCFBC) is a promising clinically practical small molecule PET imaging agent specific for prostate cancer with superior pharmacodynamic and pharmacokinetic characteristics than existing prostate cancer imaging agents. This is a small molecule urea-based analog inhibitor of PSMA radiolabeled with a PET radiotracer fluorine-18 which was rationally designed based on knowledge of the crystal structure of PSMA. (58, 59) DCFBC is a high affinity inhibitory ligand for the prostate specific membrane antigen (PSMA). The relative affinity of DCFBC for PSMA was determined by evaluating ability of DCFBC to inhibit the N-acetylaspartylglutamate (NAAG) peptidase activity of PSMA using a previously developed NAAG peptidase assay. The IC50 value for the inhibitory capacity of DCFBC for PSMA was 13.9 nM, as determined by a NAAG peptidase inhibition assay, in keeping with other compounds of this class. Our proposed work is innovative because DCFBC PET imaging will optimize functional PSMA prostate

cancer imaging by improving the following factors: (1) as a small molecule it allows for higher tumor penetration and rapid blood clearance allowing for increased tumor to background ratio, (2) it targets the more accessible external binding domain of PSMA, and (3) PET imaging allows for quantitative high resolution images, and (4) clinically practical labeling with ^{18}F, with a 2-hour half-life. PSMA-specific uptake of this radiotracer have been validated in PET imaging of pre-clinical human prostate cancer xenograft models which verify high selective uptake in PSMA expressing prostate tumors but no uptake in prostate tumors not expressing PSMA.(60, 61) Small animal PET imaging of DCFBC in subcutaneous prostate cancer bearing mice demonstrated specific uptake in PSMA expressing PC-3 PIP tumors, achieving a maximum target to background (muscle) ratio of 20:1 at 120 min after injection. The time-activity curve demonstrates that DCFBC has achieved equilibrium by 120 minutes and has begun to decrease in concentration at the target site, with washout from target sites slower than non-target sites.

Preliminary results of a recently completed Phase I first-in-man biodistribution and dosimetry study of DCFBC in five patients with advanced metastatic disease demonstrated high radiotracer uptake in both nodal and bone metastatic sites, minimal radiotracer metabolism, and favorable biodistribution.(62) Of 32 total sites of DCFBC uptake, 21 were concordant with prostate cancer metastases as determined by conventional imaging modalities (CIM) (CT, bone scan) at 21 sites and the other 11 sites of DCFBC uptake not detected by CIM were unconfirmed but considered suspicious for metastases. Seven bone findings thought to be benign fracture or stable changes on bone scan were negative for DCFBC uptake. Radiotracer administration was found to be safe with no severe adverse events. Tumor-to-background ratio for metastatic detection was highest at 2 hours post-radiotracer injection. A 2-hour post-injection DCFBC PET/CT with anterior MIP image demonstrating multiple pelvic bone metastases and sagittal PET, CT, and fused PET/CT images demonstrating T12 and L4 bone metastases (SUVmax 4.7 and 3.4, respectively). A 2-hour post-injection DCFBC transaxial PET/CT in another patient with prominent high level DCFBC uptake in a large right external iliac (SUVmax 11.6) and smaller left common iliac (SUVmax 5.3) pelvic nodal metastases. Dosimetry studies demonstrates the highest mean absorbed dose per unit administered activity (µGy/MBq) was to the bladder wall (32.4) and the mean effective dose (ED ± StdDev) was 19.9 ± 1.34 µSv/MBq, with dose estimates for our DCFPyL PET radiopharmaceutical that are comparable to those of other PET radiopharmaceuticals such as ^{18}F-fluorodeoxyglucose. Although further studies are needed for validation, our findings demonstrate the potential of DCFBC-PET imaging as a new positron-emitting imaging agent for detection of metastatic prostate cancer.

2-(3-{1-carboxy-5-[(6-[^{18}F]fluoro-pyridine-3-carbonyl)-amino]-pentyl}-ureido)-pentanedioic acid, DCFPyL, is a second generation compound that includes the pyridine moiety intended to improve pharmacokinetics through altered lipophilicity and potential pH dependent intra-tumoral sequestration. Extensive study of both

DCFBC and DCFPyL in pre-clinical models has been performed and published, attesting to their suitability for clinical translation – including with respect to dosimetry. While the first generation compound (DCFBC) provided isogenic PSMA+ human PC3 PIP tumor to PSMA- PC3 flu tumor ratios of 20:1 at 3 h post-injection, DCFPyL demonstrated ratios on the order of 400:1. Neither compound demonstrates appreciable de-fluorination *in vivo* and PSMA+ PIP to bone ratios are sufficient to enable imaging of bone metastases, as demonstrated below for DCFBC. After synthesis of ~100 compounds, including those with several new scaffolds designed to target PSMA, DCFPyL emerged as the best *in vivo* (63). On the basis of this promising clinical evidence with DCFBC-PET and the superiority of DCFPyL pre-clinically and because bone scintigraphy and NaF-PET are non-specific, we propose as a secondary objective evaluating the performance of DCFPyL-PET versus the conventional bone scan as a means to identify bone lesions for SBRT treatment in oligometastatic prostate cancer.

4.6 Rationale for Correlative Science

Novel Models of Metastasis and Circulating Tumor Cell (CTCs). Older models of metastasis portray the unidirectional flow of circulating tumor cells (CTCs) leaving the primary tumor and seeding a metastasis at a distant site(64). However, recent preclinical data using diverse experimental models of breast cancer, colon cancer and melanoma suggest metastasis is a multidirectional process where CTCs seed both distant sites as well as the original primary tumor – a process termed “self-seeding”(65, 66). Proponents of self-seeding have posited that CTC self-seeding of established macroscopic tumor sites likely requires less or no adaptation of CTCs to the recipient microenvironment in comparison to the colonization of CTCs to a distant and foreign site. In addition, self-seeding CTCs have already undergone selection for movement into and out of the circulation as well as resistance to anoiksis. Pre-clinical data have shown that self-seeding CTCs home back and extravasate into the primary in reaction to signals from the recipient primary tumors cells and tumor stroma. These self-seeding CTCs appear to be the most aggressive fraction of the CTC population(65). This feed forward loop of increasingly more aggressive cancer cells interacting with the tumor stroma results in the release of signals that foster tumor growth, angiogenesis, immune evasion, and ultimately macroscopic metastases.

Interestingly, genomic lineage tracing data of metastases from a rapid autopsy series of men who died of metastatic prostate cancer suggest macroscopic metastases represent *communal sanctuaries* that are composed of prostate cancer cells from many other metastatic sites throughout the body of patients(67). These *communal sanctuaries* are favorable niches that allow prostate cancer cells the ability to gain competence for the development of future macroscopic metastases. These human data are consistent with the preclinical concept of “self-seeding” or a multidirectional flow of CTCs. If these provocative data hold true, then SBRT/SABR to all macroscopic metastases in

oligometastatic patients may eliminate these *sanctuaries* and alter the natural history of metastatic patients. Following change in CTC numbers and biology following SABR may allow us to interrogate this hypothesis. The CellSearch CTC™ system is an FDA approved biomarker to allow enumeration of CTCs that can be ordered from national laboratories and is partially reimbursed by Medicare. Interventions that effectively target these macroscopic metastatic sanctuaries, such as SBRT/SABR, in combination with immune mediated therapies appear poised to arrest self-seeding and subsequent maturation of macroscopic metastases.

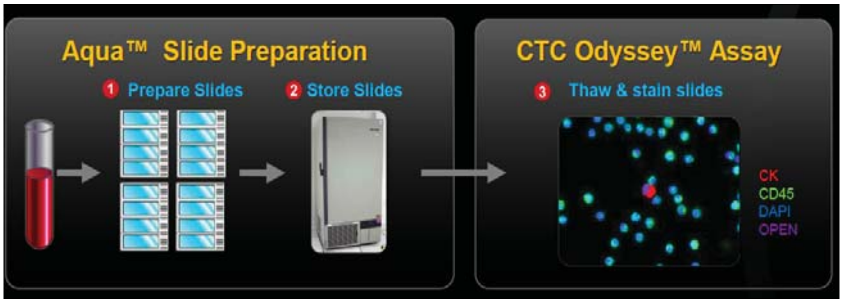

Fig. 1. The HD-CTC assay. Red blood cells are lysed followed by plating of nucleated cells on custom made cell-adhesion glass slides. Slides are stained for cytokeratin (CK), CD45 and DAPI, then CTCs are identified among leukocytes using computerized high resolution immunofluorescence imaging.

EPIC High-Definition analysis of single CTCs. The High Definition-CTC (HD-CTC) method can be used for the longitudinal CTC enumeration and to assess for AR expression(68). The assay employs as little as 1 mL of blood and is an unbiased protocol to distinguish CTCs among the surrounding leukocytes based on their cytokeratin positive (CK+) phenotype by using a high resolution immunofluorescence imaging. All cells are captured, and AR- cells can be evaluated. In addition, the HD-CTC technology preserves the cell morphology in such a way that enables the morphometric and the indirect quantification of AR and CK protein expression levels for all the CTCs identified in the blood sample (**Fig. 1**). Recently, the HD-CTC method has been used to examine expression of immune checkpoint molecules relevant to our proposal such as programmed cell death-1 (PD-1) on CTCs (data not shown). The HD-CTC assay was technically validated with cell line spiking experiments to reach an R^2=0.9997 on linearity testing as previously reported. These experiments were performed using SK-BR-3 cell lines and 0 to 3x10^2 cells per mL of normal donor control blood. The coefficient of variation is 16% and inter-processor correlation is R^2 = 0.979. Sample preparation process adhered to standard operating procedures for patient samples through a bar coded system for all consumables and instrumentation. All off-the-shelf instrumentation was calibrated according to analytical validation protocols established during commissioning.

Cancer personalized profiling by deep sequencing (CAPP-Seq) to detect circulating tumor DNA. Circulating tumor DNA (ctDNA) is a promising biomarker for non-invasive assessment of cancer burden, but existing ctDNA detection methods have either insufficient sensitivity and/or lack broad clinical applicability. Dr. Max Diehn's laboratory recently published Cancer Personalized Profiling by deep sequencing (CAPP-Seq) (69), an economical and ultrasensitive method for quantifying ctDNA. CAPP-Seq was initially implemented for lung cancer with a design covering multiple classes of somatic alterations that identified mutations in >95% of tumors. We detected ctDNA in 100% of patients with stage II–IV lung cancer and in 50% of patients with stage I, with 96% specificity for mutant allele fractions down to ~0.02%. Levels of ctDNA were highly correlated with tumor volume and distinguished between residual disease and treatment-related imaging changes, and measurement of ctDNA levels allowed for earlier response assessment than radiographic approaches from 1.5 mL of blood (**Fig. 2**). We have designed a prostate cancer-specific CAPP-Seq selector that identifies at least 1 mutation in >95% of prostate tumors with a median of ~4 mutations per tumor. We will apply CAPP-Seq to detect and monitor prostate cancer response to SBRT/SABR.

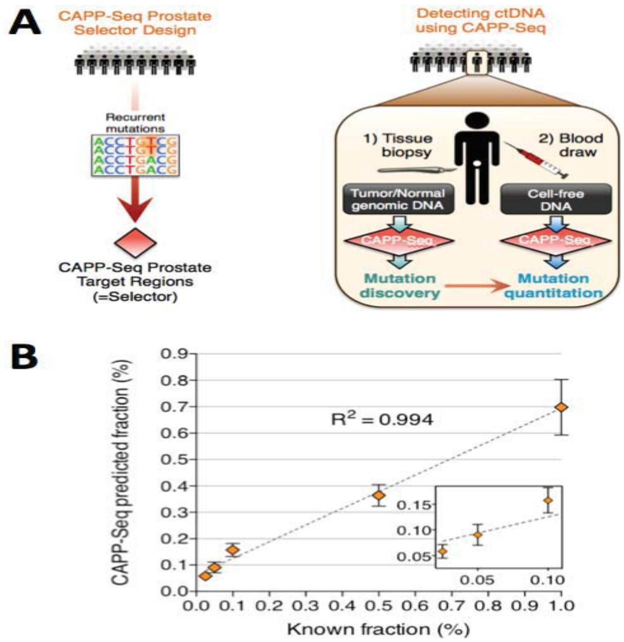

Fig. 2. CAPP-Seq ctDNA detection. (**A**) Analysis of exome sequencing from many tumors is used to select mutated genomic regions for a custom library of biotinylated oligonucleotides that are used for hybrid capture selection of tumor and germline genomic DNA and ctDNA. (**B**) Dilution series analysis of expected versus observed frequencies of mutant alleles, assessed by spiking fragmented HCC78 DNA into control cell free DNA.

ImmunoSEQ allows Unprecedented Profiling of the Anti-Tumor Immune Response. There are now an established body of pre-clinical and emerging clinical literature demonstrating that SABR can profoundly modify anti-tumor immune responses. Radiation induced activation of antigen presenting cells has been demonstrated in animal models to enhance tumor antigen cross-presentation in the draining lymph node and result in activation and proliferation of tumor specific cytotoxic T-cells (78). The cellular adaptive immune system generates a remarkable breadth of diversity in antigen-specific TCRs by combinatorial recombination of gene segments in lymphocytes. The TCR is composed of two peptide chains, encoded by the TCRA and TCRB or TCRG and TCRD genes, respectively. There are thus two types of T-cell receptors, αβ and γδ, that differ by the TCR heterodimer type and immune function. The antigenic specificity of T-lymphocytes is in large part determined by the amino

acid sequence in the hypervariable complementarity-determining region 3 (CDR3) regions of the T-cell receptors. Because of the potential diversity of receptors (a healthy adult has approximately 10 million different TCRB chains contained within their 10^{12} circulating T-cells(70)) it is highly improbable to randomly converge on the same TCRB nucleotide CDR3 sequence, effectively making each CDR3 sequence a unique tag for a T-cell clone. Adaptive Biotechnologies' ImmunoSEQ assay, a multiplex PCR-based method that amplifies rearranged TCR CDR3 sequences and exploits the capacity of highthroughput sequencing technology characterizes tens of thousands of TCRB CDR3 chains simultaneously. Thus, the assay captures the full TCR repertoire including specific individual clones. The ImmunoSEQ assay provides a novel method to identify and track the presence and frequency of common and rare clones, in the context of the total adaptive immune system. Recently, ImmunoSEQ showed profound evolution and diversification of the TCR repertoire of men with mCRPC treated with the immune stimulatory agent ipilimumab (71). Improved clinical outcomes were associated with less T-cell clonotype loss, consistent with the maintenance of high-frequency TCR clonotypes during treatment reported by ImmunoSEQ. These clones may have represented the presence of preexisting high-avidity T cells that may be relevant in the antitumor response.

5. PARTICIPANT SELECTION AND ENROLLMENT PROCEDURES

All patients will be eligible to receive systemic therapy alone at the time of clinical or radiographic disease progression.

5.1 Inclusion Criteria

5.1.1 Patient must have at least one and up to three asypmtomatic metastatic tumor(s) of the bone or soft tissue develop within the past 6-months that are ≤ 5.0 cm or <250 cm^3

5.1.2 Patient must have had their primary tumor treated with surgery and/or radiation.

5.1.3 Patient must have ≤ 3 metastatic bone sites.

5.1.4 Histologic confirmation of malignancy (primary or metastatic tumor).

5.1.5 PSADT <15 months. PSA doubling time (PSADT) will be calculated using as many PSA values that are available from time of relapse (PSA > 0.2). To calculate PSADT, the Memorial Sloan Kettering Cancer Center Prostate Cancer Prediction Tool will be used. It can be found at the following web site: https://www.mskcc.org/nomograms/prostate/psa-doubling-time.

5.1.6 PSA ≥1 but ≤50.

5.1.7 Testosterone ≥ 125 ng/dL.

5.1.8 Patient must be ≥ 18 years of age.

5.1.9 Patient must have a life expectancy ≥ 12 months.

5.1.10 Patient must have an ECOG performance status ≤ 2.

5.1.11 Patient must have normal organ and marrow function as defined as:

Leukocytes	≥2,000/μL
Absolute Neutrophil Count	≥1,000/μL
Platelets	≥50,000/μL

5.1.12 Patient must have the ability to understand and the willingness to sign a written informed consent document.

5.2 Exclusion Criteria

5.2.1 Patient may not have had any prior systemic therapy allowed aside from ADT associated with treatment of their primary prostate cancer or with salvage radiation therapy and for not more than 6 months with the most recent ADT treatment having occurred greater than 6 months prior to enrollment.

5.2.2 Castration-resistant prostate cancer (CRPC).

5.2.3 Spinal cord compression or impending spinal cord compression.

5.2.4 Suspected pulmonary and/or liver metastases (greater ≥10 mm in largest axis).

5.2.5 Patient receiving any other investigational agents.

5.2.6 Patient is participating in a concurrent treatment protocol.

5.2.7 Serum creatinine > 3 times the upper limit of normal.

5.2.8 Total bilirubin > 3 times the upper limit of normal.

5.2.9 Liver Transaminases > 5-times the upper limit of normal.

5.2.10 Unable to lie flat during or tolerate PET/MRI, PET/CT or SBRT.

5.2.11 Liver Transaminases > 5-times the upper limit of normal.

5.2.12 Prior salvage treatment to the primary prostate cancer or pelvis is allowed.

5.2.13 Refusal to sign informed consent.

6. TREATMENT PLAN

6.1 Randomization

Eligibility work-up will include a complete blood count, serum chemistries, PSA, and radiographic studies (CT and/or MRI of the chest, abdomen, and pelvis and bone scan). Once a signed informed consent has been obtained and after confirming patient eligibility, the lead data coordinator will assign a unique Study ID Number.

Treatment must not commence until the patient has received his identification number.

Stratification:
Subjects who meet eligibility criteria and qualify for enrollment will be stratified according to the following:

1) Initial treatment with surgery vs. radiation therapy
2) Prior hormonal therapy vs. no prior hormonal therapy
3) PSADT <6 months vs. 6-14.9 months

Process for Randomization:
The research team will utilize an interactive web response system (IWRS) to obtain the patient's randomization assignment. Randomization will be 1:2 for observation:SBRT arms and be stratified as above. Minimization approach will be applied to ensure balanced assignment to each treatment arm. The **ON STUDY date for protocol entry** will be the day that the study subject is randomized.

6.2 Diagnostic Procedures

A bone scan and CT (and/or MRI of questionalbe sites) must be avilaible or will be obtained within 3-months of randomization for confirmation of oligometastatses and a separate DCFPyL-PET/MRI or -PET/CT scan will be performed for protocol purposes as below in the patients randomized to the SBRT arm. A CT- and/or MRI-simulation scan will be performed for tumor localization and radiation planning using rigid immobilization appropriate for stereotactic treatment.

6.3 Therapeutic Procedures

Upon confirmation of eligibility and enrollment in the study, the following will be confirmed or completed:

1) Demographics review, medical history and clinical exam
2) Review of concurrent medications
3) Vital signs, height and weight
4) PSA, testosterone, CBC, chemistry panel, LDH and coagulation profile
5) CT and/or MRI scan of the involved site(s) (if not previously conducted within 3 month of enrollment).
6) Bone scan (if not previously conducted within 3 month of enrollment).
7) **DCFPyL-PET/MR or -PET/CT Diagnostic Imaging Protocol. <u>For men randomized to the SBRT arm only</u>.** Our collaborating center at the NIH Clinical Center has a PET/MRI available and will proceed with these exams, while at the Johns Hopkins campus will be proceed with PET/CT. The subjects accrued at the NIH will be scanned on a Siemens Biograph mMR PET/MR scanner, a simultaneous PET and MR imaging system. The system is operated by the radiology department of the National Institutes of Health clinical center located on the NIH campus in Bethesda MD. The Johns Hopkins campus has Discovery DRX PET/CT scanner (GE Healthcare) imaging systems.

Patient Preparation.
i. Patient has to be NPO for 4-6 hrs.

ii. When the patient arrivals in the clinic they are explained the procedure by the Nuclear Medicine Staff and all questions are answered prior the start of the procedure.
iii. IV access is obtained and verified that it is patent with a saline flush prior to giving the F-18 DCFPyL.
iv. A dose of 9-10 mCi F-18 DCFPyL is injected through the IV and followed by at least 10 ml of saline to flush the IV line of the remaining dose.
v. Intravenous fluid (5% dextrose + 0.45% normal saline) will be delivered at a low flow rate during the duration of the study (maximum 2 litters).
vi. The patient is asked to void prior to the scan 15-20 minutes before the scan. After the patient is finished voiding, they are positioned on the scanner bed with the head placed first and arms down to their side.
vii. A CT or (MR) localizer scan is taken from the top of the head to mid-thigh to properly frame the subjects skull vertex through mid-thigh in the CT (or MR) scanner's field of view. It is expected to require 7 to 9 bed positions.
viii. At the 1 hour post injection time (50-70 minutes), the PET/CT or PET/MR imaging sequence begins.
ix. The PET system of the CT (or MR) scanner will collect data for each bed position required to scan the subject from the skull vertex to mid-thigh. The scan time for each bed position will be on the order of 7 to 8 minutes depending on the details setup of the MR imaging sequences. The PET images will be reconstructed using a 3D iterative reconstruction algorithm, with 3 iterations and 24 subsets. Detector pair normalization, random scatter and attenuation corrections will be applied during the image reconstruction process. No image post filtering will be applied.
x. Simultaneous with the PET acquisition the following MR imaging sequences will be collected.

a. A T1 weighted gradient echo TR=200ms,TE =2.3ms, slice thickness=5mm, matrix =376x116, FOV 47cm
b. DWI TR=6000ms, TE=79ms,slice thickness=5mm, matrix=128x128, b=0,1000,2000,FOV 47cm
c STIR TR=12000, TE=110,TI=200ms, slice thickness=5mm, matrix =376x116, FOV 47cm

The study PI, co-I's or study coordinator will contact the patient around 7 days (3 - 10 days) after the PET/CT or PET/MRI study to inquire about delayed side effects. If there are suspected problems the patient will be asked to return for a clinic visit for a follow-up clinic visit.

DCFPyL-PET/MRI or -PET/CT images will be evaluated and compared to bone scan. However, additional sites(s) of suspected metastatic disease detected by DCFPyL-PET/MRI or -PET/CT will not be considered for treatment by SBRT/SABR or undergo further required evaluation. The results of the DCFPyL-PET/MRI or –PET/CT will not be made available to the treating physicians until after the trial is completed and these results will not be made available to the patient.

8) SBRT/SABR treatment planning

CT- and/or MRI-simulation will then be performed with fabrication of a radiation therapy immobilization device (such as the Alpha Cradle) which will be custom made for each patient. The treating radiation oncologist will identify the location of the tumor. Gross tumor volume (GTV) delineation will be performed with a diagnostic radiologist on sequential axial computed tomography images. A radiosurgical treatment plan will be developed based on tumor geometry and location. The clinical tumor volume (CTV) will equal the GTV. The dose will be prescribed to the minimal isodose line that completely covers the planning target volume (PTV) PTV (=CTV plus a 5 mm margin). Adjacent normal structures including but not limited to the heart, esophagus, aorta, spinal cord, kidneys, rectum, bowel, liver, and stomach within 5 cm of the CTV will be identified for the purpose of limiting incidental radiation to these structures.

In addition, prior to treatment delivery, a four-dimensional cone beam CT study will be performed on individual patients to assess respiration in these patients and to determine tumor targeting accuracy for those tumors that may be subject to respiratory motion such as those in the bones of the thorax. If tumor motion is greater than 5 mm, PTV will be expanded to account for respiration.

SBRT/SABR will be delivered in 1 to 5 fractions, and the dose and fractionation schedule will depend on the size and location of the lesion and the surrounding normal tissue constraints in accordance with AAPM Task Group 101 recommendations. Typical doses include 16 – 24 Gy in 1 fraction, 48 – 50 Gy in 4 fractions, and 50 – 60 Gy in 5 fractions. For example, isolated osseous lesions will be treated in a single fraction, lesions close to the lung and liver lesions will be treated in 3 to 5 fractions depending on their size (5 fractions for ≥ 3 cm or central tumors in close proximity to the mediastinum), and bone lesions will be treated in 5 fractions if small-bowel constrains fewer doses.

Within three weeks of the initial treatment planning imaging study, SBRT/SABR will be administered using image-guidance. An Alpha Cradle (or equivalent immobilization device) will be used to minimize movement of the chest, spine, and abdomen during treatment. During treatment, real time cone beam CT images of the patient's body site of interest will be obtained. Cone beam CT scan will be obtained immediately prior to treatment and will be repeated until the treatment shift, required to align the CT planning scan and the cone beam CT scan performed on the day of treatment cone beam CT, is within tolerance for the body site.

Patients will be evaluated for adverse events/toxicities during their treatment.

6.3.1 The dose limits for surrounding critical structures are as follows:

Spinal Cord: maximal allowable dose should be = 800 cGy in 1 fraction

Lung: 2/3 of the lung volume should be kept under 500 cGy.

Heart: 50 % of the heart volume should be kept under 1000 cGy.

Esophagus: 50 % of the esophagus volume should be kept under 1000 cGy and no single point dose in the esophagus should exceed 2000 cGy.

Brachial Plexus: maximal allowable point dose = 1000 cGy

Liver: One third of the uninvolved liver or approximately 700 cc <15 Gy.

Kidneys: 75% of volume of each kidney <5 Gy.

Small Bowel: <5% of bowel limited to <20 Gy.

6.4 Follow-Up Procedures

Subsequent to randomization, patients will be followed clinically and radiographically. A detailed medical and physical examination with blood draws for PSA, LDH, AP and testosterone will be performed at month 3 and 6 for all patients. The patients randomized to the SBRT arm will also have blood draws for CTCs/ctDNA and immune cell profiling (ImmunoSEQ) correlative studies. In clinic visits are not required otherwise, unless indicated by laboratory tests. Bone scan and CT scan of metastatic site(s) will be at 6 months for all patients, unless indicated more frequently by clinical or laboratory findings. The patients randomized to the SBRT arm will also have DCFPyL-PET/MRI or –PET/CT correlative studies. The results of the DCFPyL-PET/MRI or –PET/CT will not be made available to the treating physicians until after the trial is completed and these results will not be made available to the patient.

CT scans at 6 months will be used to determine radiographic response based on RECIST 1.1 criteria for soft tissue lesions.

Correlative science blood draws will be performed at baseline, day 90 (3-months) and day 180 (6-months) for patients randomized to the SBRT arm. Details for these blood draws are contained within the APPENDIX III-V.

Briefly:

Cell Search CTC^{TM}: Will be ordered per Quest labs and is a 7.5-ml whole blood draw.

CAPP–Seq: 30-ml whole blood in purple top (EDTA) tubes (see APPENDIX III for details).

EPIC HD-CTC: 4-10 ml Streck Cell-Free DNA BCT tubes (see APPENDIX IV for details).

ImmunoSEQ: 10-ml whole blood in purple top (EDTA) tubes (see APPENDIX V for details).

6.5 Duration of Therapy

Within three weeks of the initial treatment planning imaging study, SBRT will be administered in a 1-5 fractions to each treated site.

6.6 Duration of Follow-Up

A trial with a similar patient population took 2.5 years to screen 77 patients (72). Thus we anticipate this study will last 2-2.5 years.

6.7 Criteria for Patient Removal

Diagnosis of >3 bone metastases on entry imaging studies before SBRT/SABR. PSA >50 ng/ml before SBRT. Progression as defined in section 9.6.2.

6.8 Alternatives

The study has been designed to minimize potential risks to participants. First, this population of asypmtomatic oligometastatic men is heavily pre-selected based on institutional data indicating that similar men without metastases will progress in 6-months based on rising serum PSAs (72-74). Secondly, the level of clinical-radiographic interrogation will allow the control arm to be safely observed so that standard of care treatments can be initiated within a typical interval between clinic visits (72-74). Lastly, the SBRT dose has been shown to be safe in previous SBRT trials. Risks to confidentiality will be minimized by having access to study records avaialble only to the investigators with the exception of the standard clinical records (lab values, dictations, operative notes, etc).

Standard therapies for metastatic prostate disease include radiotherapy, ADT or observation. Such treatment may or may not be applicable for patients enrolled in this study. Regardless, patients will be expected to forgo standard treatment until there is evidence of clinical, biochemical (PSA >50 ng/ml) or radiographic disease progression.

6.9 Costs

Patients and/or their insurance companies will be responsible for the cost of all procedures and treatments under this protocol.

6.10 Compensation

Patients and/or their insurance companies will be responsible for the cost of all procedures and treatments under this protocol.

6.11 Withdrawal from Study

The reasons for withdrawal from the study include:

- Subject withdraws consent for follow-up.
- Subject is lost to follow-up.
- Study is terminated for any reason.

6.12 Cross-over

The men on the control arm are allowed to cross over and be treated with SBRT following documented progression as defined in section 9.6.2.

7. INVESTIGATIONAL AGENT/DEVICE/PROCEDURE INFORMATION

7.1 Investigational Agent - DCFPyL

(This information is loaded into the IND application section of eIRB. Additional radiopharmaceutical information not listed below is downloaded on the FDA IND

application in the supporting documents section of eIRB and available there for review.)

8. STUDY CALENDAR

	Pre-Study	Randomization (+/- 7 days)	Day 1 (+/- 7 days)	Day 1-30 SBRT/SABR	Day 90 (+/- 7 days)	Day 180 (+/- 7 days)
**Informed Consent**		X				
**Demographics**	X					
**Medical History & Review of Medications**	X			X[d,g]	X	X
**Physical Exam**	X			X[d,g]	X	X
**Vital Signs**	X					
**Height**	X					
**Weight**	X					
**Performance Status**	X				X	X
**CBC w/ Diff[a]**	X		X		X	X
**Serum Chemistry[b]**	X		X		X	X
**Testosterone**	X		X		X	X
**CellSearch CTC**			X[d]		X[d]	X[d]
**Correlative Blood Draws**			X[d,e]		X[d,e]	X[d,f]
**Bone scan & CT of Involved Site[c]**	X					X[h]
**DCFPyL-PET/CT or**			X[d]			X[d]

**PET/MR**						
**PSA**	X		X		X	X
**AE Evaluation**			X	X[d,g]	X	X
**QoL - BPI**			X	X[d,g]	X	X

a. Including platelets.
b. Albumin, Alkaline Phosphatase (AP), Total Bilirubin, Bicarbonate, BUN, Calcium, Chloride, Creatinine, Glucose, LDH, Phosphorus, Potassium, Total Protein, SGOT [AST], SPGT [ALT], Sodium.
c. Enrollment bone scan and CT-imaging studies need to be within 3-months of randomization.
d. Only for the SBRT arm.
e. See APPENDIX III-V for details regarding CAPP-Seq, EPIC HD-CTC & ImmunoSEQ.
f. Only for CAPP-Seq & EPIC HD-CTC.
g. Can be during SBRT on-treatment visit.
h. Tumor measurements are to be calculated on CT scans at 6 months.

9. MEASUREMENT OF EFFECT

9.1 Antitumor Effect – Metastatic Tumors

For the purposes of this study, patients should be re-evaluated for radiographic response 6 months after randomization. Trial radiologists evaluating for treatment responses will be blinded to the treatment group and treatment specifics (lesions treated with SBRT).

Response and progression will be evaluated in three ways:

1) Using Response Evaluation Criteria in Solid Tumors (RECIST 1.1) Committee definition. Changes in only the largest diameter (unidimensional measurement) of the tumor lesions are used in the RECIST criteria.
2) Lesions by bone scan will be evaluated as positive, negative or no change. MRI of the bone lesion can be used to clarify equivocal lesions.
3) Serial PSA changes.

9.2 Definitions

Evaluable Population: will consist of all patients who have received SBRT.

Safety Population: Will consist of all subjects who were enrolled and have undergone at least one fraction of SBRT. This will be used to assess the clinical safety and tolerability of the study.

Evaluable for Objective Response: Only those patients who have measurable disease present at baseline, have completed all fractions of SBRT, and have had their disease re-evaluated will be considered evaluable for response. These patients will have their response classified according to the definitions stated below.

9.3 Disease Parameters

Measurable Disease: Measurable lesions are defined as those that can be accurately measured in at least one dimension (longest diameter to be recorded) as ≥10 mm with diagnostic techniques (CT, or MRI). All tumor measurements must be recorded in millimeters (or decimal fractions of centimeters). Serial PSA measurements will also be analyzed.

Non-Measurable Disease: All other lesions (or sites of disease), including small lesions (longest diameter <10 mm with diagnostic techniques), are considered non-measurable disease. Bone lesions, leptomeningeal disease, ascites, pleural/pericardial effusions, lymphangitis, abdominal masses (not followed by CT or MRI), and cystic lesions are all non-measurable.

Target Lesions: Target lesions in this study will be considered oligometastatic sites up to a maximum of 3 lesions per patient. They should be recorded and measured at baseline. Target lesions should be equal to or larger than 10 mm in

the smallest cross-sectional diameter on CT or MRI and/or any lesion that shows increase uptake on bone scans. A sum of the longest diameter (LD) for all target lesions will be calculated and reported as the baseline sum LD. The baseline sum LD will be used as reference by which to characterize the objective tumor response. For bone scans we will also use a simple evaluation system composed of positive, negative or no change to lesions.

Non-Target Lesions: N/A

9.4 Methods for Evaluation of Measurable Disease

All measurements should be taken and recorded in metric notation using a ruler or calipers. All baseline evaluations should be performed as closely as possible to the beginning of treatment and never more than 4 weeks before the beginning of the treatment.

The same method of assessment and the same technique should be used to characterize each identified and reported lesion at baseline and during follow-up. Imaging-based evaluation is preferred to evaluation by clinical examination when both methods have been used to assess the antitumor effect of a treatment.

Conventional CT, PET/CT, and MRI. These techniques should be performed with cuts of 10 mm or less in slice thickness contiguously. Spiral CT should be performed using a 5 mm contiguous reconstruction algorithm. This applies to tumors of the chest, abdomen, and pelvis. Head and neck tumors and those of extremities usually require specific protocols.

Secondary objectives for comparison of DCFPyL-PET/MRI or –PET/CT to bone scan at baseline and 1-year following SBRT/SABR. We will estimate 1) the proportion of metastatic lesions found on bone scan that are DCFPyL-PET/MRI or –PET/CT positive and 2) the proportion of DCFPyL-PET/MRI or –PET/CT positive sites that are positive for metastatic disease by bone scan for prostate cancer. The results of the DCFPyL-PET/MRI or –PET/CT will not be made available to the treating physicians until after the trial is completed and these results will not be made available to the patient.

9.5 Response Criteria

9.5.1 Evaluation of Target Lesions/PSA Response

Complete Response (CR): Disappearance of all target lesions and PSA < pre SBRT PSA

Partial Response (PR): At least a 30% decrease in the sum of the longest diameter (LD) of target lesions, taking as reference the baseline sum LD. Or a third of the lesions are

negative or no change by bone scan and PSA ≤ pre-SBRT PSA.

Progressive Disease (PD): At least a 20% increase in the sum of the LD of target lesions, taking as reference the smallest sum LD recorded since the treatment started or the appearance of ≥1 new lesion(s). Or ≥1 new lesion(s) appear by bone scan. Or PSA ≥25% increase in PSA from nadir or > 50 ng/ml.

Stable Disease (SD): Neither sufficient shrinkage to qualify for PR nor sufficient increase to qualify for PD, taking as reference the smallest sum LD since the treatment started. Or PSA ≥ pre-SBRT PSA, but not ≥25% increase in PSA from nadir and <50 ng/ml.

9.5.2 Evaluation of Non-Target Lesions

N/A

9.5.3 Evaluation of Best Overall Response

The best overall response is the best response recorded from the start of the treatment until disease progression/recurrence (taking as reference for progressive disease the smallest measurements recorded since the treatment started). Best overall response will be based on the overall response of the target lesions.

9.6 Imaging Reference Standard, as Applicable (i.e., "Gold standard")

Reference standard will be determined by a truth panel consisting of a nuclear medicine physician, radiologist, and GU oncologist. This panel will determine whether individual sites of suspected metastatic disease on initial baseline CIM (bone scan and chest/abdomen/pelvis CT) and DCFPyL-PET/MRI or -PET/CT findings are truly positive based on following data:

- prior available imaging
- available follow-up imaging obtained as part of clinical care and/or investigational protocol and treatment history for up to 12 months from the time of DCFPyL-PET/MRI or –PET/CT scan.
- The results of the DCFPyL-PET/MRI or –PET/CT will not be made available to the treating physicians until after the trial is completed and these results will not be made available to the patient.

9.6.1 Duration of Response

Response will be defined as evidence of CR, PR, or stable disease. The duration of response will be measured from the start of treatment until the criteria for progression are met.

Duration of CR or PR: The duration of CR or PR will be recorded from the time measurement criteria are met for CR or PR (whichever is first recorded) until the first date that current or progressive disease is objectively documented (taking as reference for progressive disease the smallest measurements recorded since the treatment started).

Duration of Stable Disease: Stable disease is measured from the start of the treatment until the criteria for progression are met, taking as reference the smallest measurements recorded since the treatment started.

9.6.2 Clinical Response Parameters

Progression is a composite endpoint defined from the Prostate Cancer Working Group 2 (PCWG2) criteria for metastatic castrate resistant prostate cancer (mCRPC) (75) and our previous trials in a population of men with biochemical failure without metastases (72-74). Progression will be defined as either: 1) a ≥25% increase in PSA from nadir (and by ≥2 ng/mL), requiring confirmation ≥4 weeks later (PCWG2 criteria); and/or, 2) clinical/radiographic-progression defined as symptomatic progression (worsening disease-related symptoms or new cancer-related complications), or radiologic progression (on CT scan: ≥20% enlargement in sum diameter of soft-tissue target lesions [RECIST 1.1 criteria]; on bone scan: ≥1 new bone lesions), initiation of ADT or death due to any cause, whichever occurs first. Death is considered as an event here.

Progression Free Survival (PFS) is defined as the time from starting treatment to the time of progression as defined above. Subjects who do not progress will be censored at the time of the last contact.

ADT Free Survival (ADT-FS) is defined as the time from starting treatment to the time of initiation of palliative ADT. ADT will typically be initiated on tumor progression and/or development of new metastases. Subjects who do not start ADT will be censored at the time of the last contact.

Time to Progression (TTP) is defined as the time from starting treatment to the time of first documented tumor progression or new lesions by CT and/or bone scan or initiation of ADT. Subjects who do not progress will be censored at the time of the last contact. In addition, death from any cause will also be censored.

Overall Survival (OS) is defined as the time from starting treatment until death due to any cause. For subjects who do not die, time to death will be censored at the time of last contact.

Locoregional Control (LRC) is defined as the time from starting treatment until local and/or regional relapse is documented

9.6.3 Response Review

All responses will be reviewed by the study co-investigator radiologists.

10. ADVERSE EVENT REPORTING REQUIREMENTS

10.1 General

Adverse event collection and reporting is a routine part of every clinical trial. This study will use the descriptions and grading scales found in the NCI Common Terminology Criteria for Adverse Events version 4.0 (CTCAE v 4.0) that is available at http://ctep.cancer.gov/reporting//ctc.html.

Information on all adverse events, whether reported by the participant, directly observed, or detected by physical examination, laboratory test or other means, will be collected, recorded, followed and reported as described in the following sections.

Participants should be instructed to report any serious post-study event(s) that might reasonably be related to participation in this study. The investigator should notify the IRB and any other applicable regulatory agency of any unanticipated death or adverse event occurring after a participant has discontinued or terminated study participation that may reasonably be related to the study.

10.2 Definitions

10.2.1 Adverse Event (AE)

An adverse event is any undesirable sign, symptom or medical condition or experience that develops or worsens in severity after starting the first dose of study treatment or any procedure specified in the protocol, even if the event is not considered to be related to the study.

Abnormal laboratory values or diagnostic test results constitute adverse events only if they induce clinical signs or symptoms or require treatment or further diagnostic tests.

10.2.2 Serious adverse event (SAE)

A serious adverse event is an undesirable sign, symptom, or medical condition which:

- is fatal or life-threatening;
- requires or prolongs inpatient hospitalization;
- results in persistent or significant disability/incapacity;
- constitutes a congenital anomaly or birth defect; or
- jeopardizes the participant and requires medical or surgical intervention to prevent one of the outcomes listed above.

Events **not** considered to be serious adverse events are hospitalizations for:

- routine treatment or monitoring of the studied indication, not associated with any deterioration in condition, or for elective procedures

- elective or pre-planned treatment for a pre-existing condition that did not worsen
- emergency outpatient treatment for an event not fulfilling the serious criteria outlined above and not resulting in inpatient admission
- respite care

10.2.3 Expectedness

- Expected: Expected adverse events are those that have been previously identified as resulting from administration of the agent. For the purposes of this study, an adverse event is considered expected when it appears in the current adverse event list, the Investigator's Brochure, the package insert or is included in the informed consent document as a potential risk.
- Unexpected: An adverse event is considered unexpected when it varies in nature, intensity or frequency from information provided in the current adverse event list, the Investigator's Brochure, the package insert or when it is not included in the informed consent document as a potential risk

10.2.4 Attribution

Attribution is the relationship between an adverse event or serious adverse event and the study treatment. Attribution will be assigned as follows:

- Definite – The AE is clearly related to the study treatment.
- Probable – The AE is likely related to the study treatment.
- Possible – The AE may be related to the study treatment.
- Unlikely - The AE is doubtfully related to the study treatment.
- Unrelated - The AE is clearly NOT related to the study treatment.

10.3 Potential Adverse Events

Signs and symptoms of disease progression are not considered AEs. The development of a new cancer should be regarded as an AE. New cancers are those that are not the primary reason for administration of study treatment and have been identified after inclusion of the patient into the clinical study.

Because patients are receiving standard treatments, which are not part of this study, their treating physician will be counseling them on the risk of their treatments, including the risk of surgery, radiation therapy, and/or chemotherapy, whichever is appropriate for the type and the stage of their cancer. The procedures related to the study are phlebotomy, PET imaging, SBRT, and DCFPyL administration.

Phlebotomy can cause pain, bleeding, and rare needle site infection. PET imaging results in low dose radiation exposure (see Investigator's Brochure for details of dosimetry), which has an extremely small risk of causing a secondary cancer.

10.4 Stereotactic Body Radiation Treatment (SBRT) or Stereotactic Ablative Radiation Treatment (SABR)

It is difficult at this time to predict with confidence the complication rate from the proposed SBRT/SABR; however, it is reasonable to extrapolate from the current experience with SBRT/SABR to the lung, prostate, spine, liver and pancreas. One significant toxicity is radiation pneumonitis, which can be manifested as fever, increased excertional dypsnea, pleuritic chest pain, and peritumoral infiltrate on chest imaging. It generally occurs between 1 to 3 months of completion of radiotherapy. The risk of grade 2-4 radiation pneumonitis is aproximately 10-15% in patients treated with standard fractionated large field radiotherapy and higher in patients treated with combined chemoradiotherapy. It is highly dependent on the volume of the lung treated to high dose and the mean lung dose. At this point, the incidence of RT pneumonitis from stereotactic radiosurgery for small pulmonary tumors is unknown. However, if the treated tumor volume is kept ≤ 65 cc, the risk should be < 10-15% with the proposed dose level.

Other toxicities commonly associated with such treatment includes dysphagia, odynophagia, nausea, vomiting, anorexia, and weight loss. Some of these symptoms can also be due to tumor progression. Clinical and radiographic assessments will be performed as indicated to identify all adverse effects, ascertain their etiology, and provide the most appropriate palliative measures. Complications orther than radiation pneumonitis, if any, will be graded according to the Common Toxicity Criteria, National Cancer Institute, version 4.0.

10.5 DCFPyL

10.5.1 [^{18}F]DCFPyL is eluted with 1 mL of ethanol followed by 10 mL of 0.9% sodium chloride, via a sterilizing 0.22μ filter into a sterile vial containing 4 mL 0.9% sodium chloride. Test radiopharmaceutical will be administered intravenously via slow I.V. push. The maximum mass dose of the ligand corresponding to the 10 mCi dose of [^{18}F]DCFPyL will be less than 4.02μg per administration, although the actual dose will be significantly less than depending on the specific activity achieved during each radiosynthesis run. The lowest limit for specific-activity (SA) we will use at the time of injection is 1000 mCi/μmole, although the validation and phase I study radiosynthesis SA was significantly higher than 1000 mCi/μmole.

<u>Preclinical Toxicology</u>
A toxicity report “Single Dose Toxicity Study of ^{19}F- DCFBC (NSC-743104) in Rats” was prepared by Bridge GPS, Inc. (Study No. 1535-07015). The following summary is taken from that report.

This study was designed to determine target organ toxicity of 19F- DCFBC (NSC-743104) and its reversibility in rats treated with a single intravenous dose.

Sixty Fischer 344 rats (30/sex) were randomly assigned to one of three dose groups (10/sex/group) based on body weight and physical examination. Five rats/sex/group were designated to the main phase of the study, and five rats/sex/group were designated to the recovery phase of the study. All animals were dosed once on Study Day (SC) 1 via intravenous injection (tail vein) with either the control article (5% dextrose) or 19F- DCFBC (NSC-743104) at nominal dose levels of 0.1 or 0.5 mg/kg. Terminal sacrifice necropsies were performed on SD 4; recovery sacrifice necropsies were performed on SD 15. Parameters evaluated during the study included mortality, clinical and cage side observations, body weights, body weight changes, clinical pathology parameters (clinical chemistry and hematology), gross pathology and histopathology.

Mortality, clinical and cage-side observation, body weight and body weight changes, clinical pathology, gross pathology and histopathology were unaffected by treatment.

Based on the results of this study, a single intravenous injection of 19F-DCFBC (NSC-743104) at doses up to 0.5 mg/kg to male and female Fischer 344 rats was well tolerated.

Table 1: Tissue distribution of [^{18}F] DCFBC (%ID/gm)

	5 min n=5	15 min n=4	30 min n=4	60 min n=4	120 min n=3
Blood	11.34	4.07	2.26	1.80	0.36
heart	4.38	2.01	1.24	0.77	0.27
lung	7.03	3.21	1.78	1.10	0.36
liver	5.99	4.07	4.18	5.12	2.11
stomach	2.87	1.35	0.81	0.46	1.08
pancreas	2.17	1.04	0.99	0.54	0.18
spleen	8.76	4.27	1.94	1.58	0.44
fat	1.75	1.56	0.67	1.05	0.26
kidney	63.40	62.94	51.29	41.55	13.08
bone	2.88	1.61	1.72	1.73	2.46
muscle	2.21	1.00	0.45	0.57	0.24
small intestine	4.29	2.31	1.20	0.66	0.15
large intestine	2.82	1.47	0.78	0.58	0.27
bladder	55.41	15.94	15.18	14.49	2.57
PC-3 PIP	8.17	6.03	6.16	8.16	4.69
PC-3 flu	3.47	1.72	0.97	0.77	0.18
cortex	0.49	0.25	0.13	0.10	0.06

cerebellum	0.58	0.26	0.14	0.10	0.04
PIP:flu	2.36	3.51	6.35	10.57	26.65

Similar toxicity was observed for our second generation compound DCFPyL and was awarded a physician sponsored FDA exploratory IND based in this acceptable pre-clinical data (IND#121,064) for ^{18}F-DCFPyL as a PET radiopharmaceutical, which is held by the co-I, Dr. Martin Pomper.

Initial Phase I study Adverse Events

An initial phase I study of the biodistribution and dosimetry of DCFBC was performed in five men with metastatic prostate cancer from 10/2010 through 12/2010. No serious adverse events (SAE) were observed. One patient with history of hypertension experienced grade III hypertension after administration of DCFBC with no clinical symptoms. Twenty-four hours later his blood pressure returned to baseline grade 2 hypertension and at 7 days post DCFBC administration, his blood pressure normalized to baseline levels. The attribution of this hypertension adverse event (AE) was listed as unlikely (the adverse event is doubtfully related to the investigational imaging tracer) given this patient had been previously controlled with a calcium-channel blocker medication but this was discontinued about two months prior to DCFBC administration and his blood pressure was more likely attributed to stress during the PET/CT scan and overall labile blood pressure of his calcium-channel blocker. No other adverse events noted in all five patients over a 28 day follow-up post study radiopharmaceutical administration.

Human Dosimetry

In our biodistribution and dosimetry study, five patients with radiologic evidence of metastatic prostate cancer were studied after intravenous administration of 370 MBq (10 mCi) of DCFBC. Serial PET imaging was performed out to 2 hour after administration. Time-activity curves were generated for selected normal tissues and metastatic foci. Radiation dose estimates were calculated using OLINDA/EXM 1.1. Most vascular organs demonstrated a slow decrease in activity concentration over time consistent with clearance from blood pool, with primarily urinary radiotracer excretion. The organ with the highest mean absorbed dose (µGy/MBq) is the urinary bladder wall (32.4), followed by the stomach wall (30.2), heart wall (29.2), and kidneys (28.4). The remaining GI tract organs (small intestines, SI; upper large intestines, ULI; lower large intestines, LLI), liver and lungs receive lower absorbed doses. The mean effective dose was 19.9 ± 1.34 µSv/MBq. Dose estimates for ^{18}F- DCFBC are comparable to those of other PET radiopharmaceuticals such as ^{18}F-fluorodeoxyglucose (^{18}F-FDG). (Note: This study has been accepted for publication in the Journal of Nuclear Medicine in the fall of 2012 (35) and reported this dosimetry data to the FDA as part of a protocol revision for Protocol/IND 108,943 on 8/30/2011.

Table 2: Human Average Organ Absorbed Dose (mGy/MBq) and estimated effective dose (mSv/MBq)

	Average	Std Dev	COV
Adrenals	1.85E-02	2.83E-03	15.32%
Brain	4.21E-03	2.83E-04	6.73%
Breasts	8.51E-03	3.22E-04	3.78%
GB Wall	1.79E-02	1.95E-03	10.90%
LLI Wall	2.47E-02	3.69E-03	14.92%
SI Wall	2.36E-02	1.72E-03	7.31%
Stomach Wall	3.02E-02	3.24E-03	10.72%
ULI Wall	2.34E-02	2.20E-03	9.39%
Heart Wall	2.92E-02	3.24E-03	11.12%
Kidneys	2.84E-02	3.81E-03	13.45%
Liver	2.46E-02	4.16E-03	16.88%
Lungs	2.45E-02	2.99E-03	12.22%
Muscle	9.69E-03	3.97E-04	4.10%
Ovaries	1.32E-02	5.26E-04	3.99%
Pancreas	1.92E-02	2.15E-03	11.19%
Red Marrow	1.70E-02	9.81E-04	5.79%
Osteogenic Cells	1.82E-02	8.92E-04	4.90%
Skin	7.30E-03	3.50E-04	4.79%
Spleen	1.72E-02	1.05E-03	6.08%
Testes	1.54E-02	4.19E-03	27.23%
Thymus	1.10E-02	4.53E-04	4.12%
Thyroid	1.17E-02	6.87E-04	5.88%
Bladder Wall	3.24E-02	7.24E-03	22.35%
Uterus	1.34E-02	2.95E-04	2.20%
Total Body	1.09E-02	4.28E-04	3.91%
ED	1.99E-02	1.34E-03	6.73%

10.6 Reporting Procedures

10.6.1 General

Adverse events will be recorded at each visit. If an adverse event requiring medical attention occurs between visits, this will be recorded as well. The variables to be recorded for each adverse event include, but are not limited to, onset, resolution, intensity, action taken, outcome, causality rating and

whether it constitutes an SAE or not. The intensity of the adverse event should be captured using CTCAE criteria, version 4.0, when possible.

Pregnancy should be excluded before enrollment. Pregnancy in itself is not reported as an AE unless there is a suspicion that an investigational product may have interfered with the effectiveness of a contraceptive medication.

All Serious Adverse Events (SAEs) will be reported via the AdEERS (Adverse Event Expedited Reporting System) application accessed via the CTEP web site (https://webapps.ctep.nci.nih.gov/openapps/plsql/gadeers_main$.startup).

All adverse events will be captured on the appropriate study-specific case report forms (CRFs).

10.6.2 Expedited FDA Reporting Requirements for Unexpected and Related Serious Adverse Events (per 21CFR312.32)

<u>7 Calendar-Day Telephone or Fax IND Safety Report</u>
The Sponsor-Investigator is required to notify the FDA of any fatal or life-threatening adverse event that is unexpected and assessed by the Sponsor-Investigator to be possibly related to the use of DCFPyL within 7 calendar-days of first learning of the event. An unexpected adverse event deemed possibly related to the use of an investigational study drug is defined as any adverse drug experience of which the specificity or severity is not consistent with the current investigator brochure, the general investigational plan, or elsewhere in the current application, as amended.

Such reports are to be telephoned or faxed to the FDA within 7 calendar-days of first learning of the event. Each telephone call or fax transmission should be directed to the FDA new drug review division in the Center for Drug Evaluation and Research or in the product review division for the Center for Biologics Evaluation and Research, whichever department is responsible for the review of the IND.

<u>15 Calendar-Day Written IND Safety Report</u>
The Sponsor-Investigator (Dr. Martin Pomper) is required to notify the FDA, and all participating investigators (as applicable), in a written IND Safety Report, of any serious, unexpected adverse event considered by the Sponsor-Investigator to be possibly related to the use of DCFPyL within 15 calendar days of first learning of the event. If applicable, the Sponsor-Investigator must also notify the FDA, and all participating investigators (as applicable), of any finding from tests in laboratory animals that suggests a significant risk for human subjects including reports of mutagenicity, teratogenicity, or carcinogenicity within 15 calendar-days of first learning of the event.

A serious, unexpected adverse event deemed possibly related to the use of an investigational study drug is any adverse drug experience of which the specificity or severity is not consistent with the current investigator brochure, the general investigational plan, or elsewhere in the current application, as amended, and results in any of the following outcomes:

- Death (report first as a 7-day telephone/fax report);
- life-threatening adverse drug experience (report first as a 7-day telephone/fax report);
- inpatient hospitalization or prolongation of existing hospitalization; a persistent or significant disability/incapacity, or a congenital anomaly/birth defect; or is an important medical event that may not result in death, be life-threatening, or require hospitalization but is considered a serious adverse drug experience when, based upon appropriate medical judgment, it may jeopardize the patient or subject and may require medical or surgical intervention to prevent one of the outcomes listed in this definition.

All written IND Safety Reports should include an Analysis of Similar Events in accordance with 21CFR312.32. All safety reports previously filed with the IND concerning similar events should be analyzed. The new report should contain comments on the significance of the new event in light of the previous, similar reports and be submitted to the FDA, Abbott Laboratories, and all participating investigators (as applicable), within 15 calendar-days of first learning of the event. The FDA prefers these reports be documented on a MedWatch 3500A Form, but alternative formats are acceptable (e.g., summary letter). This form is available at http://www.fda.gov/medwatch/report/hcp.htm.

<u>Follow-up Reports</u>

All follow-up information concerning IND Safety Reports should be submitted to the FDA as soon as possible.

10.6.3 Institutional Review Board

All adverse events and serious adverse events will be reported to the IRB per current institutional standards. If an adverse event requires modification of the informed consent, these modifications will be provided to the IRB with the report of the adverse event. If an adverse event requires modification to the study protocol, these modifications will be provided to the IRB as soon as is possible.

11. DATA AND SAFETY MONITORING PLAN

This is a DSMP Level I study under the SKCCC Monitoring Plan (see Appendix VI). A Level I study requires both internal and external data monitoring. The Principal Investigator is responsible for internal monitoring for both safety and data quality. External data

monitoring will be performed by the SKCCC at Johns Hopkins Clinical Research Office Quality Assurance Program (CRO QA).

Data and safety monitoring oversight will be conducted by the SKCCC at Johns Hopkins Safety Monitoring Committee. Per the SKCCC at Johns Hopkins Safety Monitoring plan, the CRO AQ will forward summaries of all monitoring reports to the Safety Monitoring Committee for review. All reportable anticipated and unanticipated protocol events/problems and amendments that are submitted to the IRB will also be reviewed by the Safety Monitoring Committee Chair (or designee) and QA manager.

12. REGULATORY CONSIDERATIONS

12.1 Protocol Review and Amendments

Information regarding study conduct and progress will be reported to the Institutional Review Board (IRB) per the current institutional standards.

Any changes to the protocol will be made in the form of an amendment and must be approved by the IRB prior to implementation.

12.2 Informed Consent

The investigator (or his/her designee) will explain to each subject the nature of the study, its purpose, the procedures involved, the expected duration, the potential risks and benefits involved and any discomfort it may entail. Each subject will be informed that participation in the study is voluntary, that she may withdraw from the study at any time, and that withdrawal of consent will not affect her subsequent medical treatment or relationship with the treating physician(s) or institution. The informed consent will be given by means of a standard written statement, written in non-technical language, which will be IRB approved. The subject should read and consider the statement before signing and dating it, and will be given a copy of the document. No subject will enter the study or have study-specific procedures done before his/her informed consent has been obtained.

In accordance with the Health Information Portability and Accountability Act (HIPAA), the written informed consent document (or a separate document to be given in conjunction with the consent document) will include a subject authorization to release medical information to the study sponsor and supporting agencies and/or allow these bodies, a regulatory authority, or Institutional Review Board access to subjects’ medical information that includes all hospital records relevant to the study, including subjects’ medical history.

12.3 Ethics and GCP

This study will be carried out in compliance with the protocol and Good Clinical Practice, as described in:

1. ICH Harmonized Tripartite Guidelines for Good Clinical Practice 1996.
2. US 21 Code of Federal Regulations dealing with clinical studies (including parts 50 and 56 concerning informed consent and IRB regulations).
3. Declaration of Helsinki, concerning medical research in humans (Recommendations Guiding Physicians in Biomedical Research Involving Human Subjects, Helsinki 1964, amended Tokyo 1975, Venice 1983, Hong Kong 1989, Somerset West 1996).

The investigator agrees to adhere to the instructions and procedures described in it and thereby to adhere to the principles of Good Clinical Practice that it conforms to.

13. STATISTICAL CONSIDERATIONS

13.1 Endpoints

13.1.1 Primary Objective

To determine the proportion of patients with oligometastatic hormone sensitive prostate cancer who have progressed after 6 months from randomization to observation *versus* stereotactic body radiation therapy (SBRT).

13.1.2 Secondary Objectives

- To describe the toxicity of SBRT/SABR delivered for the population enrolled using grading with CTCAE v. 4.0
- To determine local control at 6-months after SBRT/SABR in patients with oligometastatic disease.
- To assess progression free survival (PFS) of this patient population after randomization defined as the time interval between the day of randomization and progression.
- To assess ADT-free survival (ADT-FS) of this patient population after randomization defined as the time interval between the day of randomization and the initiation of ADT. ADT will typically be initiated on progression and/or development of new metastases.
- To assess quality of life following of the observation *versus* SBRT/SABR arms. Brief Pain Inventory form which will be filled out by the patient at the treatment response intervals outlined above.
- Secondary objectives for comparison of DCFPyL-PET/MRI or -PET/CT to bone scan at baseline and 1-year following SBRT/SABR. We will estimate 1) the proportion of metastatic lesions found on bone scan that are DCFPyL-PET/MRI or –PET/CT positive and 2) the proportion of DCFPyL-PET/MRI or PET/CT positive sites that are positive for metastatic disease by bone scan for prostate cancer. Each of these sites of metastatic disease will also be further characterized according to specific

subcategories based on features of the lesion as follows for each modality and their specific features:

- Bone scan
  - Axial skeleton(spine, pelvis, sacrum, sternum, skull, ribs)
  - Appendicular skeleton (arms, legs)
  - Stable versus new or progressive metastatic site
- CT scan
  - Axial versus appendicular skeleton for bone lesions
  - Size of soft tissue lesions (greatest transaxial and perpendicular diameters)
  - Location of soft tissue lesion
- MRI scan
  - Axial versus appendicular skeleton for bone lesions
  - Size of soft tissue lesions (greatest transaxial and perpendicular diameters)
  - Location of soft tissue lesion
- PET
  - Bone versus lymph node versus organ involvement
  - PET-based SUV parameters (SUVpeak, SUVmax)

13.2 Sample Size/Accrual Rate

The primary endpoint will be rate of progression at 6-months. Historical data on this patient population indicate that >80% would show progression as defined above within a 6 month period without treatment, and thus this is the progression rate we expect in subjects in the control/observation arm (72-74). We hypothesize that SBRT will be able to reduce the progression at 6-months by 50% (76). A sample size using a 1:2 randomization scheme of 18 patients in the control group and 36 in the SBRT arm will provide 85% power to detect a decrease relapse rate from 80% to 40% with a type I error = 0.05 using one-sided Fisher's exact test. Thus, we will have a total of 54 patients.

There will be no interim analysis for futility, since the progression endpoint will not have been reached by a meaningful number of patients before full accrual.

13.3 Early Stopping Guidelines:

This study will monitor site-specific grade 4/5 toxicity in the SBRT arm. If it becomes evident that the proportion of grade 4/5 toxicity at specific sites convincingly exceeds 20%, the study will be halted for a safety consultation. Specifically, we will apply a Bayesian toxicity monitoring rule that suspends the enrollment if the posterior probability of toxicity being larger than 20% threshold is 75% or higher. The monitoring rule uses Beta (0.5, 5.5) as prior distribution. This means that our prior guess of the proportion of toxicity is 8.3%, and there is 90% chance that this proportion is 0.04%-30.6%. The monitoring will start from the first patient, and the decision rule for safety stopping is as follows:

Stop if:

# grade 4/5 toxicity >=	3	4	5	6	7	8	9	10
Out of # patients	3 - 5	6 - 10	11 - 14	15 - 18	19 - 23	24 - 27	28 - 32	33 - 36

The operating characteristics of the stopping rule are shown below and are based on 5000 simulations:

True AE rate	% simulated trials declaring unsafe	Average sample size
0.10	2.6	35.3
0.2	31.5	29.6
0.25	56.4	24.6
0.3	78.4	19.2
0.35	91.9	14.7

13.4 Analysis of Primary Objective

This is a (1:2) randomized, Phase II trial of observation *versus* SBRT in oligometastatic hormone sensitive prostate cancer patients. Minimization approach (77) will be applied to ensure balanced assignment to each treatment arm by stratification factors: 1) Initial treatment with surgery vs. radiation therapy; 2) Prior hormonal therapy vs. no prior hormonal therapy; and 3) PSADT <6 mos vs. 6-14.9 mos. Baseline PSA level is defined as that measured Day 1 following randomization. Progression is defined as per section 9.6.2.

The primary outcome of interest is the proportion of patients who have progressed after 6 months from randomization. For each arm, we will calculate the proportion of patients who have progressed and exact 95% confidence intervals. If a patient has withdrawn from the study before 6 months, they will be considered to have progressed when calculating the proportion of individuals who have progressed. We will compare the proportion of patients who have progressed in the observation and SBRT arms using Fisher's exact test. The analysis population includes all randomized subjects based on intend-to-treat principle.

13.5 Analysis of Secondary Objectives

- For safety analysis, adverse events will be summarized by type and grade.
- Hazard rate estimates and 95% confidence intervals as well as Kaplan-Meier (KM) estimates will be used to summarize progression free survival (PFS), ADT free survival (ADT-FS), time to locoregional progression (TTLP) and time to distant progression (TTDP), duration of response functions over time. The median PFS, ADT-FS, TTLP and TTDP will be reported.
- The efficacy of SBRT/SABR with oligometastatic disease will also be determined by measuring local control of each lesion at 6-months. Each metastatic lesion will be considered a target lesion and independently

evaluated for response using RECIST 1.1 or bone scan evaluation criteria above. The lesion will be coded as being locally controlled if it is considered stable radiographic disease or if there is evidence of a partial or complete response. Local control assessment will start at three months following randomization and continuous assessment will be pursued during the follow-up period. The proportion of the lesions that have a stable or better response will be estimated using generalized estimating equation.

- Quality of life will be assessed using the Brief Pain Inventory form. An overall score will be calculated pre-treatment and at the time of the 2nd radiologic reassessment. The change in score will be evaluated with a paired t-test.

13.6 Evaluation of Toxicity

All patients who receive at least one fraction of SBRT/SABR will be evaluable for toxicity from the time of their first treatment for SBRT.

13.7 Correlative Science

Descriptive statistics will be performed to correlate the temporal CTC, ctDNA and immune repertoire metric changes with clinical outcomes.

REFERENCES:

1. Hellman S, Weichselbaum RR. Oligometastases. *J Clin Oncol.* 1995 Jan; 13(1): 8-10.
2. Bacci G, Rocca M, Salone M, Balladelli A, Ferrari S, Palmerini E, Forni C, Briccoli A. High grade osteosarcoma of the extremities with lung metastases at presentation: Treatment with neoadjuvant chemotherapy and simultaneous resection of primary and metastatic lesions. *J Surg Oncol.* 2008 Nov 1; 98(6): 415-420.
3. Fong Y, Cohen AM, Fortner JG, Enker WE, Turnbull AD, Coit DG, Marrero AM, Prasad M, Blumgart LH, Brennan MF. Liver resection for colorectal metastases. *J Clin Oncol.* 1997 Mar; 15(3): 938-946.
4. Steele G,Jr, Bleday R, Mayer RJ, Lindblad A, Petrelli N, Weaver D. A prospective evaluation of hepatic resection for colorectal carcinoma metastases to the liver: Gastrointestinal tumor study group protocol 6584. *J Clin Oncol.* 1991 Jul; 9(7): 1105-1112.
5. Simmonds PC, Primrose JN, Colquitt JL, Garden OJ, Poston GJ, Rees M. Surgical resection of hepatic metastases from colorectal cancer: A systematic review of published studies. *Br J Cancer.* 2006 Apr 10; 94(7): 982-999. PMCID: PMC2361241.
6. Schefter TE, Kavanagh BD, Timmerman RD, Cardenes HR, Baron A, Gaspar LE. A phase I trial of stereotactic body radiation therapy (SBRT) for liver metastases. *Int J Radiat Oncol Biol Phys.* 2005 Aug 1; 62(5): 1371-1378.
7. Okunieff P, Petersen AL, Philip A, Milano MT, Katz AW, Boros L, Schell MC. Stereotactic body radiation therapy (SBRT) for lung metastases. *Acta Oncol.* 2006; 45(7): 808-817.
8. Lo SS, Fakiris AJ, Teh BS, Cardenes HR, Henderson MA, Forquer JA, Papiez L, McGarry RC, Wang JZ, Li K, Mayr NA, Timmerman RD. Stereotactic body radiation therapy for oligometastases. *Expert Rev Anticancer Ther.* 2009 May; 9(5): 621-635.
9. Milano MT, Katz AW, Muhs AG, Philip A, Buchholz DJ, Schell MC, Okunieff P. A prospective pilot study of curative-intent stereotactic body radiation therapy in patients with 5 or fewer oligometastatic lesions. *Cancer.* 2008 Feb 1; 112(3): 650-658.
10. Milano MT, Katz AW, Schell MC, Philip A, Okunieff P. Descriptive analysis of oligometastatic lesions treated with curative-intent stereotactic body radiotherapy. *Int J Radiat Oncol Biol Phys.* 2008 Dec 1; 72(5): 1516-1522.
11. Le QT, Loo BW, Ho A, Cotrutz C, Koong AC, Wakelee H, Kee ST, Constantinescu D, Whyte RI, Donington J. Results of a phase I dose-escalation study using single-fraction stereotactic radiotherapy for lung tumors. *J Thorac Oncol.* 2006 Oct; 1(8): 802-809.
12. Gibbs IC, Kamnerdsupal phon P, Ryu MR, Dodd R, Kiernan M, Chang SD, Adler JR,Jr. Image-guided robotic radiosurgery for spinal metastases. *Radiother Oncol.* 2007 Feb; 82(2): 185-190.
13. Lo SS, Fakiris AJ, Abdulrahman R, Henderson MA, Chang EL, Suh JH, Timmerman RD. Role of stereotactic radiosurgery and fractionated stereotactic radiotherapy in pediatric brain tumors. *Expert Rev Neurother.* 2008 Jan; 8(1): 121-132.
14. Levine AM, Coleman C, Horasek S. Stereotactic radiosurgery for the treatment of primary sarcomas and sarcoma metastases of the spine. *Neurosurgery.* 2009 Feb; 64(2 Suppl): A54-9.
15. Goodman KA, Wiegner EA, Maturen KE, Zhang Z, Mo Q, Yang G, Gibbs IC, Fisher GA, Koong AC. Dose-escalation study of single-fraction stereotactic body radiotherapy for liver malignancies. *Int J Radiat Oncol Biol Phys.* 2010 Oct 1; 78(2): 486-493.
16. Onishi H, Shirato H, Nagata Y, Hiraoka M, Fujino M, Gomi K, Niibe Y, Karasawa K, Hayakawa K, Takai Y, Kimura T, Takeda A, Ouchi A, Hareyama M, Kokubo M, Hara R, Itami J, Yamada K, Araki T. Hypofractionated stereotactic radiotherapy (HypoFXSRT) for stage I non-small cell lung cancer: Updated results of 257 patients in a japanese multi-institutional study. *J Thorac Oncol.* 2007 Jul; 2(7 Suppl 3): S94-100.
17. Baumann P, Nyman J, Hoyer M, Wennberg B, Gagliardi G, Lax I, Drugge N, Ekberg L, Friesland S, Johansson KA, Lund JA, Morhed E, Nilsson K, Levin N, Paludan M, Sederholm C, Traberg A, Wittgren L, Lewensohn R. Outcome in a prospective phase II trial of medically inoperable stage I non-small-cell lung cancer patients treated with stereotactic body radiotherapy. *J Clin Oncol.* 2009 Jul 10; 27(20): 3290-3296.
18. Timmerman R, Paulus R, Galvin J, Michalski J, Straube W, Bradley J, Fakiris A, Bezjak A, Videtic G, Johnstone D, Fowler J, Gore E, Choy H. Stereotactic body radiation therapy for inoperable early stage lung cancer. *JAMA.* 2010 Mar 17; 303(11): 1070-1076. PMCID: PMC2907644.

19. Videtic GM, Stephans K, Reddy C, Gajdos S, Kolar M, Clouser E, Djemil T. Intensity-modulated radiotherapy-based stereotactic body radiotherapy for medically inoperable early-stage lung cancer: Excellent local control. *Int J Radiat Oncol Biol Phys.* 2010 Jun 1; 77(2): 344-349.
20. Fakiris AJ, McGarry RC, Yiannoutsos CT, Papiez L, Williams M, Henderson MA, Timmerman R. Stereotactic body radiation therapy for early-stage non-small-cell lung carcinoma: Four-year results of a prospective phase II study. *Int J Radiat Oncol Biol Phys.* 2009 Nov 1; 75(3): 677-682.
21. Grills IS, Mangona VS, Welsh R, Chmielewski G, McInerney E, Martin S, Wloch J, Ye H, Kestin LL. Outcomes after stereotactic lung radiotherapy or wedge resection for stage I non-small-cell lung cancer. *J Clin Oncol.* 2010 Feb 20; 28(6): 928-935.
22. Wulf J, Haedinger U, Oppitz U, Thiele W, Mueller G, Flentje M. Stereotactic radiotherapy for primary lung cancer and pulmonary metastases: A noninvasive treatment approach in medically inoperable patients. *Int J Radiat Oncol Biol Phys.* 2004 Sep 1; 60(1): 186-196.
23. Hof H, Hoess A, Oetzel D, Debus J, Herfarth K. Stereotactic single-dose radiotherapy of lung metastases. *Strahlenther Onkol.* 2007 Dec; 183(12): 673-678.
24. Kim TH, Kim DY, Park JW, Kim YI, Kim SH, Park HS, Lee WJ, Park SJ, Hong EK, Kim CM. Three-dimensional conformal radiotherapy of unresectable hepatocellular carcinoma patients for whom transcatheter arterial chemoembolization was ineffective or unsuitable. *Am J Clin Oncol.* 2006 Dec; 29(6): 568-575.
25. Zhou ZH, Liu LM, Chen WW, Men ZQ, Lin JH, Chen Z, Zhang XJ, Jiang GL. Combined therapy of transcatheter arterial chemoembolisation and three-dimensional conformal radiotherapy for hepatocellular carcinoma. *Br J Radiol.* 2007 Mar; 80(951): 194-201.
26. Seo DD, Lee HC, Jang MK, Min HJ, Kim KM, Lim YS, Chung YH, Lee YS, Suh DJ, Ko GY, Lee YJ, Lee SG. Preoperative portal vein embolization and surgical resection in patients with hepatocellular carcinoma and small future liver remnant volume: Comparison with transarterial chemoembolization. *Ann Surg Oncol.* 2007 Dec; 14(12): 3501-3509.
27. Kamel IR, Liapi E, Reyes DK, Zahurak M, Bluemke DA, Geschwind JF. Unresectable hepatocellular carcinoma: Serial early vascular and cellular changes after transarterial chemoembolization as detected with MR imaging. *Radiology.* 2009 Feb; 250(2): 466-473.
28. Lo SS, Teh BS, Mayr NA, Olencki TE, Wang JZ, Grecula JC, Lu JJ, Timmerman RD. Stereotactic body radiation therapy for oligometastases. *Discov Med.* 2010 Sep; 10(52): 247-254.
29. Ben-Josef E, Lawrence TS. Using a bigger hammer: The role of stereotactic body radiotherapy in the management of oligometastases. *J Clin Oncol.* 2009 Apr 1; 27(10): 1537-1539.
30. Christodouleas JP, Marks LB. Analysis of patients with oligometastases undergoing two or more curative-intent stereotactic radiotherapy courses: In regard to milano et al. (int J radiat oncol biol phys 2009;73:832-837). *Int J Radiat Oncol Biol Phys.* 2009 Aug 1; 74(5): 1628; author reply 1628-9.
31. Kavanagh BD, McGarry RC, Timmerman RD. Extracranial radiosurgery (stereotactic body radiation therapy) for oligometastases. *Semin Radiat Oncol.* 2006 Apr; 16(2): 77-84.
32. Kelloff GJ, Krohn KA, Larson SM, et al. The progress and promise of molecular imaging probes in oncologic drug development. Clin Cancer Res. Nov 15 2005;11(22):7967-7985.
33. Shankar LK, Hoffman JM, Bacharach S, et al. Consensus recommendations for the use of 18F-FDG PET as an indicator of therapeutic response in patients in National Cancer Institute Trials. J Nucl Med. Jun 2006;47(6):1059-1066.
34. Turlakow A, Larson SM, Coakley F, et al. Local detection of prostate cancer by positron emission tomography with 2-fluorodeoxyglucose: comparison of filtered back projection and iterative reconstruction with segmented attenuation correction. Q J Nucl Med. Sep 2001;45(3):235-244.
35. Hofer C, Laubenbacher C, Block T, Breul J, Hartung R, Schwaiger M. Fluorine-18-fluorodeoxyglucose positron emission tomography is useless for the detection of local recurrence after radical prostatectomy. European urology. 1999;36(1):31-35.
36. Nunez R, Macapinlac HA, Yeung HW, et al. Combined 18F-FDG and 11C-methionine PET scans in patients with newly progressive metastatic prostate cancer. J Nucl Med. Jan 2002;43(1):46-55.
37. Shreve PD, Grossman HB, Gross MD, Wahl RL. Metastatic prostate cancer: initial findings of PET with 2-deoxy-2-[F-18]fluoro-D-glucose. Radiology. Jun 1996;199(3):751-756.
38. Hricak H, Choyke PL, Eberhardt SC, Leibel SA, Scardino PT. Imaging prostate cancer: a multidisciplinary perspective. Radiology. Apr 2007;243(1):28-53.

39. Lawrentschuk N, Davis ID, Bolton DM, Scott AM. Positron emission tomography and molecular imaging of the prostate: an update. BJU international. May 2006;97(5):923-931.
40. Scattoni V, Picchio M, Suardi N, et al. Detection of lymph-node metastases with integrated [11C]choline PET/CT in patients with PSA failure after radical retropubic prostatectomy: results confirmed by open pelvic-retroperitoneal lymphadenectomy. European urology. Aug 2007;52(2):423-429.
41. de Jong IJ, Pruim J, Elsinga PH, Vaalburg W, Mensink HJ. 11C-choline positron emission tomography for the evaluation after treatment of localized prostate cancer. European urology. Jul 2003;44(1):32-38; discussion 38-39.
42. Picchio M, Messa C, Landoni C, et al. Value of [11C]choline-positron emission tomography for re-staging prostate cancer: a comparison with [18F]fluorodeoxyglucose-positron emission tomography. The Journal of urology. Apr 2003;169(4):1337-1340.
43. Morris MJ, Scher HI. (11)C-acetate PET imaging in prostate cancer. European journal of nuclear medicine and molecular imaging. Feb 2007;34(2):181-184.
44. Even-Sapir E, Metser U, Mishani E, Lievshitz G, Lerman H, Leibovitch I. The detection of bone metastases in patients with high-risk prostate cancer: 99mTc-MDP Planar bone scintigraphy, single- and multi-field-of-view SPECT, 18F-fluoride PET, and 18F-fluoride PET/CT. J Nucl Med. Feb 2006;47(2):287-297.
45. Even-Sapir E, Mishani E, Flusser G, Metser U. 18F-Fluoride positron emission tomography and positron emission tomography/computed tomography. Semin Nucl Med. Nov 2007;37(6):462-469.
46. Beattie BJ, Smith-Jones PM, Jhanwar YS, et al. Pharmacokinetic assessment of the uptake of 16beta-18F-fluoro-5alpha-dihydrotestosterone (FDHT) in prostate tumors as measured by PET. J Nucl Med. Feb 2011;51(2):183-192.
47. Schuster DM, Savir-Baruch B, Nieh PT, et al. Detection of recurrent prostate carcinoma with anti-1-amino-3-18F-fluorocyclobutane-1-carboxylic acid PET/CT and 111In-capromab pendetide SPECT/CT. Radiology. Jun 2011;259(3):852-861.
48. Schuster DM, Votaw JR, Nieh PT, et al. Initial experience with the radiotracer anti-1-amino-3-18F-fluorocyclobutane-1-carboxylic acid with PET/CT in prostate carcinoma. J Nucl Med. Jan 2007;48(1):56-63.
49. Greco C, Cascini GL, Tamburrini O. Is there a role for positron emission tomography imaging in the early evaluation of prostate cancer relapse? Prostate cancer and prostatic diseases. 2008;11(2):121-128.
50. Bostwick DG, Pacelli A, Blute M, Roche P, Murphy GP. Prostate specific membrane antigen expression in prostatic intraepithelial neoplasia and adenocarcinoma: a study of 184 cases. Cancer. Jun 1 1998;82(11):2256-2261.
51. Chang SS, Reuter VE, Heston WD, Gaudin PB. Comparison of anti-prostate-specific membrane antigen antibodies and other immunomarkers in metastatic prostate carcinoma. Urology. Jun 2001;57(6):1179-1183.
52. Sweat SD, Pacelli A, Murphy GP, Bostwick DG. Prostate-specific membrane antigen expression is greatest in prostate adenocarcinoma and lymph node metastases. Urology. Oct 1998;52(4):637-640.
53. Wright GL, Jr., Grob BM, Haley C, et al. Upregulation of prostate-specific membrane antigen after androgen-deprivation therapy. Urology. Aug 1996;48(2):326-334.
54. Perner S, Hofer MD, Kim R, et al. Prostate-specific membrane antigen expression as a predictor of prostate cancer progression. Human pathology. May 2007;38(5):696-701.
55. Ross JS, Sheehan CE, Fisher HA, et al. Correlation of primary tumor prostate-specific membrane antigen expression with disease recurrence in prostate cancer. Clin Cancer Res. Dec 15 2003;9(17):6357-6362.
56. Thomas CT, Bradshaw PT, Pollock BH, et al. Indium-111-capromab pendetide radioimmunoscintigraphy and prognosis for durable biochemical response to salvage radiation therapy in men after failed prostatectomy. J Clin Oncol. May 1 2003;21(9):1715-1721.
57. Wilkinson S, Chodak G. The role of 111indium-capromab pendetide imaging for assessing biochemical failure after radical prostatectomy. The Journal of urology. Jul 2004;172(1):133-136.
58. Barinka C, Rovenska M, Mlcochova P, et al. Structural insight into the pharmacophore pocket of human glutamate carboxypeptidase II. Journal of medicinal chemistry. Jul 12 2007;50(14):3267-3273.
59. Barinka C, Starkova J, Konvalinka J, Lubkowski J. A high-resolution structure of ligand-free human glutamate carboxypeptidase II. Acta crystallographica. Mar 1 2007;63(Pt 3):150-153.

60. Foss CA, Mease RC, Fan H, et al. Radiolabeled small-molecule ligands for prostate-specific membrane antigen: in vivo imaging in experimental models of prostate cancer. Clin Cancer Res. Jun 1 2005;11(11):4022-4028.
61. Mease RC, Dusich CL, Foss CA, et al. N-[N-[(S)-1,3-Dicarboxypropyl]carbamoyl]-4-[18F]fluorobenzyl-L-cysteine, [18F]DCFBC: a new imaging probe for prostate cancer. Clin Cancer Res. May 15 2008;14(10):3036-3043.
62. Cho SY, Gage KL, Mease RC, et al. Biodistribution, Tumor Detection and Radiation Dosimetry of N-[N-[(S)-1,3-dicarboxypropyl]carbamoyl]-4-18F-fluorobenzyl-L-cysteine (18F-DCFBC), a Low Molecular Weight Inhibitor of PSMA, in Patients with Metastatic Prostate Cancer. J Nucl Med. 2012 Dec;53(12):1883-91.
63. Szabo Z, Mena E, Rowe SP, et al. Initial Evaluation of [(18)F]DCFPyL for Prostate-Specific Membrane Antigen (PSMA)-Targeted PET Imaging of Prostate Cancer. Mol Imaging Biol. 2015 Aug;17(4):565-74.
64 Chiang, A. C. & Massague, J. Molecular basis of metastasis. *N Engl J Med* **359**, 2814-2823, doi:10.1056/NEJMra0805239359/26/2814 [pii] (2008).
65 Kim, M. Y. *et al.* Tumor self-seeding by circulating cancer cells. *Cell* **139**, 1315-1326, doi:10.1016/j.cell.2009.11.025S0092-8674(09)01437-8 [pii] (2009).
66 Comen, E., Norton, L. & Massague, J. Clinical implications of cancer self-seeding. *Nat Rev Clin Oncol* **8**, 369-377, doi:10.1038/nrclinonc.2011.64nrclinonc.2011.64 [pii] (2011).
67 Gundem, G. *et al.* The evolutionary history of lethal metastatic prostate cancer. *Nature* **520**, 353-357, doi:10.1038/nature14347nature14347 [pii] (2015).
68 Marrinucci, D. *et al.* Fluid biopsy in patients with metastatic prostate, pancreatic and breast cancers. *Phys Biol* **9**, 016003, doi:10.1088/1478-3975/9/1/016003S1478-3975(12)97631-6 [pii] (2012).
69 Newman, A. M. *et al.* An ultrasensitive method for quantitating circulating tumor DNA with broad patient coverage. *Nature medicine*, doi:10.1038/nm.3519nm.3519 [pii] (2014).
70 Robins, H. S. *et al.* Comprehensive assessment of T-cell receptor beta-chain diversity in alphabeta T cells. *Blood* **114**, 4099-4107, doi:10.1182/blood-2009-04-217604blood-2009-04-217604 [pii] (2009).
71 Cha, E. *et al.* Improved survival with T cell clonotype stability after anti-CTLA-4 treatment in cancer patients. *Sci Transl Med* 6, 238ra270, doi:10.1126/scitranslmed.30082116/238/238ra70 [pii] (2014).
72 Keizman D, Zahurak M, Sinibaldi V, et. al. Lenalidomide in nonmetastatic biochemically relapsed prostate cancer: results of a phase I/II double-blinded, randomized study. Clin Cancer Res. 2010 Nov 1;16(21):5269-76.
73. Pound C, Partin A, Eisenberger M, Chan DW, Pearson JD, Walsh PC. Natural history of progression after PSA elevation following radical prostatectomy. JAMA 1999;281:1591–7.
74. Antonarakis ES, Trock BJ, Feng Z, et al. The natural history of metastatic progression in men with PSA-recurrent prostate cancer after radical prostatectomy: 25-year follow-up. J Clin Oncol 2009;27: 15s, (suppl; abstr 5008.
75. Scher HI, Halabi S, Tannock I, et. al. Design and End Points of Clinical Trials for Patients With Progressive Prostate Cancer and Castrate Levels of Testosterone: Recommendations of the Prostate Cancer Clinical Trials Working Group. J Clin Oncol. 2008 Mar 1;26(7):1148-59.
76. Decaestecker K, De Meerleer G, Lambert B, *et al.* Repeated stereotactic body radiotherapy for oligometastatic prostate cancer recurrence. Radiat Oncol. 2014 Jun 12;9:135.
77. Vickers AJ. How to randomize. J Soc Integr Oncol 2006;4:194-8.
78. Zeng, J., Harris, T. J., Lim, M., Drake, C. G. & Tran, P. T. Immune modulation and stereotactic radiation: improving local and abscopal responses. *Biomed Res Int* **2013**, 658126, doi:10.1155/2013/658126 (2013).

APPENDIX I: Brief Pain Inventory Form

STUDY ID# ____________ HOSPITAL # ____________

DO NOT WRITE ABOVE THIS LINE

Brief Pain Inventory (Short Form)

Date: _____/_____ /_____ Time: _____

Name: ____________________ ____________________ ____________________

Last First Middle Initial

1. Throughout our lives, most of us have had pain from time to time (such as minor headaches, sprains, and toothaches). Have you had pain other than these every-day kinds of pain today?

   1. Yes 2. No

2. On the diagram, shade in the areas where you feel pain. Put an X on the area that hurts the most.

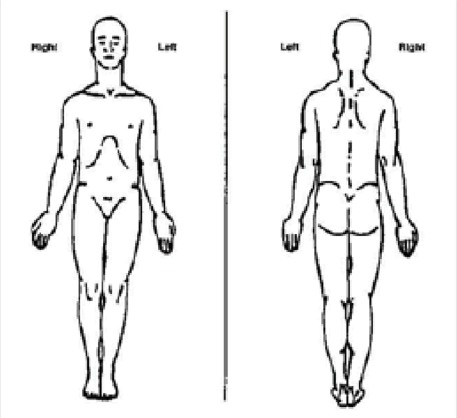

3. Please rate your pain by circling the one number that best describes your pain at its worst in the last 24 hours.

0	1	2	3	4	5	6	7	8	9	10
No Pain										Pain as bad as you can imagine

4. Please rate your pain by circling the one nuimber that best describes your pain at its least in the last 24 hours.

0	1	2	3	4	5	6	7	8	9	10
No Pain										Pain as bad as you can imagine

5. Please rate your pain by circling the one number that best describes your pain on the average.

0	1	2	3	4	5	6	7	8	9	10
No Pain										Pain as bad as you can imagine

6. Please rate your pain by circling the one number that tells how much pain you have right now.

0	1	2	3	4	5	6	7	8	9	10
No Pain										Pain as bad as you can imagine

7. What treatments or medications are you receiving for your pain?

____

____

8. In the last 24 hours, how much relief have pain treatments or medications provided? Please circle the one percentage that most shows how much relief you have received.

0%	10%	20%	30%	40%	50%	60%	70%	80%	90%	100%
No Relief										Complete Relief

9. Circle the one number that describes how, during the past 24 hours, pain has interfered with your:

A. General Activity

0	1	2	3	4	5	6	7	8	9	10
Does not Interfere										Completely Interferes

B. Mood

0	1	2	3	4	5	6	7	8	9	10
Does not Interfere										Completely Interferes

C. Walking Ability

0	1	2	3	4	5	6	7	8	9	10
Does not Interfere										Completely Interferes

D. Normal Work (includes both work outside the home and housework)

0	1	2	3	4	5	6	7	8	9	10
Does not Interfere										Completely Interferes

E. Relations with other people

0	1	2	3	4	5	6	7	8	9	10
Does not Interfere										Completely Interferes

F. Sleep

0	1	2	3	4	5	6	7	8	9	10
Does not Interfere										Completely Interferes

G. Enjoyment of life

0	1	2	3	4	5	6	7	8	9	10
Does not Interfere										Completely Interferes

Copyright 1991 Charles S. Cleeland, PhD
Pain Research Group
All rights reserved.
Used by permission.

APPENDIX II: Performance Status Criteria

**ECOG Performance Status Scale**		**Karnofsky Performance Scale**	
Grade	Descriptions	Percent	Description
0	Normal activity. Fully active, able to carry on all pre-disease performance without restriction.	100	Normal, no complaints, no evidence of disease.
		90	Able to carry on normal activity; minor signs or symptoms of disease.
1	Symptoms, but ambulatory. Restricted in physically strenuous activity, but ambulatory and able to carry out work of a light or sedentary nature (e.g., light housework, office work).	80	Normal activity with effort; some signs or symptoms of disease.
		70	Cares for self, unable to carry on normal activity or to do active work.
2	In bed <50% of the time. Ambulatory and capable of all self-care, but unable to carry out any work activities. Up and about more than 50% of waking hours.	60	Requires occasional assistance, but is able to care for most of his/her needs.
		50	Requires considerable assistance and frequent medical care.
3	In bed >50% of the time. Capable of only limited self-care, confined to bed or chair more than 50% of waking hours.	40	Disabled, requires special care and assistance.
		30	Severely disabled, hospitalization indicated. Death not imminent.
4	100% bedridden. Completely disabled. Cannot carry on any self-care. Totally confined to bed or chair.	20	Very sick, hospitalization indicated. Death not imminent.
		10	Moribund, fatal processes progressing rapidly.
5	Dead.	0	Dead.

APPENDIX III: CAPP-Seq Sample Collection & Processing

Diehn Lab Blood Processing Protocol (version 122314)

❖ **Materials** (check to make sure you have all of it before you start)
- Filtered P2000 pipette tips
- P-2000 pipette
- 2.0-mL Eppendorf tubes
- Patient's blood - usually 30 mL in 3 "purple top" tubes (EDTA) per time point. Blood should be kept on ice or in refrigerator after drawing and processed as soon as possible to minimize lysis of WBCs and release of cellular genomic DNA into plasma

❖ **Methods**
1. Spin samples in the clinical centrifuge using the settings: 3,500rpm, 10min, 4C
2. While you wait, label tubes
3. After spinning, carefully remove lavender top tubes from centrifuge. Do not disturb the separated plasma and cell-free whole blood
    i. Tip: It helps to put all the tubes into one holder and carefully carry the holder to the hood
4. Using a filtered tip and p-2000 pipet aliquot ~1.8 mL clear plasma (not all the way to the top since tops of tubes tend to pop open upon freezing if filled all the way) into a 2.0 mL Eppendorf tube. Repeat until you have aliquotted the plasma from all the purple-top tubes into 2.0 mL Eppendorf tubes. With the tubes that have only the buffy coat and RBC remaining, mix the buffy coat and cell-free whole blood using a pipette tip and aliquot ~1.8 mL into a 2.0 mL Eppendorf tube. Repeat so you have a second 2.0 mL Eppendorf tube containing the buffy coat and RBC mixed together.
5. Put Eppendorf tubes into -80C freezer.
6. Write down **# of plasma tubes stored, box number, date of blood draw, time of storage** on the top of patients' requisition forms.
7. Enter the blood draw information into RedCap database.

APPENDIX VI: EPIC-HD CTC Sample Collection & Processing

EPIC SCIENCES

EPIC SCIENCES, INC.
9381 Judicial Dr, Suite 200
San Diego, CA 92121

CTC Sample Collection in Streck Cell-Free DNA BCT Tubes

IMPORTANT: The first 5 mL of blood collected from the fresh venipuncture **cannot** be used for the collection into the Streck tubes due to possibility of contaminating epithelial cells during venipuncture. **Please ensure that at least one blood tube of 5 mL or more is collected prior to collection of the CTC sample to avoid adversely affecting the test results.**

Prevention of Backflow:

Since Streck Cell-Free DNA BCT tubes contain chemical additives, it is important to avoid possible backflow from the tube. To guard against backflow, observe the following precautions:

- Keep patient's arm in the downward position during the collection procedure.
- Hold the tube with the stopper uppermost.
- Release tourniquet once the blood starts to flow into the tube, or within 2 minutes of application.
- Tube contents should not touch stopper or the end of the needle during the collection procedure.

Blood Collection Instructions:

**Schedule courier for same-day sample pic-up prior to collection

1. Confirm blood tube is not expired. Expired tubes should not be used for blood collection.
2. Draw whole blood sample into 10 mL Streck Cell-Free DNA BCT tube (*see note regarding prevention of backflow). Fill tube until blood flow stops. NOTE: Epic requires a minimum of 4mL blood per sample, but a full 10 mL tube of blood should be provided when possible.
3. Remove tube from adapter and immediately mix by gentle inversion 8 to 10 times. Tube inversion prevents clotting. Inadequate or delayed mixing may result in inaccurate test results.
4. Label the tube with subject's identification and date and time of blood draw.
5. Keep sample at ***room temperature*** and ***ship on day of collection.***

Sample Shipment Instructions:

Samples are processed the same day as receipt. Priority is given to the samples that had notification the day prior. Epic is able to process samples up to 96 hours after the initial blood draw, but it is preferable to process the blood before the 48 hour mark for optimal CTC retention and data integrity purposes.

EPIC SCIENCES

EPIC SCIENCES, INC.
9381 Judicial Dr, Suite 200
San Diego, CA 92121

The total number of slides created is dependent upon the patient's WBC count and amount of blood provided. Each tube should be labeled with the Study and Subject ID. Each sample sent should be accompanied by a requisition form, filled out by the site, listing the study ID, subject ID, draw date, draw time, and time point (if applicable). To avoid weekend delays, mark Saturday delivery if shipping on Friday.

Samples should be sent overnight in ambient shippers on the day the blood sample is drawn.

Epic Sciences receives samples Monday through Saturday from 8:00 a.m. to 5:00 p.m., excluding U.S. holidays. Incoming sample notifications should be sent during normal business hours on the day of shipment by email to partners@epicsciences.com or by fax at 858-356-5852. For samples that will be processed at LabCorp Belgium, please send the sample notification email to partners.eu@epicsciences.com. For samples that will be processed at LabCorp Singapore, please send the sample notification email to LabCorp.Singapore@epicsciences.com.

This email/fax should include:

1) Study ID
2) Subject ID (and timepoint if applicable)
3) Tracking Number
4) Number of samples being shipped
5) Date and time of each blood sample draw

APPENDIX V: ImmunoSEQ Sample Collection & Processing

immunoSEQ® Service

amplify discovery™

Human Sample Preparation Guidelines

Sample Types Accepted
- Sorted T and B cells
- Peripheral blood mononucleated cells (PBMCs)
- Whole blood
- Tissue (including Fresh Frozen [FF] or formalin-fixed, paraffin-embedded [FFPE] tissue)
- gDNA and cDNA

Assays available:
- T-cell receptor beta (TCRB)
- T-cell receptor alpha/delta (TCRA/D)
- T-cell receptor gamma (TCRG)
- IGH
- IG kappa/lambda (IGK/L)

TCR IMMUNOSEQ ASSAY SAMPLE GUIDELINES

Sample type	Resolution			
	Survey	Deep	Ultra Deep	Max Depth
Sorted T cells	60,000 cells[a] 1 µg DNA 50 µL vol.	200,000 cells 3 µg DNA 125 µL vol.	800,000 cells 12 µg DNA 400 µL vol.	4,000,000 cells 48 µg DNA 700 µL vol.
PBMCs	120,000 cells 2 µg DNA 50 µL vol.	400,000 cells 6 µg DNA 125 µL vol.	1,600,000 cells 24 µg DNA 400 µL vol.	8,000,000 cells 96 µg DNA 700 µL vol.
Whole blood	2 mL blood 4 µg DNA	4 mL blood 12 µg DNA	10 mL blood 48 µg DNA	Contact Technical Support
Lymphoid tissue[b]	25 microns FFPE 10 mg FF tissue 1 µg DNA	3 µg DNA	Contact Technical Support	
Non-lymphoid tissue	25 microns FFPE 10 mg FF tissue 3 µg DNA	9 µg DNA	Not recommended	

[a] 1000 cells is the absolute minimum number of cells accepted.
[b] Deep resolution is recommended for lymphoid tissue samples.

NOTE: In order to sequence only rearranged TCRG receptors originating from gamma/delta T cells in PBMCs and tissue samples, a cell sort must be performed prior to receipt of samples by Adaptive Biotechnologies. Unsorted cells will result in the amplification of rearranged TCRG receptors from both gamma/delta and alpha/beta T cells.

cDNA GUIDELINES

- A minimum of 150 ng of RNA is recommended as starting material for the reverse transcription step
- Adaptive targets 10-fold sequencing coverage of each TCR or BCR template
- Using cDNA as an input source will limit available applications

BCR IMMUNOSEQ ASSAY SAMPLE GUIDELINES

Sample type	Resolution			
	Survey	Deep	Ultra Deep	Max Depth
Sorted B cells	60,000 cells[a] 1 µg DNA 50 µL vol.	200,000 cells 3 µg DNA 125 µL vol.	800,000 cells 12 µg DNA 400 µL vol.	4,000,000 cells 48 µg DNA 700 µL vol.
PBMCs/bone marrow[b]	600,000 cells 4 µg DNA 50 µL vol.	2,000,000 cells 12 µg DNA 125 µL vol.	Contact Technical Support	
Lymphoid tissue	10 mg FF tissue 1 µg DNA	3 µg DNA		
Non-lymphoid tissue	10 mg FF tissue 3 µg DNA	9 µg DNA	Not recommended	

[a] 1000 cells is the absolute minimum number of cells accepted.
[b] B cells represent a small fraction of the total PBMC population; this resolution may not be appropriate for all projects.

DESCRIPTION OF PROFILING RESOLUTIONS: SURVEY VS. DEEP

Resolution	Considerations for choosing resolution
Survey	• Clonal samples<br>• Samples with low numbers of T or B cells (<100,000 estimated T or B cells cells)<br>• Samples derived from most non-lymphoid tissues
Deep	• Studying the peripheral immune repertoire (e.g. whole blood, peripheral blood mononuclear cells [PBMCs], or lymphoid tissue)<br>• Samples requiring greater sensitivity (detection of rare clones)<br>• Experiments assessing a broader range of the repertoire

amplify **discovery**™

RECOMMENDATIONS FOR SAMPLE PREPARATION

Isolating DNA from different sample types

Sorted cells

- Sorting fixed cells into HEPES buffer (PBS with 2% FBS and 0.025M HEPES) can boost the DNA yield from the cell pellets
- When preparing fixed cells for fluorescence-activated cell sorting (FACS), a concentration of 0.5%–2.0% PFA is recommended. Higher concentrations of PFA can fragment the DNA, which will result in reduced PCR amplification efficiency
- Cells should arrive in no more than 200 µL of buffer

Tissue

- A tissue homogenizer with homogenization buffer is recommended for disruption of fresh or frozen tissue samples
- Example kit for DNA extraction:
  - Qiagen DNeasy™ Blood & Tissue Kit (Mini Spin Columns)
  - Qiagen QIAamp™ DNA FFPE Tissue Kit

Blood, PBMCs, or bone marrow

- ACD or EDTA is recommended as anticoagulant for whole blood or bone marrow collection
- Sodium heparin and sodium citrate are compatible with the immunoSEQ Assay. However, excessive amounts of sodium heparin can inhibit PCR
- Roughly 50% of cells frozen in DMSO will lyse during the thawing process. To recover all DNA do not centrifuge the sample after thawing. Instead, extract DNA from the entire thawed sample
- Possible extraction kits:
  - Qiagen DNeasy™ Blood & Tissue Kit (Mini Spin Columns): Bone marrow and <1 mL blood
  - Qiagen QIAamp™ DNA Blood Maxi Kit: 1–10 mL blood

Shipping Samples

- Cells and tissue: 1.5 or 2.0 mL Eppendorf tubes/ cryotubes (snap top or screw cap)
- Whole blood: Vacutainer ACD or EDTA tube, completely filled
- gDNA or cDNA: Uniquely barcoded sample containers will be provided by Adaptive Biotechnologies.

Quality of input DNA

Once DNA is isolated, quantification using a spectrophotometer or comparable method is highly recommended. For optimal results the absorbance ratios of DNA samples should be:

- A260/280 = 1.8–2.0
- A260/230 = 2.0–2.2

Potential PCR inhibitors

Sample source(s) containing any of the following may inhibit PCR steps used in the immunoSEQ Assay:

- **Heparin, EDTA,** common anticoagulants in blood and bone marrow samples
- **Melanin,** common to skin and melanoma tissue samples
- **B5 Reagent,** commonly used for bone marrow storage
- **Collagen,** can be at high levels in some tissue samples
- **Myoglobin,** common to muscle tissue
- **Bacterial contamination** from all sample sources
- **Phenol, ethanol,** and **other organic contaminants** remaining after DNA extraction

For questions or Technical Support contact:
techsupport@adaptivebiotech.com, or (855) 466-8667

For Research Use Only. Not for use in diagnostic procedures.
Copyright © 2015 Adaptive Biotechnologies Corp. All rights reserved.
MRK-10003-01 AC

Adaptive biotechnologies®
adaptivebiotech.com

Phase II Randomized Observation versus Stereotactic Ablative RadiatIon for OLigometastatic Prostate CancEr (ORIOLE) Trial

Protocol Number J15180
IND Number # 121,064

Principal Investigator
Phuoc T. Tran, M.D., PhD
Sidney Kimmel Comprehensive Cancer Center
Johns Hopkins University
Weinberg Building
1550 Orleans St., CRB 2 Rm 406
Baltimore, MD 21231
Phone: 410-614-3880
Fax: 410-502-1419
Email: tranp@jhmi.edu

Co-Investigators
Theodore DeWeese, M.D.
Danny Song, M.D.
Mario Eisenberger, MD
Ashley Ross, MD, PhD
Michael Gorin, M.D.
Michael Carducci, M.D.
Emmanuel Antonarakis, M.D.
Channing Paller, M.D.
Kenneth Pienta, M.D.
Lawrence Kleinberg, M.D.
Kristin Redmond, M.D.
Steve Rowe, M.D., PhD
Martin Pomper, M.D., PhD
Stephen Greco, M.D.
Curtiland Deville, M.D.

Statisticians
Hao Wang, PhD
Bruce Trock, PhD

Research Nurse
Shirl Dipasquale

Research Managers:
Helen Kim
Terry Caldwell

Study Coordinator:
Noura Radwan, M.D.

Funding:
NCI 1U01CA183031-01A1 (Pomper/DeWeese)
Movember-PCF Challenge Award (Tran/Ross/Dicker)

Table of Contents

SCHEMA 4

1. PROTOCOL SYNOPSIS 5
2. ABBREVIATIONS AND DEFINITIONS OF TERMS 7
3. STUDY DESIGN AND OBJECTIVES 8
   - 3.1 STUDY DESIGN 8
   - 3.2 PRIMARY OBJECTIVE 8
   - 3.3 SECONDARY OBJECTIVES 8
   - 3.4 CORRELATIVE OBJECTIVES 8
4. BACKGROUND 9
   - 4.1 OLIGOMETASTATIC DISEASE 9
   - 4.2 STEREOTACTIC BODY RADIATION THERAPY (SBRT) OR STEREOTACTIC ABLATIVE RADIATION THERAPY (SABR) 9
   - 4.3 RATIONALE FOR USE OF SBRT/SABR IN OLIGOMETASTATIC DISEASE 10
   - 4.4 RATIONALE FOR SBRT/SABR FOR BONE OLIGOMETASTASES IN PROSTATE CANCER 10
   - 4.5 RATIONALE FOR PROSTATE SPECIFIC MEMBRANE ANTIGEN (PSMA) FUNCTIONAL IMAGING TO HELP REFINE SELECTION OF BONE LESIONS TO TARGET WITH SBRT/SABR IN OLIGOMETASTATIC PROSTATE CANCER 11
   - 4.6 RATIONALE FOR CORRELATIVE SCIENCE 14
   - 4.7 RATIONAL FOR COLOR GENOMICS 17
5. PARTICIPANT SELECTION AND ENROLLMENT PROCEDURES 18
   - 5.1 INCLUSION CRITERIA 18
   - 5.2 EXCLUSION CRITERIA 19
6. TREATMENT PLAN 19
   - 6.1 RANDOMIZATION 19
   - 6.2 DIAGNOSTIC PROCEDURES 20
   - 6.3 THERAPEUTIC PROCEDURES 20
   - 6.4 FOLLOW-UP PROCEDURES 23
   - 6.5 DURATION OF THERAPY 25
   - 6.6 DURATION OF STUDY ACCRUAL 25
   - 6.7 DURATION OF FOLLOW-UP 25
   - 6.8 CRITERIA FOR PATIENT REMOVAL 25
   - 6.9 ALTERNATIVES 25
   - 6.10 COSTS 25
   - 6.11 COMPENSATION 25
   - 6.12 WITHDRAWAL FROM STUDY 26
   - 6.13 CROSS-OVER 26
7. INVESTIGATIONAL AGENT/DEVICE/PROCEDURE INFORMATION 26
   - 7.1 INVESTIGATIONAL AGENT - DCFPYL 26
8. STUDY CALENDARS 27
   - STUDY CALENDAR: OBSERVATION ARM ONLY 27
   - STUDY CALENDAR: SBRT ARM ONLY 28
9. MEASUREMENT OF EFFECT 29
   - 9.1 ANTITUMOR EFFECT – METASTATIC TUMORS 29

9.2 DEFINITIONS 29
9.3 DISEASE PARAMETERS 29
9.4 METHODS FOR EVALUATION OF MEASURABLE DISEASE 30
9.5 RESPONSE CRITERIA 31
9.6 IMAGING REFERENCE STANDARD, AS APPLICABLE (I.E., "GOLD STANDARD") 31

10. ADVERSE EVENT REPORTING REQUIREMENTS 33

10.1 GENERAL 33
10.2 DEFINITIONS 33
10.3 POTENTIAL ADVERSE EVENTS 34
10.4 STEREOTACTIC BODY RADIATION TREATMENT (SBRT) OR STEREOTACTIC ABLATIVE RADIATION TREATMENT (SABR) 35
10.5 DCFPYL 35
10.6 REPORTING PROCEDURES 39

11. DATA AND SAFETY MONITORING PLAN 41

12. REGULATORY CONSIDERATIONS 42

12.1 PROTOCOL REVIEW AND AMENDMENTS 42
12.2 INFORMED CONSENT 42
12.3 ETHICS AND GCP 42

13. STATISTICAL CONSIDERATIONS 43

13.1 ENDPOINTS 43
13.2 SAMPLE SIZE/ACCRUAL RATE 44
13.3 EARLY STOPPING GUIDELINES: 44
13.4 ANALYSIS OF PRIMARY OBJECTIVE 45
13.5 ANALYSIS OF SECONDARY OBJECTIVES 45
13.6 EVALUATION OF TOXICITY 46
13.7 CORRELATIVE SCIENCE 46

14. REFERENCES 47

15. APPENDICES 51

APPENDIX I: BRIEF PAIN INVENTORY FORM 52
APPENDIX II: PERFORMANCE STATUS CRITERIA 54
APPENDIX III: CAPP-SEQ SAMPLE COLLECTION & PROCESSING 55
APPENDIX IV: EPIC-HD CTC SAMPLE COLLECTION & PROCESSING 56
APPENDIX V: IMMUNOSEQ SAMPLE COLLECTION & PROCESSING 58
APPENDIX VI: RECTAL SWAB COLLECTION FOR MICROBIOME ANALYSIS 59
APPENDIX VII: COLOR SALIVA KIT SAMPLE COLLECTION 60

SCHEMA

Eligibility
Men with histologically confirmed oligometastatic (≤3 mets)
hormone-sensitive prostate cancer and the following:
PSADT <15 mos, tumors ≤ 5.0 cm or <250 cm^3, Age ≥18 yrs & ECOG ≤ 2

↓

Randomize 1:2
Observation (n=18) *versus* SBRT (n=36)

↓

Day 1
OBS and SBRT Arm: DCFPyL-PET/MRI or -PT/CT

↓

Day 1
OBS & SBRT Arm: PSA, LDH, CBC, Chemistry Panel (CMP), ctDNA (CAPP-seq), Rectal Swab, & Color Test**
* SBRT Arm ONLY: + CTC (Epic-HD) & PBMCs (ImmunoSEQ) *** Assays

↓

Day 1-30
Observation (OBS) *versus* **SABR to 1-3 mets**

↓

Day 90
OBS and SBRT Arm: PSA, LDH, CBC, CMP, Testosterone, ctDNA (CAPP-seq), & Rectal Swab
* SBRT Arm ONLY: + PBMCs (ImmunoSEQ)***

↓

Day 180
OBS and SBRT Arm: Bone Scan & DCFPyL-PET/MRI or -PT/CT

↓

Day 180
OBS and SBRT Arm: PSA, LDH, CBC, CMP, Testosterone, & ctDNA (CAPP-seq)
* SBRT Arm ONLY: +CTCs (Epic-HD)

↓

Patients that progress in the Observation (OBS) Arm can be crossed over off-protocol to receive SBRT.
All other patients can continue with standard of care.
* Additional Blood Correlative for SBRT Arm ONLY
** One-time Color Test will be provided for ALL enrolled patients
***First 10 patients randomized to OBS Arm will have PBMCs (ImmunoSEQ)

1. PROTOCOL SYNOPSIS

TITLE	Phase II Randomized Observation versus Stereotactic Ablative RadiatIon for OLigometastatic Prostate CancEr (ORIOLE) Trial
STUDY PHASE	Phase II
INDICATION	Men with oligometastatic disease (<3 mets) and PSADT <15 months
PRIMARY OBJECTVES	• Proportion of men who have progressed after 6 months from randomization to observation *versus* stereotactic body radiation therapy (SBRT) who have oligometastatic prostate cancer
SECONDARY OBJECTIVES	• To assess the toxicity of SBRT in patients with oligometastatic disease<br>• To determine local control at 6-months after SBRT in patients with oligometastatic disease<br>• To assess progression free survival after randomization to observation *versus* SBRT<br>• To assess ADT-free survival after randomization to observation *versus* SBRT<br>• To assess quality of life following control *versus* SBRT arm<br>• Estimate the proportion of metastatic lesions found on bone scan that are DCFPyL-PET/MRI or –PET/CT positive and vice versa.<br>• Estimate the proportion of DCFPyL-PET/MRI or –PET/CT positive sites that are positive for new or progressive metastatic disease by bone scan at 6-months from SBRT and vice versa
CORRELATIVE SCIENCE	• To enumerate circulating tumor cells (CTC) using EPIC HD-CTC platforms at baseline and day 180 from randomization.<br>• To enumerate circulating tumor DNA (ctDNA) using Cancer

	Personalized Profiling by deep sequencing (CAPP-Seq) at baseline, day 90 and day 180 from randomization for control and SBRT arms.<br>• To quantitatively sequence T-cell receptor (TCR) repertoires using peripheral blood monocytes and the ImmunoSEQ platform at baseline and day 90 from randomization.<br>• To determine frequency of germline DNA repair mutations in the oligometastatic state<br>• Descriptive statistics will be performed to correlate the metrics above with clinical outcomes.
HYPOTHESES	SBRT will reduce progression at 6-months from >80% in the control arm to <40% in the SBRT arm
STUDY DESIGN	Men with oligometastatic prostate cancer lesions will be randomized (1:2) to observation versus SBRT. The study will NOT be blinded. Within three weeks of the initial treatment planning, SBRT (1-5 fractions) will be administered.
SAMPLE SIZE BY TREATMENT GROUP	54 patients total
SUMMARY OF SUBJECT ELIGIBILITY CRITERIA	1.Metastatic bone or nodal sites ≤ 3.<br>2.PSADT <15 months<br>3.Tumor ≤ 5.0 cm or <250 cm3.<br>4.Age > 18 years.<br>5.ECOG perfomance status ≤ 2.<br>6.Histologic confirmation of malignancy (primary or metastatic tumor).
CONTROL GROUP	N/A
PROCEDURES	1. Physical exam<br>2. CT/MRI scan of Involved Site<br>3. Bone scan<br>4. Randomization<br>5. DCFPyL-PET/MRI or –PET/CT<br>6. Blood draws and Rectal Swab<br>7. Observation *versus* SBRT<br>8. Blood draws and Color Test<br>9. DCFPyL-PET/MRI or –PET/CTand bone scan of Involved Site

2. ABBREVIATIONS AND DEFINITIONS OF TERMS

ADL	Activities of daily living
AE	Adverse event
BID	Twice daily
BSA	Body surface area
CBC	Complete blood count
CI	Confidence interval
CMAX	Maximum concentration of drug
CNS	Central nervous system
CRF	Case report/Record form
CR	Complete response
CRPC	Castration-Resistant Prostate Cancer
CTC	Circulating Tumor Cell
CTCAE	Common Terminology Criteria for Adverse Events
DLT	Dose Limiting Toxicity
DSMB	Data Safety Monitoring Board
ECG	Electrocardiogram
GI	Gastrointestinal
Hgb	Hemoglobin
HIV	Human Immunodeficiency Virus
HPF	High-power field
HTN	Hypertensions
IRB	Institutional Review Board
IV	Intravenous
LLN	Lower limit of normal
MTD	Maximum tolerated dose
OS	Overall survival
PLT	Platelet
PD	Progressive diseased
PFS	Progression free survival
PR	Partial response
PSADT	PSA Doubling Time
QD	Once daily
RECIST	Response evaluation criteria in solid tumors
RR	Response rate
SABR	Stereotactic ablative radiation therapy
SAE	Serious adverse event
SBRT	Stereotactic body radiation therapy
SD	Stable disease
TTP	Time to progression
ULN	Upper limit of normal
UNK	Unknown
WBC	White blood cell

3. STUDY DESIGN AND OBJECTIVES

3.1 Study Design

Phase II non-blinded randomized study evaluating men with oligometastatic prostate cancer lesions randomized (1:2) to observation versus stereotactic body radiation therapy (SBRT).

3.2 Primary Objective

To determine the proportion of men who have progressed after 6 months from randomization to observation *versus* SBRT who have oligometastatic prostate cancer.

3.3 Secondary Objectives

To assess the toxicity of SBRT in patients with oligometastatic disease.

To determine local control at 6-months after SBRT in patients with oligometastatic disease.

To assess progression free survival (PFS) after randomization to observation *versus* SBRT.

To assess ADT-free survival after randomization to observation *versus* SBRT.

To assess quality of life following completion of SBRT.

Estimate the proportion of metastatic lesions found on bone scan/CT that are DCFPyL-PET/MRI positive and vice versa.

Estimate the proportion of DCFPyL-PET/MRI or –PET/CT positive sites that are positive for new or progressive metastatic disease by bone scan/CT at 6-months following SBRT and vice versa.

3.4 Correlative Objectives

To enumerate circulating tumor cells (CTC) using EPIC HD-CTC platforms at baseline and day 180 from randomization.

To enumerate circulating tumor DNA (ctDNA) using Cancer Personalized Profiling by deep sequencing (CAPP-Seq) at baseline, day 90 and day 180 from randomization for control and SBRT arms.

To quantitatively sequence T-cell receptor (TCR) repertoires using peripheral blood monocytes and the ImmunoSEQ platform at baseline and day 90 from randomization.

Descriptive statistics will be performed to correlate the metrics above with clinical outcomes.

4. BACKGROUND

4.1 Oligometastatic Disease

Cancer is the second leading cause of death in the United States, chiefly from an inability to control metastatic disease. Systemic therapy alone is not curative for patients with most metastatic solid tumors (1). The metastatic capacity of cancers behaves along a spectrum of disease progression, such that some tumors have spread widely before clinical detectability and others never metastasize. Contained within this spectrum, is an oligometastatic state where metastases are limited in number and location. The presence of an oligometastatic state was originally proposed by Hellman who suggested that these oligometastatic patients would benefit from effective local therapy in addition to systemic therapy (1). In agreement with this hypothesis, surgery and chemotherapy for isolated pulmonary metastases can result in long term disease-free periods (2). Additionally, some 25% of patients following resection and chemotherapy for colorectal cancer and isolated liver metastases can similarly have long-term disease free survival (3)(4)(5).

The treatment of metastases depends on multiple factors including 1) the location of the primary tumor, 2) the presence or absence of other metastatic foci, 3) the size, number and location of metastases, 4) the effectiveness of various forms of therapy (such as surgery, radiation and chemotherapy), and 5) patient's functional status. Extracranial metastatic tumors most often referred for local therapy in the form of resection include colon cancer, sarcomas, germ cell tumors, and melanoma metastatic to the lung. For the more common primary tumors such as those of breast and prostate, metastases are rarely referred for resection as chemotherapy (or hormonal manipulation) is generally considered to be the primary mode of treatment.

4.2 Stereotactic Body Radiation Therapy (SBRT) or Stereotactic Ablative Radiation Therapy (SABR)

Conventional moderate dose radiation for metastatic disease is given primarily for palliation. Recent advancements in radiation delivery now make it possible to image and treat precisely within any anatomical region of the body (6, 7). As a result, the capacity to deliver tumor killing radiation doses in a single or few (1-5) outpatient radiation treatments is now possible (8-12). In addition, by minimizing the irradiation of surrounding healthy tissue, it should also be possible to decrease the rate of complications. Intracranial stereotactic body radiation therapy (SBRT) [also known has stereotactic ablative radiation therapy (SABR)] has been shown to be a highly effective treatment for brain metastases (13). Data suggests that selective small extracranial tumors (either primary or metastatic tumors) may be effectively controlled by similar focal high-dose SBRT/SABR. There is an increasing experience with extracranial SBRT as effective local therapy for metastatic lesions. Local control in excess of 75% has been reported for metastatic tumors of the spine, lung and liver, which is significantly higher than standard conventional moderate dose radiation (9, 11,

12, 14)(15-27). Toxicity has been minimal in multiple U.S., European and Japanese trials of extracranial stereotactic radiotherapy to the lung, liver, spine, pelvis and abdomen despite the use of very high biological equivalent doses for patients with both organ confined and metastatic cancer.

4.3 Rationale for Use of SBRT/SABR in Oligometastatic Disease

Historically, aggressive local therapy of metastases has not been fruitful secondary to further progression from microscopic metastatic deposits. Chemotherapy and even molecular-targeted agents rarely eradicate macroscopic metastases permanently. However, as systemic treatments for microscopic metastatic disease have improved the importance of local therapy in metastatic disease has been re-examined. It is now recognized that some patients with "oligo," or few sites of metastases, may have isolated sites of metastases that can be potentially eradicated with aggressive local therapy. The term "oligometastases" was coined to refer to this stage of distant metastases. Typically, the entire burden of disease can be recognized as a finite number of discrete lesions. Although there is no strict definition of oligometastases, it is commonly interpreted to be no more than 5 or 6 metastatic sites.

Milano et al. (9) from the University of Rochester have also shown that the net tumor burden for patients with 5 or fewer sites of macrometastatic disease, treated on 2 prospective SBRT protocols, was an independent predictor of overall outcome. Whether SBRT, as an adjunct to systemic therapy, to selected macroscopic metastases can influence overall survival by keeping the burden of disease below such a "lethal threshold" is being investigated.

Colorectal carcinoma metastatic to the liver, selected modern resection series have yielded 5-year survival rates of approximately 50% showing that improved systemic treatments and local therapy has the potential to cure "oligo" or isolated liver metastases (5, 9, 28-31). The benefit of local therapy in non-colorectal liver metastases is less clearly defined, but long-term survival has been reported after the resection of liver metastases from sarcoma, breast cancer, and other tumor sites (32).

Goodman et al. (15) reported a phase I dose-escalation single-fraction trial for patients with liver metastases or intrahepatic cholangiocarcinoma. Doses were escalated in 4-Gy cohorts from 18 Gy up to 30 Gy in 1 fraction. Twenty-six patients with 40 lesions were treated. There was no dose-limiting toxicity. The median follow-up was 17 months, and this corresponded to a 12-month local control rate of 77%. The 2-year actuarial survival rate was 50.4%.

4.4 Rationale for SBRT/SABR for Bone Oligometastases in Prostate Cancer

Major advancements in radiation treatment planning and delivery have resulted in resurgence in the use of radiation therapy (RT) as a treatment for bone metastases. SBRT/SABR is defined as highly focused, stereotactic localized,

high-dose RT delivered in a hypofractionated course. In selected patients, very high local control rates have been observed, with minimal toxicity. Bone metastases represent the major metastatic site (>90%) in men with rising PSA following primary treatment for their prostate cancer.

The primary management of metastatic prostate cancer is systemic therapy in the form of androgen deprivation therapy (ADT). Many men can remain on ADT treatment for years before progression or failure of ADT. However, similar to chemotherapy for other metastatic malignancies, ADT and even newer androgen receptor signaling inhibitory agents rarely eradicate metastatic disease permanently. In addition, ADT has been shown conclusively to adversely affect patient quality of life. Thus, even the ability to defer ADT initiation in men with oligometastatic prostate cancer represents a considerable clinical advance.

On the basis of this emerging clinical evidence and because SBRT for bone metastases is known to be safe, we propose a phase II study of SBRT in patients with oligometastatic prostate cancer. Abundant experience with SBRT for bone metastases provides useful safety information for our trial. We are also building off of our Phase II SBRT trial in diverse histologies here at Johns Hopkins - J12137. This trial has almost completed accural to the target of 42 patients, but we have already finished accuring the 20 prostate cancer patient limit. Although very preliminary we have seen only low G1-G2 toxicites and no local failures to date in our prostate cancer patients. Thus the proposed study represents an informed estimate based on current knowledge of SBRT doses and those administered in currently approved image-guided protocols including our own JHU protocol (brain, base of skull, cervico-thoracic spine, pancreas and liver).

In general metastatic disease carries an extremely high mortality rate. Current therapies provide only partial palliation of symptoms and mild to moderate prolongation of survival. Patients are rarely cured of this disease; consequently, better treatment is clearly needed. The proposed treatment represents a logical extension of the current state-of-the-art radiation therapy. It has the potential to translate into more effective palliation and longer patient survival free of intiation of systemic treatment.

4.5 Rationale for Prostate specific membrane antigen (PSMA) Functional Imaging to Help Refine Selection of Bone Lesions to Target with SBRT/SABR in Oligometastatic Prostate Cancer

Conventional imaging modalities, *i.e.,* bone scintigraphy, CT and MR imaging, are currently used to detect metastatic prostate cancer for staging (4). Positron emission tomography (PET) imaging, particularly [^{18}F]fluorodeoxyglucose positron emission tomography ([^{18}F]-FDG PET), has gained an important role in the clinical management of cancer patients, more notably for staging and assessment of response to therapy (32, 33). However, studies employing [18F]-

FDG PET have demonstrated low uptake in prostate cancer except for advanced metastatic disease (34-37). A number of novel PET radiotracer are being investigated for use in prostate cancer but have yielded mixed results and have yet to gain widespread clinical use (38, 39). [11C]Choline is the most widely studied PET radiotracer for prostate cancer detection and has demonstrated the ability to detect lymph node and bone metastases (40-42). [11C]Acetate is another emerging radiotracer, which has been evaluated in a limited number of studies and appears to demonstrates comparable uptake in comparison to [11C]Choline for detection of prostate tumor and metastases (43). One limitation of these radiotracers include nonspecific uptake at sites of inflammation. 18F-Fluoride-PET (NaF-PET) for identifying bone metastases has proven very sensitive but is unable to differentiate between viable metastatic prostate tumor from chronic reactive bone changes (44, 45). Other promising radiopharmaceuticals for prostate cancer include anti-1-amino-3-18F-fluorocyclobutane-1-carboxylic acid (18F-FACBC) and 18F-fluorodihydrotestosterone (18F-FDHT), which are also actively undergoing clinical evaluation in a variety of settings (46-48). Further work needs to be done to compare the merits of [11C]Choline and other emerging PET radiotracers in the detection and management of prostate cancer (49).

Prostate specific membrane antigen (PSMA is a promising well-characterized biomarker specific for prostate cancer which has also been associated with prostate tumor aggressiveness. Histologic studies have associated high PSMA expression with metastatic spread (50-52), androgen independence (53), and expression levels have be found to be predictive of prostate cancer progression (54, 55). However, previous attempts to image PSMA by single-photon-emission computed tomography (SPECT) (Prostacint™) demonstrated poor performance due to inherent limited antibody and imaging characteristics (poor tumor penetration, slow blood pool clearance, low SPECT resolution) (56, 57).

[^{18}F]DCFBC (N-[N-[(S)-1,3-Dicarboxypropyl]Carbamoyl]-4-[18F]Fluorobenzyl-L-Cysteine) (DCFBC) is a promising clinically practical small molecule PET imaging agent specific for prostate cancer with superior pharmacodynamic and pharmacokinetic characteristics than existing prostate cancer imaging agents. This is a small molecule urea-based analog inhibitor of PSMA radiolabeled with a PET radiotracer fluorine-18 which was rationally designed based on knowledge of the crystal structure of PSMA (58, 59). DCFBC is a high affinity inhibitory ligand for the prostate specific membrane antigen (PSMA). The relative affinity of DCFBC for PSMA was determined by evaluating ability of DCFBC to inhibit the N-acetylaspartylglutamate (NAAG) peptidase activity of PSMA using a previously developed NAAG peptidase assay. The IC50 value for the inhibitory capacity of DCFBC for PSMA was 13.9 nM, as determined by a NAAG peptidase inhibition assay, in keeping with other compounds of this class. Our proposed work is innovative because DCFBC PET imaging will optimize functional PSMA prostate cancer imaging by improving the following factors: (1) as a small molecule it allows for higher tumor penetration and rapid blood clearance allowing for

increased tumor to background ratio, (2) it targets the more accessible external binding domain of PSMA, and (3) PET imaging allows for quantitative high resolution images, and (4) clinically practical labeling with ^{18}F, with a 2-hour half-life. PSMA-specific uptake of this radiotracer have been validated in PET imaging of pre-clinical human prostate cancer xenograft models which verify high selective uptake in PSMA expressing prostate tumors but no uptake in prostate tumors not expressing PSMA (60, 61). Small animal PET imaging of DCFBC in subcutaneous prostate cancer bearing mice demonstrated specific uptake in PSMA expressing PC-3 PIP tumors, achieving a maximum target to background (muscle) ratio of 20:1 at 120 min after injection. The time-activity curve demonstrates that DCFBC has achieved equilibrium by 120 minutes and has begun to decrease in concentration at the target site, with washout from target sites slower than non-target sites.

Preliminary results of a recently completed Phase I first-in-man biodistribution and dosimetry study of DCFBC in five patients with advanced metastatic disease demonstrated high radiotracer uptake in both nodal and bone metastatic sites, minimal radiotracer metabolism, and favorable biodistribution (62). Of 32 total sites of DCFBC uptake, 21 were concordant with prostate cancer metastases as determined by conventional imaging modalities (CIM) (CT, bone scan) at 21 sites and the other 11 sites of DCFBC uptake not detected by CIM were unconfirmed but considered suspicious for metastases. Seven bone findings thought to be benign fracture or stable changes on bone scan were negative for DCFBC uptake. Radiotracer administration was found to be safe with no severe adverse events. Tumor-to-background ratio for metastatic detection was highest at 2 hours post-radiotracer injection. A 2-hour post-injection DCFBC PET/CT with anterior MIP image demonstrating multiple pelvic bone metastases and sagittal PET, CT, and fused PET/CT images demonstrating T12 and L4 bone metastases (SUVmax 4.7 and 3.4, respectively). A 2-hour post-injection DCFBC transaxial PET/CT in another patient with prominent high level DCFBC uptake in a large right external iliac (SUVmax 11.6) and smaller left common iliac (SUVmax 5.3) pelvic nodal metastases. Dosimetry studies demonstrates the highest mean absorbed dose per unit administered activity (µGy/MBq) was to the bladder wall (32.4) and the mean effective dose (ED ± StdDev) was 19.9 ± 1.34 µSv/MBq, with dose estimates for our DCFPyL PET radiopharmaceutical that are comparable to those of other PET radiopharmaceuticals such as ^{18}F-fluorodeoxyglucose. Although further studies are needed for validation, our findings demonstrate the potential of DCFBC-PET imaging as a new positron-emitting imaging agent for detection of metastatic prostate cancer.

2-(3-{1-carboxy-5-[(6-[^{18}F]fluoro-pyridine-3-carbonyl)-amino]-pentyl}-ureido)-pentanedioic acid, DCFPyL, is a second generation compound that includes the pyridine moiety intended to improve pharmacokinetics through altered lipophilicity and potential pH dependent intra-tumoral sequestration. Extensive study of both DCFBC and DCFPyL in pre-clinical models has been performed and published, attesting to their suitability for clinical translation – including with respect to

dosimetry. While the first generation compound (DCFBC) provided isogenic PSMA+ human PC3 PIP tumor to PSMA- PC3 flu tumor ratios of 20:1 at 3 h post-injection, DCFPyL demonstrated ratios on the order of 400:1. Neither compound demonstrates appreciable de-fluorination *in vivo* and PSMA+ PIP to bone ratios are sufficient to enable imaging of bone metastases, as demonstrated below for DCFBC. After synthesis of ~100 compounds, including those with several new scaffolds designed to target PSMA, DCFPyL emerged as the best *in vivo* (63). On the basis of this promising clinical evidence with DCFBC-PET and the superiority of DCFPyL pre-clinically and because bone scintigraphy and NaF-PET are non-specific, we propose as a secondary objective evaluating the performance of DCFPyL-PET versus the conventional bone scan as a means to identify bone lesions for SBRT treatment in oligometastatic prostate cancer.

4.6 Rationale for Correlative Science

Novel Models of Metastasis and Circulating Tumor Cell (CTCs). Older models of metastasis portray the unidirectional flow of circulating tumor cells (CTCs) leaving the primary tumor and seeding a metastasis at a distant site (64). However, recent preclinical data using diverse experimental models of breast cancer, colon cancer and melanoma suggest metastasis is a multidirectional process where CTCs seed both distant sites as well as the original primary tumor – a process termed "self-seeding"(65, 66). Proponents of self-seeding have posited that CTC self-seeding of established macroscopic tumor sites likely requires less or no adaptation of CTCs to the recipient microenvironment in comparison to the colonization of CTCs to a distant and foreign site. In addition, self-seeding CTCs have already undergone selection for movement into and out of the circulation as well as resistance to anoiksis. Pre-clinical data have shown that self-seeding CTCs home back and extravasate into the primary in reaction to signals from the recipient primary tumors cells and tumor stroma. These self-seeding CTCs appear to be the most aggressive fraction of the CTC population (65). This feed forward loop of increasingly more aggressive cancer cells interacting with the tumor stroma results in the release of signals that foster tumor growth, angiogenesis, immune evasion, and ultimately macroscopic metastases.

Interestingly, genomic lineage tracing data of metastases from a rapid autopsy series of men who died of metastatic prostate cancer suggest macroscopic metastases represent *communal sanctuaries* that are composed of prostate cancer cells from many other metastatic sites throughout the body of patients (67). These *communal sanctuaries* are favorable niches that allow prostate cancer cells the ability to gain competence for the development of future macroscopic metastases. These human data are consistent with the preclinical concept of "self-seeding" or a multidirectional flow of CTCs. If these provocative data hold true, then SBRT/SABR to all macroscopic metastases in oligometastatic patients may eliminate these *sanctuaries* and alter the natural history of metastatic patients. Following change in CTC numbers and biology

following SABR may allow us to interrogate this hypothesis. Interventions that effectively target these macroscopic metastatic sanctuaries, such as SBRT/SABR, in combination with immune mediated therapies appear poised to arrest self-seeding and subsequent maturation of macroscopic metastases.

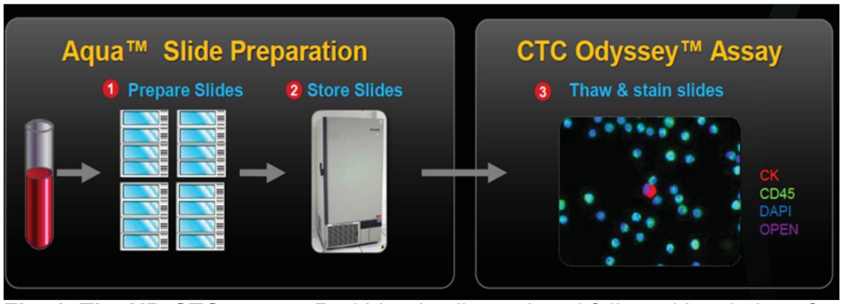

Fig. 1. The HD-CTC assay. Red blood cells are lysed followed by plating of nucleated cells on custom made cell-adhesion glass slides. Slides are stained for cytokeratin (CK), CD45 and DAPI, then CTCs are identified among leukocytes using computerized high resolution immunofluorescence imaging.

EPIC High-Definition analysis of single CTCs. The High Definition-CTC (HD-CTC) method can be used for the longitudinal CTC enumeration and to assess for AR expression (68). The assay employs as little as 1 mL of blood and is an unbiased protocol to distinguish CTCs among the surrounding leukocytes based on their cytokeratin positive (CK+) phenotype by using a high resolution immunofluorescence imaging. All cells are captured, and AR- cells can be evaluated. In addition, the HD-CTC technology preserves the cell morphology in such a way that enables the morphometric and the indirect quantification of AR and CK protein expression levels for all the CTCs identified in the blood sample (Fig. 1). Recently, the HD-CTC method has been used to examine expression of immune checkpoint molecules relevant to our proposal such as programmed cell death-1 (PD-1) on CTCs (data not shown). The HD-CTC assay was technically validated with cell line spiking experiments to reach an R2=0.9997 on linearity testing as previously reported. These experiments were performed using SK-BR-3 cell lines and 0 to 3x102 cells per mL of normal donor control blood. The coefficient of variation is 16% and inter-processor correlation is R2 = 0.979. Sample preparation process adhered to standard operating procedures for patient samples through a bar coded system for all consumables and instrumentation. All off-the-shelf instrumentation was calibrated according to analytical validation protocols established during commissioning.

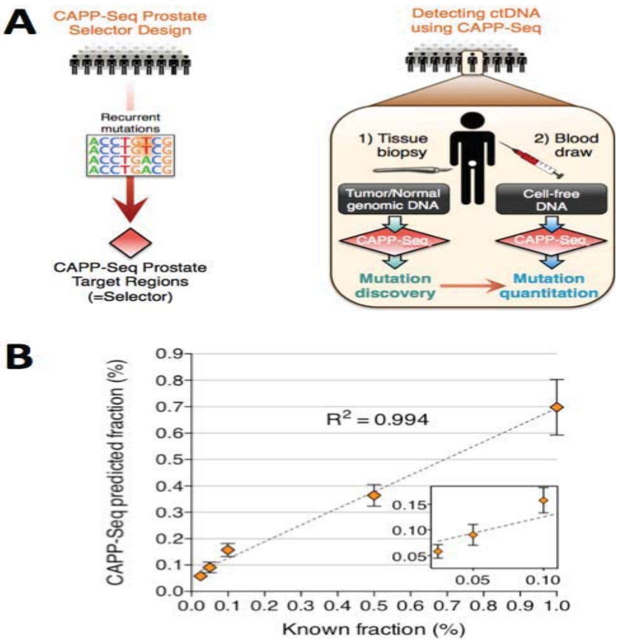

Fig. 2. CAPP-Seq ctDNA detection. (**A**) Analysis of exome sequencing from many tumors is used to select mutated genomic regions for a custom library of biotinylated oligonucleotides that are used for hybrid capture selection of tumor and germline genomic DNA and ctDNA. (**B**) Dilution series analysis of expected versus observed frequencies of mutant alleles, assessed by spiking fragmented HCC78 DNA into control cell free DNA.

Cancer personalized profiling by deep sequencing (CAPP-Seq) to

detect circulating tumor DNA. Circulating tumor DNA (ctDNA) is a promising biomarker for non-invasive assessment of cancer burden, but existing ctDNA detection methods have either insufficient sensitivity and/or lack broad clinical applicability. Dr. Max Diehn's laboratory recently published Cancer Personalized Profiling by deep sequencing (CAPP-Seq) (69), an economical and ultrasensitive method for quantifying ctDNA. CAPP-Seq was initially implemented for lung cancer with a design covering multiple classes of somatic alterations that identified mutations in >95% of tumors. We detected ctDNA in 100% of patients with stage II–IV lung cancer and in 50% of patients with stage I, with 96% specificity for mutant allele fractions down to ~0.02%. Levels of ctDNA were highly correlated with tumor volume and distinguished between residual disease and treatment-related imaging changes, and measurement of ctDNA levels allowed for earlier response assessment than radiographic approaches from 1.5 mL of blood (**Fig. 2**). We have designed a prostate cancer-specific CAPP-Seq selector that identifies at least 1 mutation in $\geq$95% of prostate tumors with a median of ~4 mutations per tumor. We will apply CAPP-Seq to detect and monitor prostate cancer response to SBRT/SABR.

ImmunoSEQ allows Unprecedented Profiling of the Anti-Tumor Immune Response. There are now an established body of pre-clinical and emerging clinical literature demonstrating that SABR can profoundly modify anti-tumor immune responses. Radiation induced activation of antigen presenting cells has been demonstrated in animal models to enhance tumor antigen cross-presentation in the draining lymph node and result in activation and proliferation of tumor specific cytotoxic T-cells (78). The cellular adaptive immune system generates a remarkable breadth of diversity in antigen-specific TCRs by combinatorial recombination of gene segments in lymphocytes. The TCR is composed of two peptide chains, encoded by the TCRA and TCRB or TCRG and TCRD genes, respectively. There are thus two types of T-cell receptors, αβ and γδ, that differ by the TCR heterodimer type and immune function. The antigenic specificity of T-lymphocytes is in large part determined by the amino acid sequence in the hypervariable complementarity-determining region 3 (CDR3) regions of the T-cell receptors. Because of the potential diversity of receptors (a healthy adult has approximately 10 million different TCRB chains contained within their 10^{12} circulating T-cells (70)) it is highly improbable to randomly converge on the same TCRB nucleotide CDR3 sequence, effectively making each CDR3 sequence a unique tag for a T-cell clone. Adaptive Biotechnologies' ImmunoSEQ assay, a multiplex PCR-based method that amplifies rearranged TCR CDR3 sequences and exploits the capacity of high throughout sequencing technology characterizes tens of thousands of TCRB CDR3 chains simultaneously. Thus, the assay captures the full TCR repertoire including specific individual clones. The ImmunoSEQ assay provides a novel method to identify and track the presence and frequency of common and rare clones, in the context of the total adaptive immune system. Recently, ImmunoSEQ showed profound evolution and diversification of the TCR repertoire of men with mCRPC treated with the immune stimulatory agent

ipilimumab (71). Improved clinical outcomes were associated with less T-cell clonotype loss, consistent with the maintenance of high-frequency TCR clonotypes during treatment reported by ImmunoSEQ. These clones may have represented the presence of preexisting high-avidity T cells that may be relevant in the antitumor response.

4.7 Rational for Color Genomics

The evolution of human genome sequencing has enabled the assessment of genetic anomalies in routine clinical practice. According to a recent multicenter study in the *New England Journal of Medicine*, it was reported that inherited mutations in DNA-repair genes were much higher than expected in men with lethal metastatic castrate resistant prostate cancer (79). The study isolated germline DNA of 692 men with metastatic prostate cancer (unselected for family history of cancer or age at diagnosis), and identified 84 deleterious germline DNA-repair gene mutations in 82 of those men (79). This was substantially higher than in men with non-metastatic hormone sensitive localized disease. The prevalence of germline DNA-repair mutations in the oligometastatic hormone sensitive or castrate-resistant state is unknown.

In April 2015, Color launched a test with 19 genes in which germline mutations have been associated with an elevated risk of cancer. In the hereditary cancer genetic test, Color uses a sequencing platform to analyze the risk of developing hereditary cancer due to inheritance of a pathogenic mutation in 30 cancer predisposition genes (81). The Color Genomics panel, covers 12 of 16 genes (BRCA1, BRCA2, MSH2, MSH6, PMS2, CHECK2, ATM, NBN, BRIP1, RAD51C, and RAD51D) that account for 92% of the germline mutations reported in Pritchard et al NEJM. Thus, inherited prostate cancer genomic data provided by Color can further allow investigators to personalize screening and target specified effective therapies for each patient disease (80).

5. PARTICIPANT SELECTION AND ENROLLMENT PROCEDURES

All patients will be eligible to receive systemic therapy alone at the time of clinical or radiographic disease progression.

5.1 Inclusion Criteria

5.1.1 Patient must have at least one and up to three asypmtomatic metastatic tumor(s) of the bone or soft tissue develop within the past 6-months that are ≤ 5.0 cm or <250 cm^3

5.1.2 Patient must have had their primary tumor treated with surgery and/or radiation.

5.1.3 Histologic confirmation of malignancy (primary or metastatic tumor).

5.1.4 PSADT <15 months. PSA doubling time (PSADT) will be calculated using as many PSA values that are available from time of relapse (PSA > 0.2). To calculate PSADT, the Memorial Sloan Kettering Cancer Center Prostate Cancer Prediction Tool will be used. It can be found at the following web site: https://www.mskcc.org/nomograms/prostate/psa-doubling-time.

5.1.5 Patient may have had prior systemic therapy and/or ADT associated with treatment of their primary prostate cancer. Patient may have had ADT associated with salvage radiation therapy (to the primary prostate cancer or pelvis is allowed).

5.1.6 PSA ≥ 0.5 but ≤50.

5.1.7 Testosterone ≥ 125 ng/dL.

5.1.8 Patient must be ≥ 18 years of age.

5.1.9 Patient must have a life expectancy ≥ 12 months.

5.1.10 Patient must have an ECOG performance status ≤ 2.

5.1.11 Patient must have normal organ and marrow function as defined as:

Leukocytes	≥2,000/µL
Absolute Neutrophil Count	≥1,000/µL
Platelets	≥50,000/µL

5.1.12 Patient must have the ability to understand and the willingness to sign a written informed consent document.

5.2 Exclusion Criteria

5.2.1 No more than 3 years of ADT is allowed, with the most recent ADT treatment having occurred greater than 6 months prior to enrollment.

5.2.2 DCFPyL-PET/MRI or DCFPyL-PET/CT scan within the past 6 months with results that demonstrate more disease lesions than baseline CT/Bone Scan

5.2.3 Castration-resistant prostate cancer (CRPC).

5.2.4 Spinal cord compression or impending spinal cord compression.

5.2.5 Suspected pulmonary and/or liver metastases (greater ≥10 mm in largest axis).

5.2.6 Patient receiving any other investigational agents.

5.2.7 Patient is participating in a concurrent treatment protocol.

5.2.8 Serum creatinine > 3 times the upper limit of normal.

5.2.9 Total bilirubin > 3 times the upper limit of normal.

5.2.10 Liver Transaminases > 5-times the upper limit of normal.

5.2.11 Unable to lie flat during or tolerate PET/MRI, PET/CT or SBRT.

5.2.12 Prior salvage treatment to the primary prostate cancer or pelvis is allowed.

5.2.13 Refusal to sign informed consent.

6. TREATMENT PLAN

6.1 Randomization

Eligibility work-up will include a complete blood count, serum chemistries, PSA, and radiographic studies (of involved sites) and bone scan. Once a signed informed consent has been obtained and after confirming patient eligibility, the lead data coordinator will assign a unique Study ID Number.

Treatment must not commence until the patient has received his identification number.

Stratification:
Subjects who meet eligibility criteria and qualify for enrollment will be stratified according to the following:

1) Initial treatment with surgery vs. radiation therapy
2) Prior hormonal therapy vs. no prior hormonal therapy

3) PSADT <6 months vs. 6-14.9 months

Process for Randomization:
The research team will utilize an interactive web response system (IWRS) to obtain the patient's randomization assignment. Randomization will be 1:2 for observation: SBRT arms and be stratified as above. Minimization approach will be applied to ensure balanced assignment to each treatment arm. The **ON STUDY date for protocol entry** will be the day that the study subject is randomized. Within 30 days from the date of randomization, is considered DAY 1 of SBRT and/or Observation Arm.

6.2 Diagnostic Procedures

A bone scan and CT (and/or MRI of questionable sites) must be avilaible or will be obtained within 3-months of randomization for confirmation of oligometastatses and a separate DCFPyL-PET/MRI or -PET/CT scan will be performed for protocol purposes as below. A CT- and/or MRI-simulation scan will be performed for tumor localization and radiation planning using rigid immobilization appropriate for stereotactic treatment.

6.3 Therapeutic Procedures

Upon confirmation of eligibility and enrollment in the study, the following will be confirmed or completed:

1) Demographics review, medical history and clinical exam
2) Review of concurrent medications
3) Vital signs, height and weight
4) PSA, Testosterone, CBC, Chemistry Panel, LDH, CAPP-seq**, and ImmunoSEQ**
5) **Additional correlative blood draws: Epic HD-CTC only <u>for men randomized to the SBRT arm</u>**
6) CT and/or MRI scan of the involved site(s) (if not previously conducted within 3 month of enrollment).
7) Bone scan (if not previously conducted within 3 month of enrollment).
8) **DCFPyL-PET/MR or -PET/CT Diagnostic Imaging Protocol.** Our collaborating center at the NIH Clinical Center has a PET/MRI available and will proceed with these exams, while at the Johns Hopkins campus will be proceed with PET/CT. The subjects accrued at the NIH will be scanned on a Siemens Biograph mMR PET/MR scanner, a simultaneous PET and MR imaging system. The system is operated by the radiology department of the National Institutes of Health clinical center located on the NIH campus in Bethesda MD. The Johns Hopkins campus has Discovery DRX PET/CT scanner (GE Healthcare) imaging systems.

Patient Preparation.
i. Patient has to be NPO for 4-6 hrs.
ii. When the patient arrivals in the clinic they are explained the procedure by the Nuclear Medicine Staff and all questions are answered prior the start of the procedure.
iii. IV access is obtained and verified that it is patent with a saline flush prior to giving the F-18 DCFPyL.
iv. A dose of 9-10 mCi F-18 DCFPyL is injected through the IV and followed by at least 10 ml of saline to flush the IV line of the remaining dose.
v. Intravenous fluid (5% dextrose + 0.45% normal saline) will be delivered at a low flow rate during the duration of the study (maximum 2 litters).
vi. The patient is asked to void prior to the scan 15-20 minutes before the scan. After the patient is finished voiding, they are positioned on the scanner bed with the head placed first and arms down to their side.
vii. A CT or (MR) localizer scan is taken from the top of the head to mid-thigh to properly frame the subjects' skull vertex through mid-thigh in the CT (or MR) scanner's field of view. It is expected to require 7 to 9 bed positions.
viii. At the 1-hour post injection time (50-70 minutes), the PET/CT or PET/MR imaging sequence begins.
ix. The PET system of the CT (or MR) scanner will collect data for each bed position required to scan the subject from the skull vertex to mid-thigh. The scan time for each bed position will be on the order of 7 to 8 minutes depending on the details setup of the MR imaging sequences. The PET images will be reconstructed using a 3D iterative reconstruction algorithm, with 3 iterations and 24 subsets. Detector pair normalization, random scatter and attenuation corrections will be applied during the image reconstruction process. No image post filtering will be applied.
x. Simultaneous with the PET acquisition the following MR imaging sequences will be collected.

a. A T1 weighted gradient echo TR=200ms, TE =2.3ms, slice thickness=5mm, matrix =376x116, FOV 47cm
b. DWI TR=6000ms, TE=79ms, slice thickness=5mm, matrix=128x128, b=0,1000,2000,FOV 47cm
c STIR TR=12000, TE=110, TI=200ms, slice thickness=5mm, matrix =376x116, FOV 47cm

The study PI, co-I's or study coordinator will contact the patient around 7 days (3 - 10 days) after the PET/CT or PET/MRI study to inquire about delayed side effects. If there are suspected problems the patient will be asked to return for a clinic visit for a follow-up clinic visit.

DCFPyL-PET/MRI or -PET/CT images will be evaluated and compared to bone scan. However, additional sites(s) of suspected metastatic disease detected by DCFPyL-PET/MRI or -PET/CT will not be considered for treatment by SBRT/SABR or undergo further required evaluation. The results of the DCFPyL-

PET/MRI or –PET/CT will not be made available to the treating physicians until after the trial is completed and these results will not be made available to the patient.

9) SBRT/SABR treatment planning

CT- and/or MRI-simulation will then be performed with fabrication of a radiation therapy immobilization device (such as the Alpha Cradle) which will be custom made for each patient. The treating radiation oncologist will identify the location of the tumor. Gross tumor volume (GTV) delineation will be performed with a diagnostic radiologist on sequential axial computed tomography images. A radiosurgical treatment plan will be developed based on tumor geometry and location. The clinical tumor volume (CTV) will equal the GTV. The dose will be prescribed to the minimal isodose line that completely covers the planning target volume (PTV) PTV (=CTV plus a 5 mm margin). Adjacent normal structures including but not limited to the heart, esophagus, aorta, spinal cord, kidneys, rectum, bowel, liver, and stomach within 5 cm of the CTV will be identified for the purpose of limiting incidental radiation to these structures.

In addition, prior to treatment delivery, a four-dimensional cone beam CT study will be performed on individual patients to assess respiration in these patients and to determine tumor targeting accuracy for those tumors that may be subject to respiratory motion such as those in the bones of the thorax. If tumor motion is greater than 5 mm, PTV will be expanded to account for respiration.

SBRT/SABR will be delivered in 1 to 5 fractions, and the dose and fractionation schedule will depend on the size and location of the lesion and the surrounding normal tissue constraints in accordance with AAPM Task Group 101 recommendations. Typical doses include 16 – 24 Gy in 1 fraction, 48 – 50 Gy in 4 fractions, and 50 – 60 Gy in 5 fractions. For example, isolated osseous lesions will be treated in a single fraction, lesions close to the lung and liver lesions will be treated in 3 to 5 fractions depending on their size (5 fractions for ≥ 3 cm or central tumors in close proximity to the mediastinum), and bone lesions will be treated in 5 fractions if small-bowel constrains fewer doses.

Within three weeks of the initial treatment planning imaging study, SBRT/SABR will be administered using image-guidance. An Alpha Cradle (or equivalent immobilization device) will be used to minimize movement of the chest, spine, and abdomen during treatment. During treatment, real time cone beam CT images of the patient's body site of interest will be obtained. Cone beam CT scan will be obtained immediately prior to treatment and will be repeated until the treatment shift, required to align the CT planning scan and the cone beam CT scan performed on the day of treatment cone beam CT, is within tolerance for the body site.

Patients will be evaluated for adverse events/toxicities during their treatment.

6.3.1 The dose limits for surrounding critical structures are as follows:

Spinal Cord: maximal allowable dose should be = 800 cGy in 1 fraction

Lung: 2/3 of the lung volume should be kept under 500 cGy.

Heart: 50 % of the heart volume should be kept under 1000 cGy.

Esophagus: 50 % of the esophagus volume should be kept under 1000 cGy and no single point dose in the esophagus should exceed 2000 cGy.

Brachial Plexus: maximal allowable point dose = 1000 cGy

Liver: One third of the uninvolved liver or approximately 700 cc <15 Gy.

Kidneys: 75% of volume of each kidney <5 Gy.

Small Bowel: <5% of bowel limited to <20 Gy.

6.4 Follow-Up Procedures

6.4.1 Blood Draws

Subsequent to randomization, patients will be followed clinically and radiographically. A detailed medical and physical examination with blood draws for PSA, LDH, CBC w/Diff, Serum Chemistry panel (CMP), ctDNA (CAPP-seq), and testosterone will be performed within the first month, month 3 and 6 (from date of randomization) for all patients. Immune cell profiling (ImmunoSEQ) will be performed within the first month and at 3 months from randomization for all men on the SBRT arm and the first 10 patients randomized to the observartion arm. The patients randomized to the SBRT arm will also have blood draws for CTCs (EPIC HD-CTC) correlative studies at the start and end of the study. In clinic visits are not required otherwise, unless indicated by laboratory tests.

Correlative science blood draws will be performed at Day 1 (within 30 days of randomization), Day 90 from randomization, and Day 180 from randomization. Details for these blood draws are contained within the APPENDIX III-V.

Briefly:

CAPP–Seq: 30-ml whole blood in purple top (EDTA) tubes (see APPENDIX III for details). The blood is stored in a freezer, then shipped in batches as per APPENDIX III. Baseline, day 90 and day 180 from randomization for both arms.

EPIC HD-CTC: 4-10 ml Streck Cell-Free DNA BCT tubes (see APPENDIX IV for details). Whole blood is shipped out the same day as it is drawn as per APPENDIX IV. Baseline and day 180 from randomization for SBRT arm only.

ImmunoSEQ: 10-ml whole blood in purple top (EDTA) tubes (see APPENDIX V for details). The patient samples are stored in a 80 Degree Celsius freezer and shipped in batches as per APPENDIX V. Baseline and day 90 from randomization for all men on the SBRT arm and the first 10 patients randomized to the observation arm.

6.4.2 Imaging

Bone scan and DCFPyL-PET/MRI or –PET/CT of metastatic site(s) will be at 6 months for all patients, unless indicated more frequently by clinical or laboratory findings. The results of the DCFPyL-PET/MRI or –PET/CT will not be made available to the treating physicians until after the trial is completed and these results will not be made available to the patient.

The CT portion of the DCFPyL-PET/MRI or –PET/CT scans at 6 months will be used to determine radiographic response based on RECIST 1.1 criteria for soft tissue lesions.

6.4.3 Rectal Swab

The collected rectal swab (see APPENDIX VI for details) will provide researchers with a tool to analyze the gut microbiome of patients in an effort to investigate potential correlations between the gut microbiome and response to cancer therapies. Consented patients will be asked to self-collect the rectal swab during an office visit by inserting a cotton tipped application approximately 3 cm (about 1 inch) into the anal canal and rotating it. The swab can then be returned to the provided tube. Rectal swabs will be collected at enrollment within 1-mos of randomization but prior to SBRT and at the 90 day time point.

6.4.4 Rectal Swab Processing

The collected swabs will be immediately stored at 4° C to prevent bacteria growth before being moved to -20/-80° C. Collected swabs will be processed for DNA extraction and eventual next generation sequencing to provide a genomic profile of the bacteria present in the gut microbiome of patients.

6.4.5 Color Genomic Testing

Consented patients will be asked to provide a saliva sample (tube collection) during an office visit or at home, via a Color kit (see APPENDIX VII for details). **This collection will be performed on all enrolled patients, at any time point during study or after completion of study (F/U), regardless of their randomization arm.** The Color kit will then be activated online and dropped in the mail. Color will then analyze the genes from the patient salvia sample. Once the patient's results are ready, an email will be sent by Color prompting the PI to log into his established Color Provider Platform account and view the results. Color will also email the patients with instructions to create their own account, allowing them access to sign into Color's website and view their results (once the PI has released the patient's results). The patient will be allowed to make an appointment with one of the Color's genetic counselors at no cost. Further, Color will also keep the patient updated if any information related to their results changes.

6.5 Duration of Therapy

Within three weeks of the initial treatment planning imaging study, SBRT will be administered in a 1-5 fractions to each treated site (within 60 days from randomization) of the study and coincides with delivery of fraction 1 for the SABR experimental arm and beginning of the observations period for me on the observation arm.

6.6 Duration of Study Accrual

A trial with a similar patient population took 2.5 years to screen 77 patients (72). Thus we anticipate this study will last 2-2.5 years.

6.7 Duration of Follow-Up

Patients will be followed for 180 days, after Day one.

6.8 Criteria for Patient Removal

Diagnosis of >3 bone and/or soft tissue metastases on entry imaging studies before SBRT/SABR. PSA >50 ng/ml before SBRT. Progression as defined in section 8.6.2.

6.9 Alternatives

The study has been designed to minimize potential risks to participants. First, this population of asypmtomatic oligometastatic men is heavily pre-selected based on institutional data indicating that similar men without metastases will progress in 6-months based on rising serum PSAs (72-74). Secondly, the level of clinical-radiographic interrogation will allow the control arm to be safely observed so that standard of care treatments can be initiated within a typical interval between clinic visits (72-74). Lastly, the SBRT dose has been shown to be safe in previous SBRT trials. Risks to confidentiality will be minimized by having access to study records avaialble only to the investigators with the exception of the standard clinical records (lab values, dictations, operative notes, etc).

Standard therapies for metastatic prostate disease include radiotherapy, ADT or observation. Such treatment may or may not be applicable for patients enrolled in this study. Regardless, patients will be expected to forgo standard treatment until there is evidence of clinical, biochemical (PSA >50 ng/ml) or radiographic disease progression.

6.10 Costs

Every patient will be provided a copy of the Insurance and Research Participant Financial Responsibility Information Sheet. This document outlines the financial responsibilities for each study test and procedure based on a standard of care versus research analysis.

6.11 Compensation

Patients will not be financially compensated if they join the study.

6.12 Withdrawal from Study

The reasons for withdrawal from the study include:

- Subject withdraws consent for follow-up.
- Subject is lost to follow-up.
- Study is terminated for any reason.

6.13 Cross-over

The men on the control arm are allowed to cross over and be treated with SBRT following documented progression as defined in section 8.6.2.

7. INVESTIGATIONAL AGENT/DEVICE/PROCEDURE INFORMATION

7.1 Investigational Agent - DCFPyL

This information is loaded into the IND application section of eIRB. Additional radiopharmaceutical information not listed below is downloaded on the FDA IND application in the supporting documents section of eIRB and available there for review.

8. STUDY CALENDARS

STUDY CALENDAR: OBSERVATION ARM ONLY

	Pre-Study	Randomization	Day 1[f]	Day 90 from randomization (+/- 7 days)	Day 180 from randomization (+/- 7 days)
Informed Consent	X				
Demographics	X				
Medical History & Review of Medication	X			X	X
Physical Exam	X			X	X
Vital Signs	X				
Height	X				
Weight	X				
Performance Status	X			X	X
CBC w/ Diff[a]	X[e]		X	X	X
LDH & Serum Chemistry[b]	X[e]		X	X	X
Testosterone	X[e]			X	X
PSA	X[h]		X	X	X
CAPP-Seq			X	X	X
Immuno-SEQ[i]			X[i]	X[i]	
Bone scan & CT/MRI of Involved Site[c]	X				X
DCFPyL- PET/CT or PET/MRI[j]			X		X
Rectal Swab			X	X	
Color Test			X[g]		
AE Evaluation and QoL - BPI			X	X	X

a. Including platelets.
b. Albumin, Alkaline Phosphatase (AP), Total Bilirubin, Bicarbonate, BUN, Calcium, Chloride, Creatinine, Glucose, Potassium, Total Protein, SGOT [AST], SPGT [ALT], Sodium.
c. Enrollment bone scan and CT-imaging studies need to be within 3-months of enrollment.
d. See APPENDIX III-VI for details regarding CAPP-Seq. (Correlative Blood draws are within 30 days from the date of randomization)
e. CBC, Serum Chemistry, LDH, and Testosterone within 6 months prior to registration
f. Day 1 denotes the start of Observation Arm. Day 1 must occur within 30 days of randomization.
g. One-time saliva Color Kit collection performed at any point during the study or after (F/U) for ALL patients. h. PSA within 1 month prior to registration
i. Only for first 10 patients randomized to the Observation Arm j. If you received a DCFPyL-PET/MRI or DCFPyL-PET/CT scan within the past 6 months with results and did not demonstrate more disease
more disease lesions than baseline CT/Bone scan, than that scan will be used for Day 1.

STUDY CALENDAR: SBRT ARM ONLY

	Pre-Study		Day 1[i]	SBRT/SABR[j]	Day 90 from randomization (+/- 7 days)	Day 180 from randomization (+/- 7 days)
Informed Consent	X	Randomization				
Demographics	X					
Medical History & Review of Medication	X			X[f]	X	X
Physical Exam	X			X[f]	X	X
Vital Signs	X					
Height	X					
Weight	X					
Performance Status	X				X	X
CBC w/ Diff[a]	X[h]		X		X	X
LDH & Serum Chemistry[b]	X[h]		X		X	X
Testosterone	X[h]				X	X
PSA	X[m]		X		X	X
CAPP-Seq			X[d]		X[d]	X[d]
ImmunoSEQ			X[d]		X[d]	
Epic HD-CTC			X[d]			X[d]
Bone scan & CT/MRI of Involved Site[c]	X					X[g]
DCFPyL- PET/CT or PET/MRI			X[l]			X
Color Test			X[k]			
Rectal Swab			X		X	
AE Evaluation			X	X[f]	X	X
QoL - BPI			X	X[f]	X	X
SBRT/SABR			X	X		

a. Including platelets.
b. Albumin, Alkaline Phosphatase (AP), Total Bilirubin, Bicarbonate, BUN, Calcium, Chloride, Creatinine, Glucose, Potassium, Total Protein, SGOT [AST], SPGT [ALT], Sodium.

c. Enrollment bone scan and CT-imaging studies need to be within 3-months of randomization.
d. See APPENDIX III-V for details regarding CAPP-Seq, EPIC HD-CTC & ImmunoSEQ. (Correlative Blood draws are within 30 days from the date of randomization)
e. Only for CAPP-Seq
f. Can be during SBRT on-treatment visit.
g. Tumor measurements are to be calculated on CT scans at 6 months.
h. CBC, Serum Chemistry, LDH, and Testosterone within 6 months prior to registration
i. Day 1 denotes the start of SBRT Arm. Day 1 must occur within 30 days of randomization.
j. SBRT/SABR should be completed within 60 days from randomization date.
k. One-time saliva Color Kit collection performed at any point during the study or after (F/U) for ALL enrolled patients.
l. If you received a DCFPyL-PET/MRI or DCFPyL-PET/CT scan within the past 6 months with results that did not demonstrate more disease lesions than baseline CT/Bone Scan, then that scan will be used for Day 1.
m. PSA within 1 month prior to registration

9. MEASUREMENT OF EFFECT

9.1 Antitumor Effect – Metastatic Tumors

For the purposes of this study, patients should be re-evaluated for radiographic response 6 months after randomization. Trial radiologists evaluating for treatment responses will be blinded to the treatment group and treatment specifics (lesions treated with SBRT).

Response and progression will be evaluated in three ways:
1) Using Response Evaluation Criteria in Solid Tumors (RECIST 1.1) Committee definition. Changes in only the largest diameter (uni-dimensional measurement) of the tumor lesions are used in the RECIST criteria.
2) Lesions by bone scan will be evaluated as positive, negative or no change. MRI of the bone lesion can be used to clarify equivocal lesions.
3) Serial PSA changes.

9.2 Definitions

Evaluable Population: will consist of all patients who have received SBRT.

Safety Population: Will consist of all subjects who were enrolled and have undergone at least one fraction of SBRT. This will be used to assess the clinical safety and tolerability of the study.

Evaluable for Objective Response: Only those patients who have measurable disease present at baseline, have completed all fractions of SBRT, and have had their disease re-evaluated will be considered evaluable for response. These patients will have their response classified according to the definitions stated below.

9.3 Disease Parameters

Measurable Disease: Measurable lesions are defined as those that can be accurately measured in at least one dimension (longest diameter to be recorded) as $\geq$10 mm with diagnostic techniques (CT, or MRI). All tumor measurements must be recorded in millimeters (or decimal fractions of centimeters). Serial PSA measurements will also be analyzed.

Non-Measurable Disease: All other lesions (or sites of disease), including small lesions (longest diameter <10 mm with diagnostic techniques), are considered non-measurable disease. Bone lesions, leptomeningeal disease, ascites, pleural/pericardial effusions, lymphangitis, abdominal masses (not followed by CT or MRI), and cystic lesions are all non-measurable.

Target Lesions: Target lesions in this study will be considered oligometastatic sites up to a maximum of 3 lesions per patient. They should be recorded and measured at baseline. Target lesions should be equal to or larger than 10 mm in the smallest cross-sectional diameter on CT or MRI and/or any lesion that shows increase uptake on bone scans. A sum of the longest diameter (LD) for all target lesions will be calculated and reported as the baseline sum LD. The baseline sum LD will be used as reference by which to characterize the objective tumor response. For bone scans we will also use a simple evaluation system composed of positive, negative or no change to lesions.

Non-Target Lesions: N/A

9.4 Methods for Evaluation of Measurable Disease

All measurements should be taken and recorded in metric notation using a ruler or calipers. All baseline evaluations should be performed as closely as possible to the beginning of treatment and never more than 4 weeks before the beginning of the treatment.

The same method of assessment and the same technique should be used to characterize each identified and reported lesion at baseline and during follow-up. Imaging-based evaluation is preferred to evaluation by clinical examination when both methods have been used to assess the antitumor effect of a treatment.

Conventional CT, PET/CT, and MRI. These techniques should be performed with cuts of 10 mm or less in slice thickness contiguously. Spiral CT should be performed using a 5 mm contiguous reconstruction algorithm. This applies to tumors of the chest, abdomen, and pelvis. Head and neck tumors and those of extremities usually require specific protocols.

Secondary objectives for comparison of DCFPyL-PET/MRI or –PET/CT to bone scan at baseline and 1-year following SBRT/SABR. We will estimate 1) the proportion of metastatic lesions found on bone scan that are DCFPyL-PET/MRI or –PET/CT positive and 2) the proportion of DCFPyL-PET/MRI or –PET/CT positive sites that are positive for metastatic disease by bone scan for prostate cancer. The results of the DCFPyL-PET/MRI or –PET/CT will not be made available to the treating physicians until after the trial is completed and these results will not be made available to the patient.

9.5 Response Criteria

9.5.1 Evaluation of Target Lesions/PSA Response

Complete Response (CR): Disappearance of all target lesions and PSA < pre SBRT PSA

Partial Response (PR): At least a 30% decrease in the sum of the longest diameter (LD) of target lesions, taking as reference the baseline sum LD. Or a third of the lesions are negative or no change by bone scan and PSA ≤ pre-SBRT PSA.

Progressive Disease (PD): At least a 20% increase in the sum of the LD of target lesions, taking as reference the smallest sum LD recorded since the treatment started or the appearance of ≥1 new lesion(s). Or ≥1 new lesion(s) appear by bone scan. Or PSA ≥25% increase in PSA from nadir or > 50 ng/ml.

Stable Disease (SD): Neither sufficient shrinkage to qualify for PR nor sufficient increase to qualify for PD, taking as reference the smallest sum LD since the treatment started. Or PSA ≥ pre-SBRT PSA, but not ≥25% increase in PSA from nadir and <50 ng/ml.

9.5.2 Evaluation of Non-Target Lesions

N/A

9.5.3 Evaluation of Best Overall Response

The best overall response is the best response recorded from the start of the treatment until disease progression/recurrence (taking as reference for progressive disease the smallest measurements recorded since the treatment started). Best overall response will be based on the overall response of the target lesions.

9.6 Imaging Reference Standard, as Applicable (i.e., "Gold standard")

Reference standard will be determined by a truth panel consisting of a nuclear medicine physician, radiologist, and GU oncologist. This panel will determine whether individual sites of suspected metastatic disease on initial baseline CIM (bone scan and chest/abdomen/pelvis CT) and DCFPyL-PET/MRI or -PET/CT findings are truly positive based on following data:

- prior available imaging
- available follow-up imaging obtained as part of clinical care and/or investigational protocol and treatment history for up to 12 months from the time of DCFPyL-PET/MRI or –PET/CT scan.

- The results of the DCFPyL-PET/MRI or –PET/CT will not be made available to the treating physicians until after the trial is completed and these results will not be made available to the patient.

9.6.1 Duration of Response

Response will be defined as evidence of CR, PR, or stable disease. The duration of response will be measured from the start of treatment until the criteria for progression are met.

Duration of CR or PR: The duration of CR or PR will be recorded from the time measurement criteria are met for CR or PR (whichever is first recorded) until the first date that current or progressive disease is objectively documented (taking as reference for progressive disease the smallest measurements recorded since the treatment started).

Duration of Stable Disease: Stable disease is measured from the start of the treatment until the criteria for progression are met, taking as reference the smallest measurements recorded since the treatment started.

9.6.2 Clinical Response Parameters

Progression is a composite endpoint defined from the Prostate Cancer Working Group 2 (PCWG2) criteria for metastatic castrate resistant prostate cancer (mCRPC) (75) and our previous trials in a population of men with biochemical failure without metastases (72-74). Progression will be defined as either: 1) a ≥25% increase in PSA from nadir (and by ≥2 ng/mL), requiring confirmation ≥4 weeks later (PCWG2 criteria); and/or, 2) clinical/radiographic-progression defined as symptomatic progression (worsening disease-related symptoms or new cancer-related complications), or radiologic progression (on CT scan: ≥20% enlargement in sum diameter of soft-tissue target lesions [RECIST 1.1 criteria]; on bone scan: ≥1 new bone lesions), initiation of ADT or death due to any cause, whichever occurs first. Death is considered a severe adverse event here.

Progression Free Survival (PFS) is defined as the time from starting treatment to the time of progression as defined above. Subjects who do not progress will be censored at the time of the last contact.

ADT Free Survival (ADT-FS) is defined as the time from starting treatment to the time of initiation of palliative ADT. ADT will typically be initiated on tumor progression and/or development of new metastases. Subjects who do not start ADT will be censored at the time of the last contact.

Time to Progression (TTP) is defined as the time from starting treatment to the time of first documented tumor progression or new lesions by CT and/or bone scan or initiation of ADT. Subjects who do not progress will be censored at the time of the last contact. In addition, death from any cause will also be censored.

Overall Survival (OS) is defined as the time from starting treatment until death due to any cause. For subjects who do not die, time to death will be censored at the time of last contact.

Locoregional Control (LRC) is defined as the time from starting treatment until local and/or regional relapse is documented

9.6.3 Response Review

All responses will be reviewed by the study co-investigator radiologists.

10. ADVERSE EVENT REPORTING REQUIREMENTS

10.1 General

Adverse event collection and reporting is a routine part of every clinical trial. This study will use the descriptions and grading scales found in the NCI Common Terminology Criteria for Adverse Events version 4.0 (CTCAE v 4.0) that is available at http://ctep.cancer.gov/reporting//ctc.html.

Information on all adverse events, whether reported by the participant, directly observed, or detected by physical examination, laboratory test or other means, will be collected, recorded, followed and reported as described in the following sections.

Participants should be instructed to report any serious post-study event(s) that might reasonably be related to participation in this study. The investigator should notify the IRB and any other applicable regulatory agency of any unanticipated death or adverse event occurring after a participant has discontinued or terminated study participation that may reasonably be related to the study.

10.2 Definitions

10.2.1 Adverse Event (AE)

An adverse event is any undesirable sign, symptom or medical condition or experience that develops or worsens in severity after starting the first dose of study treatment or any procedure specified in the protocol, even if the event is not considered to be related to the study.

Abnormal laboratory values or diagnostic test results constitute adverse events only if they induce clinical signs or symptoms or require treatment or further diagnostic tests.

10.2.2 Serious adverse event (SAE)

A serious adverse event is an undesirable sign, symptom, or medical condition which:

- is fatal or life-threatening;
- requires or prolongs inpatient hospitalization;
- results in persistent or significant disability/incapacity;
- constitutes a congenital anomaly or birth defect; or
- jeopardizes the participant and requires medical or surgical intervention to prevent one of the outcomes listed above.

Events **not** considered to be serious adverse events are hospitalizations for:

- routine treatment or monitoring of the studied indication, not associated with any deterioration in condition, or for elective procedures
- elective or pre-planned treatment for a pre-existing condition that did not worsen
- emergency outpatient treatment for an event not fulfilling the serious criteria outlined above and not resulting in inpatient admission
- respite care

10.2.3 Expectedness

- Expected: Expected adverse events are those that have been previously identified as resulting from administration of the agent. For the purposes of this study, an adverse event is considered expected when it appears in the current adverse event list, the Investigator's Brochure, the package insert or is included in the informed consent document as a potential risk.
- Unexpected: An adverse event is considered unexpected when it varies in nature, intensity or frequency from information provided in the current adverse event list, the Investigator's Brochure, the package insert or when it is not included in the informed consent document as a potential risk

10.2.4 Attribution

Attribution is the relationship between an adverse event or serious adverse event and the study treatment. Attribution will be assigned as follows:

- Definite – The AE is clearly related to the study treatment.
- Probable – The AE is likely related to the study treatment.
- Possible – The AE may be related to the study treatment.
- Unlikely – The AE is doubtfully related to the study treatment.
- Unrelated - The AE is clearly NOT related to the study treatment.

10.3 Potential Adverse Events

Signs and symptoms of disease progression are not considered AEs. The development of a new cancer should be regarded as an AE. New cancers are those that are not the primary reason for administration of study treatment and have been identified after inclusion of the patient into the clinical study.

Because patients are receiving standard treatments, which are not part of this study, their treating physician will be counseling them on the risk of their treatments, including the risk of surgery, radiation therapy, and/or chemotherapy, whichever is appropriate for the type and the stage of their cancer. The procedures related to the study are phlebotomy, PET imaging, SBRT, and DCFPyL administration.

Phlebotomy can cause pain, bleeding, and rare needle site infection. PET imaging results in low dose radiation exposure (see Investigator's Brochure for details of dosimetry), which has an extremely small risk of causing a secondary cancer.

10.4 Stereotactic Body Radiation Treatment (SBRT) or Stereotactic Ablative Radiation Treatment (SABR)

It is difficult at this time to predict with confidence the complication rate from the proposed SBRT/SABR; however, it is reasonable to extrapolate from the current experience with SBRT/SABR to the lung, prostate, spine, liver and pancreas. One significant toxicity is radiation pneumonitis, which can be manifested as fever, increased excertional dypsnea, pleuritic chest pain, and peritumoral infiltrate on chest imaging. It generally occurs between 1 to 3 months of completion of radiotherapy. The risk of grade 2-4 radiation pneumonitis is aproximately 10-15% in patients treated with standard fractionated large field radiotherapy and higher in patients treated with combined chemoradiotherapy. It is highly dependent on the volume of the lung treated to high dose and the mean lung dose. At this point, the incidence of RT pneumonitis from stereotactic radiosurgery for small pulmonary tumors is unknown. However, if the treated tumor volume is kept ≤ 65 cc, the risk should be < 10-15% with the proposed dose level.

Other toxicities commonly associated with such treatment includes dysphagia, odynophagia, nausea, vomiting, anorexia, and weight loss. Some of these symptoms can also be due to tumor progression. Clinical and radiographic assessments will be performed as indicated to identify all adverse effects, ascertain their etiology, and provide the most appropriate palliative measures. Complications orther than radiation pneumonitis, if any, will be graded according to the Common Toxicity Criteria, National Cancer Institute, version 4.0.

10.5 DCFPyL

10.5.1 [^{18}F]DCFPyL is eluted with 1 mL of ethanol followed by 10 mL of 0.9% sodium chloride, via a sterilizing 0.22µ filter into a sterile vial containing 4 mL 0.9% sodium chloride. Test radiopharmaceutical will be administered intravenously via slow I.V. push. The maximum mass dose of the ligand corresponding to the 10 mCi dose of [^{18}F]DCFPyL will be less than 4.02µg per administration, although the actual dose will be significantly less than depending on the specific activity achieved during each radiosynthesis run. The lowest limit for

specific-activity (SA) we will use at the time of injection is 1000 mCi/μmole, although the validation and phase I study radiosynthesis SA was significantly higher than 1000 mCi/μmole.

Preclinical Toxicology

A toxicity report "Single Dose Toxicity Study of ^{19}F- DCFBC (NSC-743104) in Rats" was prepared by Bridge GPS, Inc. (Study No. 1535-07015). The following summary is taken from that report.

This study was designed to determine target organ toxicity of 19F- DCFBC (NSC-743104) and its reversibility in rats treated with a single intravenous dose.

Sixty Fischer 344 rats (30/sex) were randomly assigned to one of three dose groups (10/sex/group) based on body weight and physical examination. Five rats/sex/group were designated to the main phase of the study, and five rats/sex/group were designated to the recovery phase of the study. All animals were dosed once on Study Day (SC) 1 via intravenous injection (tail vein) with either the control article (5% dextrose) or 19F- DCFBC (NSC-743104) at nominal dose levels of 0.1 or 0.5 mg/kg. Terminal sacrifice necropsies were performed on SD 4; recovery sacrifice necropsies were performed on SD 15. Parameters evaluated during the study included mortality, clinical and cage side observations, body weights, body weight changes, clinical pathology parameters (clinical chemistry and hematology), gross pathology and histopathology.

Mortality, clinical and cage-side observation, body weight and body weight changes, clinical pathology, gross pathology and histopathology were unaffected by treatment.

Based on the results of this study, a single intravenous injection of 19F-DCFBC (NSC-743104) at doses up to 0.5 mg/kg to male and female Fischer 344 rats was well tolerated.

Table 1: Tissue distribution of [^{18}F] DCFBC (%ID/gm)

	5 min n=5	15 min n=4	30 min n=4	60 min n=4	120min n=3
Blood	11.34	4.07	2.26	1.80	0.36
heart	4.38	2.01	1.24	0.77	0.27
lung	7.03	3.21	1.78	1.10	0.36
liver	5.99	4.07	4.18	5.12	2.11
stomach	2.87	1.35	0.81	0.46	1.08
pancreas	2.17	1.04	0.99	0.54	0.18
spleen	8.76	4.27	1.94	1.58	0.44
fat	1.75	1.56	0.67	1.05	0.26
kidney	63.40	62.94	51.29	41.55	13.08
bone	2.88	1.61	1.72	1.73	2.46
muscle	2.21	1.00	0.45	0.57	0.24
small intestine	4.29	2.31	1.20	0.66	0.15
large intestine	2.82	1.47	0.78	0.58	0.27
bladder	55.41	15.94	15.18	14.49	2.57
PC-3 PIP	8.17	6.03	6.16	8.16	4.69
PC-3 flu	3.47	1.72	0.97	0.77	0.18
cortex	0.49	0.25	0.13	0.10	0.06
cerebellum	0.58	0.26	0.14	0.10	0.04
PIP: flu	2.36	3.51	6.35	10.57	26.65

Similar toxicity was observed for our second generation compound DCFPyL and was awarded a physician sponsored FDA exploratory IND based in this acceptable pre-clinical data (IND#121,064) for ^{18}F-DCFPyL as a PET radiopharmaceutical, which is held by the co-I, Dr. Martin Pomper.

<u>Initial Phase I study Adverse Events</u>
An initial phase I study of the biodistribution and dosimetry of DCFBC was performed in five men with metastatic prostate cancer from 10/2010 through 12/2010. No serious adverse events (SAE) were observed. One patient with history of hypertension experienced grade III hypertension after administration of DCFBC with no clinical symptoms. Twenty-four hours later his blood pressure returned to baseline grade 2 hypertension and at 7 days'

post DCFBC administration, his blood pressure normalized to baseline levels. The attribution of this hypertension adverse event (AE) was listed as unlikely (the adverse event is doubtfully related to the investigational imaging tracer) given this patient had been previously controlled with a calcium-channel blocker medication but this was discontinued about two months prior to DCFBC administration and his blood pressure was more likely attributed to stress during the PET/CT scan and overall labile blood pressure of his calcium-channel blocker. No other adverse events noted in all five patients over a 28-day follow-up post study radiopharmaceutical administration.

Human Dosimetry

In our biodistribution and dosimetry study, five patients with radiologic evidence of metastatic prostate cancer were studied after intravenous administration of 370 MBq (10 mCi) of DCFBC. Serial PET imaging was performed out to 2 hours after administration. Time-activity curves were generated for selected normal tissues and metastatic foci. Radiation dose estimates were calculated using OLINDA/EXM 1.1. Most vascular organs demonstrated a slow decrease in activity concentration over time consistent with clearance from blood pool, with primarily urinary radiotracer excretion. The organ with the highest mean absorbed dose (μGy/MBq) is the urinary bladder wall (32.4), followed by the stomach wall (30.2), heart wall (29.2), and kidneys (28.4). The remaining GI tract organs (small intestines, SI; upper large intestines, ULI; lower large intestines, LLI), liver and lungs receive lower absorbed doses. The mean effective dose was 19.9 ± 1.34 μSv/MBq. Dose estimates for ^{18}F- DCFBC are comparable to those of other PET radiopharmaceuticals such as ^{18}F-fluorodeoxyglucose (^{18}F-FDG). (Note: This study has been accepted for publication in the Journal of Nuclear Medicine in the fall of 2012 (35) and reported this dosimetry data to the FDA as part of a protocol revision for Protocol/IND 108,943 on 8/30/2011.

Table 2: Human Average Organ Absorbed Dose (mGy/MBq) and estimated effective dose (mSv/MBq)

	Average	Std Dev	COV
Adrenals	1.85E-02	2.83E-03	15.32%
Brain	4.21E-03	2.83E-04	6.73%
Breasts	8.51E-03	3.22E-04	3.78%
GB Wall	1.79E-02	1.95E-03	10.90%
LLI Wall	2.47E-02	3.69E-03	14.92%
SI Wall	2.36E-02	1.72E-03	7.31%
Stomach Wall	3.02E-02	3.24E-03	10.72%
ULI Wall	2.34E-02	2.20E-03	9.39%

Heart Wall	2.92E-02	3.24E-03	11.12%
Kidneys	2.84E-02	3.81E-03	13.45%
Liver	2.46E-02	4.16E-03	16.88%
Lungs	2.45E-02	2.99E-03	12.22%
Muscle	9.69E-03	3.97E-04	4.10%
Ovaries	1.32E-02	5.26E-04	3.99%
Pancreas	1.92E-02	2.15E-03	11.19%
Red Marrow	1.70E-02	9.81E-04	5.79%
Osteogenic Cells	1.82E-02	8.92E-04	4.90%
Skin	7.30E-03	3.50E-04	4.79%
Spleen	1.72E-02	1.05E-03	6.08%
Testes	1.54E-02	4.19E-03	27.23%
Thymus	1.10E-02	4.53E-04	4.12%
Thyroid	1.17E-02	6.87E-04	5.88%
Bladder Wall	3.24E-02	7.24E-03	22.35%
Uterus	1.34E-02	2.95E-04	2.20%
Total Body	1.09E-02	4.28E-04	3.91%
ED	1.99E-02	1.34E-03	6.73%

10.6 Reporting Procedures

10.6.1 General

Adverse events will be recorded at each visit. If an adverse event requiring medical attention occurs between visits, this will be recorded as well. The variables to be recorded for each adverse event include, but are not limited to, onset, resolution, intensity, action taken, outcome, causality rating and whether it constitutes an SAE or not. The intensity of the adverse event should be captured using CTCAE criteria, version 4.0, when possible.

All Serious Adverse Events (SAEs) will be reported via the AdEERS (Adverse Event Expedited Reporting System) application accessed via the CTEP web site (https://webapps.ctep.nci.nih.gov/openapps/plsql/gadeers_main$.startup).

All adverse events will be captured on the appropriate study-specific case report forms (CRFs).

10.6.2 Expedited FDA Reporting Requirements for Unexpected and Related Serious Adverse Events (per 21CFR312.32)

7 Calendar-Day Telephone or Fax IND Safety Report

The Sponsor-Investigator is required to notify the FDA of any fatal or life-threatening adverse event that is unexpected and assessed by the Sponsor-Investigator to be possibly related to the use of DCFPyL within 7 calendar-days of first learning of the event. An unexpected adverse event deemed possibly related to the use of an investigational study drug is defined as any adverse drug experience of which the specificity or severity is not consistent with the current investigator brochure, the general investigational plan, or elsewhere in the current application, as amended.

Such reports are to be telephoned or faxed to the FDA within 7 calendar-days of first learning of the event. Each telephone call or fax transmission should be directed to the FDA new drug review division in the Center for Drug Evaluation and Research or in the product review division for the Center for Biologics Evaluation and Research, whichever department is responsible for the review of the IND.

15 Calendar-Day Written IND Safety Report

The Sponsor-Investigator (Dr. Martin Pomper) is required to notify the FDA, and all participating investigators (as applicable), in a written IND Safety Report, of any serious, unexpected adverse event considered by the Sponsor-Investigator to be possibly related to the use of DCFPyL within 15 calendar days of first learning of the event. If applicable, the Sponsor-Investigator must also notify the FDA, and all participating investigators (as applicable), of any finding from tests in laboratory animals that suggests a significant risk for human subjects including reports of mutagenicity, teratogenicity, or carcinogenicity within 15 calendar-days of first learning of the event.

A serious, unexpected adverse event deemed possibly related to the use of an investigational study drug is any adverse drug experience of which the specificity or severity is not consistent with the current investigator brochure, the general investigational plan, or elsewhere in the current application, as amended, and results in any of the following outcomes:

- Death (report first as a 7-day telephone/fax report);
- life-threatening adverse drug experience (report first as a 7-day telephone/fax report);
- inpatient hospitalization or prolongation of existing hospitalization; a persistent or significant disability/incapacity, or a congenital anomaly/birth defect; or is an important medical event that may not result in death, be life-threatening, or require hospitalization but is considered a serious adverse drug experience when, based upon appropriate medical judgment, it may jeopardize the patient or subject and may require medical or surgical intervention to prevent one of the outcomes listed in this definition.

All written IND Safety Reports should include an Analysis of Similar Events in accordance with 21CFR312.32. All safety reports previously filed with the IND concerning similar events should be analyzed. The new report should contain comments on the significance of the new event in light of the previous, similar reports and be submitted to the FDA, Abbott Laboratories, and all participating investigators (as applicable), within 15 calendar-days of first learning of the event. The FDA prefers these reports be documented on a MedWatch 3500A Form, but alternative formats are acceptable (e.g., summary letter). This form is available at http://www.fda.gov/medwatch/report/hcp.htm.

Follow-up Reports

All follow-up information concerning IND Safety Reports should be submitted to the FDA as soon as possible.

10.6.3 Institutional Review Board

All adverse events and serious adverse events will be reported to the IRB per current institutional standards. If an adverse event requires modification of the informed consent, these modifications will be provided to the IRB with the report of the adverse event. If an adverse event requires modification to the study protocol, these modifications will be provided to the IRB as soon as is possible.

11. DATA AND SAFETY MONITORING PLAN

This is a DSMP Level I study under the SKCCC Monitoring Plan (see Appendix VI). A Level I study requires both internal and external data monitoring. The Principal Investigator is responsible for internal monitoring for both safety and data quality. External data monitoring will be performed by the SKCCC at Johns Hopkins Clinical Research Office Quality Assurance Program (CRO QA).

Data and safety monitoring oversight will be conducted by the SKCCC at Johns Hopkins Safety Monitoring Committee. Per the SKCCC at Johns Hopkins Safety Monitoring plan, the CRO AQ will forward summaries of all monitoring reports to the Safety Monitoring Committee for review. All reportable anticipated and unanticipated protocol events/problems and amendments that are submitted to the IRB will also be reviewed by the Safety Monitoring Committee Chair (or designee) and QA manager.

12. REGULATORY CONSIDERATIONS

12.1 Protocol Review and Amendments

Information regarding study conduct and progress will be reported to the Institutional Review Board (IRB) per the current institutional standards.

Any changes to the protocol will be made in the form of an amendment and must be approved by the IRB prior to implementation.

12.2 Informed Consent

The investigator (or his/her designee) will explain to each subject the nature of the study, its purpose, the procedures involved, the expected duration, the potential risks and benefits involved and any discomfort it may entail. Each subject will be informed that participation in the study is voluntary, that she may withdraw from the study at any time, and that withdrawal of consent will not affect her subsequent medical treatment or relationship with the treating physician(s) or institution. The informed consent will be given by means of a standard written statement, written in non-technical language, which will be IRB approved. The subject should read and consider the statement before signing and dating it, and will be given a copy of the document. No subject will enter the study or have study-specific procedures done before his/her informed consent has been obtained.

In accordance with the Health Information Portability and Accountability Act (HIPAA), the written informed consent document (or a separate document to be given in conjunction with the consent document) will include a subject authorization to release medical information to the study sponsor and supporting agencies and/or allow these bodies, a regulatory authority, or Institutional Review Board access to subjects' medical information that includes all hospital records relevant to the study, including subjects' medical history.

12.3 Ethics and GCP

This study will be carried out in compliance with the protocol and Good Clinical Practice, as described in:

1. ICH Harmonized Tripartite Guidelines for Good Clinical Practice 1996.
2. US 21 Code of Federal Regulations dealing with clinical studies (including parts 50 and 56 concerning informed consent and IRB regulations).
3. Declaration of Helsinki, concerning medical research in humans (Recommendations Guiding Physicians in Biomedical Research Involving Human Subjects, Helsinki 1964, amended Tokyo 1975, Venice 1983, Hong Kong 1989, Somerset West 1996).

The investigator agrees to adhere to the instructions and procedures described in it and thereby to adhere to the principles of Good Clinical Practice that it conforms to.

13. STATISTICAL CONSIDERATIONS

13.1 Endpoints

13.1.1 Primary Objective

To determine the proportion of patients with oligometastatic hormone sensitive prostate cancer who have progressed after 6 months from randomization to observation *versus* stereotactic body radiation therapy (SBRT).

13.1.2 Secondary Objectives

- o To describe the toxicity of SBRT/SABR delivered for the population enrolled using grading with CTCAE v. 4.0
- o To determine local control at 6-months after SBRT/SABR in patients with oligometastatic disease.
- o To assess progression free survival (PFS) of this patient population after randomization defined as the time interval between the day of randomization and progression.
- o To assess ADT-free survival (ADT-FS) of this patient population after randomization defined as the time interval between the day of randomization and the initiation of ADT. ADT will typically be initiated on progression and/or development of new metastases.
- o To assess quality of life following of the observation *versus* SBRT/SABR arms. Brief Pain Inventory form which will be filled out by the patient at the treatment response intervals outlined above.
- o Secondary objectives for comparison of DCFPyL-PET/MRI or -PET/CT to bone scan at baseline and 1-year following SBRT/SABR. We will estimate 1) the proportion of metastatic lesions found on bone scan that are DCFPyL-PET/MRI or –PET/CT positive and 2) the proportion of DCFPyL-PET/MRI or PET/CT positive sites that are positive for metastatic disease by bone scan for prostate cancer. Each of these sites of metastatic disease will also be further characterized according to specific subcategories based on features of the lesion as follows for each modality and their specific features:

- · Bone scan
  - Axial skeleton (spine, pelvis, sacrum, sternum, skull, ribs)
  - Appendicular skeleton (arms, legs)
  - Stable versus new or progressive metastatic site
- · CT scan
  - Axial versus appendicular skeleton for bone lesions
  - Size of soft tissue lesions (greatest transaxial and perpendicular diameters)

- Location of soft tissue lesion

· MRI scan
- Axial versus appendicular skeleton for bone lesions
- Size of soft tissue lesions (greatest transaxial and perpendicular diameters)
- Location of soft tissue lesion

· PET
- Bone versus lymph node versus organ involvement
- PET-based SUV parameters (SUVpeak, SUVmax)

13.2 Sample Size/Accrual Rate

The primary endpoint will be rate of progression at 6-months. Historical data on this patient population indicate that >80% would show progression as defined above within a 6-month period without treatment, and thus this is the progression rate we expect in subjects in the control/observation arm (72-74). We hypothesize that SBRT will be able to reduce the progression at 6-months by 50% (76). A sample size using a 1:2 randomization scheme of 18 patients in the control group and 36 in the SBRT arm will provide 85% power to detect a decrease relapse rate from 80% to 40% with a type I error = 0.05 using one-sided Fisher's exact test. Thus, we will have a total of 54 patients.

Patients who drop out within one month of enrollment or prior to day 1 of SBRT or Observation will be replaced. We will accrue 54 evaluable patients and will update total accrual number for the study if necessary.

There will be no interim analysis for futility, since the progression endpoint will not have been reached by a meaningful number of patients before full accrual.

13.3 Early Stopping Guidelines:

This study will monitor site-specific grade 4/5 toxicity in the SBRT arm. If it becomes evident that the proportion of grade 4/5 toxicity at specific sites convincingly exceeds 20%, the study will be halted for a safety consultation. Specifically, we will apply a Bayesian toxicity monitoring rule that suspends the enrollment if the posterior probability of toxicity being larger than 20% threshold is 75% or higher. The monitoring rule uses Beta (0.5, 5.5) as prior distribution. This means that our prior guess of the proportion of toxicity is 8.3%, and there is 90% chance that this proportion is 0.04%-30.6%. The monitoring will start from the first patient, and the decision rule for safety stopping is as follows:

Stop if:

**# grade 4/5 toxicity >=**	3	4	5	6	7	8	9	10
**Out of # patients**	3 - 5	6 - 10	11 - 14	15 - 18	19 - 23	24 - 27	28 - 32	33 - 36

The operating characteristics of the stopping rule are shown below and are based on 5000 simulations:

True AE rate	% simulated trials declaring unsafe	Average sample size
0.10	2.6	35.3
0.2	31.5	29.6
0.25	56.4	24.6
0.3	78.4	19.2
0.35	91.9	14.7

13.4 Analysis of Primary Objective

This is a (1:2) randomized, Phase II trial of observation *versus* SBRT in oligometastatic hormone sensitive prostate cancer patients. Minimization approach (77) will be applied to ensure balanced assignment to each treatment arm by stratification factors: 1) Initial treatment with surgery vs. radiation therapy; 2) Prior hormonal therapy vs. no prior hormonal therapy; and 3) PSADT <6 mos vs. 6-14.9 mos. Baseline PSA level is defined as that measured Day 1 following randomization. Progression is defined as per section 9.6.2.

The primary outcome of interest is the proportion of patients who have progressed after 6 months from randomization. For each arm, we will calculate the proportion of patients who have progressed and exact 95% confidence intervals. If a patient has withdrawn from the study before 6 months, they will be considered to have progressed when calculating the proportion of individuals who have progressed. We will compare the proportion of patients who have progressed in the observation and SBRT arms using Fisher's exact test. The analysis population includes all randomized subjects based on intend-to-treat principle.

13.5 Analysis of Secondary Objectives

- For safety analysis, adverse events will be summarized by type and grade.
- Hazard rate estimates and 95% confidence intervals as well as Kaplan-Meier (KM) estimates will be used to summarize progression free survival (PFS), ADT free survival (ADT-FS), time to locoregional progression (TTLP) and time to distant progression (TTDP), duration of response functions over time. The median PFS, ADT-FS, TTLP and TTDP will be reported.
- The efficacy of SBRT/SABR with oligometastatic disease will also be determined by measuring local control of each lesion at 6-months. Each metastatic lesion will be considered a target lesion and independently evaluated for response using RECIST 1.1 or bone scan evaluation criteria above. The lesion will be coded as being locally controlled if it is considered stable radiographic disease or if there is evidence of a partial or complete response. Local control assessment will start at three months following randomization and continuous assessment will be pursued during the follow-up period. The proportion of the lesions that have a stable or better response will be estimated using generalized estimating equation.

- Quality of life will be assessed using the Brief Pain Inventory form. An overall score will be calculated pre-treatment and at the time of the 2nd radiologic reassessment. The change in score will be evaluated with a paired t-test.

13.6 Evaluation of Toxicity

All patients who receive at least one fraction of SBRT/SABR will be evaluable for toxicity from the time of their first treatment for SBRT.

13.7 Correlative Science

Descriptive statistics will be performed to correlate the temporal CTC, ctDNA and immune repertoire metric changes with clinical outcomes.

14. REFERENCES

1. Hellman S, Weichselbaum RR. Oligometastases. *J Clin Oncol.* 1995 Jan; 13(1): 8-10.
2. Bacci G, Rocca M, Salone M, Balladelli A, Ferrari S, Palmerini E, Forni C, Briccoli A. High grade osteosarcoma of the extremities with lung metastases at presentation: Treatment with neoadjuvant chemotherapy and simultaneous resection of primary and metastatic lesions. *J Surg Oncol.* 2008 Nov 1; 98(6): 415-420.
3. Fong Y, Cohen AM, Fortner JG, Enker WE, Turnbull AD, Coit DG, Marrero AM, Prasad M, Blumgart LH, Brennan MF. Liver resection for colorectal metastases. *J Clin Oncol.* 1997 Mar; 15(3): 938-946.
4. Steele G,Jr, Bleday R, Mayer RJ, Lindblad A, Petrelli N, Weaver D. A prospective evaluation of hepatic resection for colorectal carcinoma metastases to the liver: Gastrointestinal tumor study group protocol 6584. *J Clin Oncol.* 1991 Jul; 9(7): 1105-1112.
5. Simmonds PC, Primrose JN, Colquitt JL, Garden OJ, Poston GJ, Rees M. Surgical resection of hepatic metastases from colorectal cancer: A systematic review of published studies. *Br J Cancer.* 2006 Apr 10; 94(7): 982-999. PMCID: PMC2361241.
6. Schefter TE, Kavanagh BD, Timmerman RD, Cardenes HR, Baron A, Gaspar LE. A phase I trial of stereotactic body radiation therapy (SBRT) for liver metastases. *Int J Radiat Oncol Biol Phys.* 2005 Aug 1; 62(5): 1371-1378.
7. Okunieff P, Petersen AL, Philip A, Milano MT, Katz AW, Boros L, Schell MC. Stereotactic body radiation therapy (SBRT) for lung metastases. *Acta Oncol.* 2006; 45(7): 808-817.
8. Lo SS, Fakiris AJ, Teh BS, Cardenes HR, Henderson MA, Forquer JA, Papiez L, McGarry RC, Wang JZ, Li K, Mayr NA, Timmerman RD. Stereotactic body radiation therapy for oligometastases. *Expert Rev Anticancer Ther.* 2009 May; 9(5): 621-635.
9. Milano MT, Katz AW, Muhs AG, Philip A, Buchholz DJ, Schell MC, Okunieff P. A prospective pilot study of curative-intent stereotactic body radiation therapy in patients with 5 or fewer oligometastatic lesions. *Cancer.* 2008 Feb 1; 112(3): 650-658.
10. Milano MT, Katz AW, Schell MC, Philip A, Okunieff P. Descriptive analysis of oligometastatic lesions treated with curative-intent stereotactic body radiotherapy. *Int J Radiat Oncol Biol Phys.* 2008 Dec 1; 72(5): 1516-1522.
11. Le QT, Loo BW, Ho A, Cotrutz C, Koong AC, Wakelee H, Kee ST, Constantinescu D, Whyte RI, Donington J. Results of a phase I dose-escalation study using single-fraction stereotactic radiotherapy for lung tumors. *J Thorac Oncol.* 2006 Oct; 1(8): 802-809.
12. Gibbs IC, Kamnerdsupal phon P, Ryu MR, Dodd R, Kiernan M, Chang SD, Adler JR,Jr. Image-guided robotic radiosurgery for spinal metastases. *Radiother Oncol.* 2007 Feb; 82(2): 185-190.
13. Lo SS, Fakiris AJ, Abdulrahman R, Henderson MA, Chang EL, Suh JH, Timmerman RD. Role of stereotactic radiosurgery and fractionated stereotactic radiotherapy in pediatric brain tumors. *Expert Rev Neurother.* 2008 Jan; 8(1): 121-132.
14. Levine AM, Coleman C, Horasek S. Stereotactic radiosurgery for the treatment of primary sarcomas and sarcoma metastases of the spine. *Neurosurgery.* 2009 Feb; 64(2 Suppl): A54-9.
15. Goodman KA, Wiegner EA, Maturen KE, Zhang Z, Mo Q, Yang G, Gibbs IC, Fisher GA, Koong AC. Dose-escalation study of single-fraction stereotactic body radiotherapy for liver malignancies. *Int J Radiat Oncol Biol Phys.* 2010 Oct 1; 78(2): 486-493.
16. Onishi H, Shirato H, Nagata Y, Hiraoka M, Fujino M, Gomi K, Niibe Y, Karasawa K, Hayakawa K, Takai Y, Kimura T, Takeda A, Ouchi A, Hareyama M, Kokubo M, Hara R, Itami J, Yamada K, Araki T. Hypofractionated stereotactic radiotherapy (HypoFXSRT) for stage I non-small cell lung cancer: Updated results of 257 patients in a japanese multi-institutional study. *J Thorac Oncol.* 2007 Jul; 2(7 Suppl 3): S94-100.
17. Baumann P, Nyman J, Hoyer M, Wennberg B, Gagliardi G, Lax I, Drugge N, Ekberg L, Friesland S, Johansson KA, Lund JA, Morhed E, Nilsson K, Levin N, Paludan M, Sederholm C, Traberg A, Wittgren L, Lewensohn R. Outcome in a prospective phase II trial of medically inoperable stage I non-small-cell lung cancer patients treated with stereotactic body radiotherapy. *J Clin Oncol.* 2009 Jul 10; 27(20): 3290-3296.

18. Timmerman R, Paulus R, Galvin J, Michalski J, Straube W, Bradley J, Fakiris A, Bezjak A, Videtic G, Johnstone D, Fowler J, Gore E, Choy H. Stereotactic body radiation therapy for inoperable early stage lung cancer. *JAMA.* 2010 Mar 17; 303(11): 1070-1076. PMCID: PMC2907644.
19. Videtic GM, Stephans K, Reddy C, Gajdos S, Kolar M, Clouser E, Djemil T. Intensity-modulated radiotherapy-based stereotactic body radiotherapy for medically inoperable early-stage lung cancer: Excellent local control. *Int J Radiat Oncol Biol Phys.* 2010 Jun 1; 77(2): 344-349.
20. Fakiris AJ, McGarry RC, Yiannoutsos CT, Papiez L, Williams M, Henderson MA, Timmerman R. Stereotactic body radiation therapy for early-stage non-small-cell lung carcinoma: Four-year results of a prospective phase II study. *Int J Radiat Oncol Biol Phys.* 2009 Nov 1; 75(3): 677-682.
21. Grills IS, Mangona VS, Welsh R, Chmielewski G, McInerney E, Martin S, Wloch J, Ye H, Kestin LL. Outcomes after stereotactic lung radiotherapy or wedge resection for stage I non-small-cell lung cancer. *J Clin Oncol.* 2010 Feb 20; 28(6): 928-935.
22. Wulf J, Haedinger U, Oppitz U, Thiele W, Mueller G, Flentje M. Stereotactic radiotherapy for primary lung cancer and pulmonary metastases: A noninvasive treatment approach in medically inoperable patients. *Int J Radiat Oncol Biol Phys.* 2004 Sep 1; 60(1): 186-196.
23. Hof H, Hoess A, Oetzel D, Debus J, Herfarth K. Stereotactic single-dose radiotherapy of lung metastases. *Strahlenther Onkol.* 2007 Dec; 183(12): 673-678.
24. Kim TH, Kim DY, Park JW, Kim YI, Kim SH, Park HS, Lee WJ, Park SJ, Hong EK, Kim CM. Three-dimensional conformal radiotherapy of unresectable hepatocellular carcinoma patients for whom transcatheter arterial chemoembolization was ineffective or unsuitable. *Am J Clin Oncol.* 2006 Dec; 29(6): 568-575.
25. Zhou ZH, Liu LM, Chen WW, Men ZQ, Lin JH, Chen Z, Zhang XJ, Jiang GL. Combined therapy of transcatheter arterial chemoembolisation and three-dimensional conformal radiotherapy for hepatocellular carcinoma. *Br J Radiol.* 2007 Mar; 80(951): 194-201.
26. Seo DD, Lee HC, Jang MK, Min HJ, Kim KM, Lim YS, Chung YH, Lee YS, Suh DJ, Ko GY, Lee YJ, Lee SG. Preoperative portal vein embolization and surgical resection in patients with hepatocellular carcinoma and small future liver remnant volume: Comparison with transarterial chemoembolization. *Ann Surg Oncol.* 2007 Dec; 14(12): 3501-3509.
27. Kamel IR, Liapi E, Reyes DK, Zahurak M, Bluemke DA, Geschwind JF. Unresectable hepatocellular carcinoma: Serial early vascular and cellular changes after transarterial chemoembolization as detected with MR imaging. *Radiology.* 2009 Feb; 250(2): 466-473.
28. Lo SS, Teh BS, Mayr NA, Olencki TE, Wang JZ, Grecula JC, Lu JJ, Timmerman RD. Stereotactic body radiation therapy for oligometastases. *Discov Med.* 2010 Sep; 10(52): 247-254.
29. Ben-Josef E, Lawrence TS. Using a bigger hammer: The role of stereotactic body radiotherapy in the management of oligometastases. *J Clin Oncol.* 2009 Apr 1; 27(10): 1537-1539.
30. Christodouleas JP, Marks LB. Analysis of patients with oligometastases undergoing two or more curative-intent stereotactic radiotherapy courses: In regard to milano et al. (int J radiat oncol biol phys 2009;73:832-837). *Int J Radiat Oncol Biol Phys.* 2009 Aug 1; 74(5): 1628; author reply 1628-9.
31. Kavanagh BD, McGarry RC, Timmerman RD. Extracranial radiosurgery (stereotactic body radiation therapy) for oligometastases. *Semin Radiat Oncol.* 2006 Apr; 16(2): 77-84.
32. Kelloff GJ, Krohn KA, Larson SM, et al. The progress and promise of molecular imaging probes in oncologic drug development. Clin Cancer Res. Nov 15 2005;11(22):7967-7985.
33. Shankar LK, Hoffman JM, Bacharach S, et al. Consensus recommendations for the use of 18F-FDG PET as an indicator of therapeutic response in patients in National Cancer Institute Trials. J Nucl Med. Jun 2006;47(6):1059-1066.
34. Turlakow A, Larson SM, Coakley F, et al. Local detection of prostate cancer by positron emission tomography with 2-fluorodeoxyglucose: comparison of filtered back projection and iterative reconstruction with segmented attenuation correction. Q J Nucl Med. Sep 2001;45(3):235-244.
35. Hofer C, Laubenbacher C, Block T, Breul J, Hartung R, Schwaiger M. Fluorine-18-fluorodeoxyglucose positron emission tomography is useless for the detection of local recurrence after radical prostatectomy. European urology. 1999;36(1):31-35.
36. Nunez R, Macapinlac HA, Yeung HW, et al. Combined 18F-FDG and 11C-methionine PET scans in patients with newly progressive metastatic prostate cancer. J Nucl Med. Jan 2002;43(1):46-55.
37. Shreve PD, Grossman HB, Gross MD, Wahl RL. Metastatic prostate cancer: initial findings of PET with 2-deoxy-2-[F-18]fluoro-D-glucose. Radiology. Jun 1996;199(3):751-756.

38. Hricak H, Choyke PL, Eberhardt SC, Leibel SA, Scardino PT. Imaging prostate cancer: a multidisciplinary perspective. Radiology. Apr 2007;243(1):28-53.

39. Lawrentschuk N, Davis ID, Bolton DM, Scott AM. Positron emission tomography and molecular imaging of the prostate: an update. BJU international. May 2006;97(5):923-931.
40. Scattoni V, Picchio M, Suardi N, et al. Detection of lymph-node metastases with integrated [11C]choline PET/CT in patients with PSA failure after radical retropubic prostatectomy: results confirmed by open pelvic-retroperitoneal lymphadenectomy. European urology. Aug 2007;52(2):423-429.
41. de Jong IJ, Pruim J, Elsinga PH, Vaalburg W, Mensink HJ. 11C-choline positron emission tomography for the evaluation after treatment of localized prostate cancer. European urology. Jul 2003;44(1):32-38; discussion 38-39.
42. Picchio M, Messa C, Landoni C, et al. Value of [11C]choline-positron emission tomography for re-staging prostate cancer: a comparison with [18F]fluorodeoxyglucose-positron emission tomography. The Journal of urology. Apr 2003;169(4):1337-1340.
43. Morris MJ, Scher HI. (11)C-acetate PET imaging in prostate cancer. European journal of nuclear medicine and molecular imaging. Feb 2007;34(2):181-184.
44. Even-Sapir E, Metser U, Mishani E, Lievshitz G, Lerman H, Leibovitch I. The detection of bone metastases in patients with high-risk prostate cancer: 99mTc-MDP Planar bone scintigraphy, single- and multi-field-of-view SPECT, 18F-fluoride PET, and 18F-fluoride PET/CT. J Nucl Med. Feb 2006;47(2):287-297.
45. Even-Sapir E, Mishani E, Flusser G, Metser U. 18F-Fluoride positron emission tomography and positron emission tomography/computed tomography. Semin Nucl Med. Nov 2007;37(6):462-469.
46. Beattie BJ, Smith-Jones PM, Jhanwar YS, et al. Pharmacokinetic assessment of the uptake of 16beta-18F-fluoro-5alpha-dihydrotestosterone (FDHT) in prostate tumors as measured by PET. J Nucl Med. Feb 2011;51(2):183-192.
47. Schuster DM, Savir-Baruch B, Nieh PT, et al. Detection of recurrent prostate carcinoma with anti-1-amino-3-18F-fluorocyclobutane-1-carboxylic acid PET/CT and 111In-capromab pendetide SPECT/CT. Radiology. Jun 2011;259(3):852-861.
48. Schuster DM, Votaw JR, Nieh PT, et al. Initial experience with the radiotracer anti-1-amino-3-18F-fluorocyclobutane-1-carboxylic acid with PET/CT in prostate carcinoma. J Nucl Med. Jan 2007;48(1):56-63.
49. Greco C, Cascini GL, Tamburrini O. Is there a role for positron emission tomography imaging in the early evaluation of prostate cancer relapse? Prostate cancer and prostatic diseases. 2008;11(2):121-128.
50. Bostwick DG, Pacelli A, Blute M, Roche P, Murphy GP. Prostate specific membrane antigen expression in prostatic intraepithelial neoplasia and adenocarcinoma: a study of 184 cases. Cancer. Jun 1 1998;82(11):2256-2261.
51. Chang SS, Reuter VE, Heston WD, Gaudin PB. Comparison of anti-prostate-specific membrane antigen antibodies and other immunomarkers in metastatic prostate carcinoma. Urology. Jun 2001;57(6):1179-1183.
52. Sweat SD, Pacelli A, Murphy GP, Bostwick DG. Prostate-specific membrane antigen expression is greatest in prostate adenocarcinoma and lymph node metastases. Urology. Oct 1998;52(4):637-640.
53. Wright GL, Jr., Grob BM, Haley C, et al. Upregulation of prostate-specific membrane antigen after androgen-deprivation therapy. Urology. Aug 1996;48(2):326-334.
54. Perner S, Hofer MD, Kim R, et al. Prostate-specific membrane antigen expression as a predictor of prostate cancer progression. Human pathology. May 2007;38(5):696-701.
55. Ross JS, Sheehan CE, Fisher HA, et al. Correlation of primary tumor prostate-specific membrane antigen expression with disease recurrence in prostate cancer. Clin Cancer Res. Dec 15 2003;9(17):6357-6362.
56. Thomas CT, Bradshaw PT, Pollock BH, et al. Indium-111-capromab pendetide radioimmunoscintigraphy and prognosis for durable biochemical response to salvage radiation therapy in men after failed prostatectomy. J Clin Oncol. May 1 2003;21(9):1715-1721.
57. Wilkinson S, Chodak G. The role of 111indium-capromab pendetide imaging for assessing biochemical failure after radical prostatectomy. The Journal of urology. Jul 2004;172(1):133-136.

58. Barinka C, Rovenska M, Mlcochova P, et al. Structural insight into the pharmacophore pocket of human glutamate carboxypeptidase II. Journal of medicinal chemistry. Jul 12 2007;50(14):3267-3273.
59. Barinka C, Starkova J, Konvalinka J, Lubkowski J. A high-resolution structure of ligand-free human glutamate carboxypeptidase II. Acta crystallographica. Mar 1 2007;63(Pt 3):150-153.
60. Foss CA, Mease RC, Fan H, et al. Radiolabeled small-molecule ligands for prostate-specific membrane antigen: in vivo imaging in experimental models of prostate cancer. Clin Cancer Res. Jun 1 2005;11(11):4022-4028.
61. Mease RC, Dusich CL, Foss CA, et al. N-[N-[(S)-1,3-Dicarboxypropyl]carbamoyl]-4-[18F]fluorobenzyl-L-cysteine, [18F]DCFBC: a new imaging probe for prostate cancer. Clin Cancer Res. May 15 2008;14(10):3036-3043.
62. Cho SY, Gage KL, Mease RC, et al. Biodistribution, Tumor Detection and Radiation Dosimetry of N-[N-[(S)-1,3-dicarboxypropyl]carbamoyl]-4-18F-fluorobenzyl-L-cysteine (18F-DCFBC), a Low Molecular Weight Inhibitor of PSMA, in Patients with Metastatic Prostate Cancer. J Nucl Med. 2012 Dec;53(12):1883-91.
63. Szabo Z, Mena E, Rowe SP, et al. Initial Evaluation of [(18)F]DCFPyL for Prostate-Specific Membrane Antigen (PSMA)-Targeted PET Imaging of Prostate Cancer. Mol Imaging Biol. 2015 Aug;17(4):565-74.
64 Chiang, A. C. & Massague, J. Molecular basis of metastasis. *N Engl J Med* **359**, 2814-2823, doi:10.1056/NEJMra0805239359/26/2814 [pii] (2008).
65 Kim, M. Y. *et al.* Tumor self-seeding by circulating cancer cells. *Cell* **139**, 1315-1326, doi:10.1016/j.cell.2009.11.025S0092-8674(09)01437-8 [pii] (2009).
66 Comen, E., Norton, L. & Massague, J. Clinical implications of cancer self-seeding. *Nat Rev Clin Oncol* **8**, 369-377, doi:10.1038/nrclinonc.2011.64nrclinonc.2011.64 [pii] (2011).
67 Gundem, G. *et al.* The evolutionary history of lethal metastatic prostate cancer. *Nature* **520**, 353-357, doi:10.1038/nature14347nature14347 [pii] (2015).
68 Marrinucci, D. *et al.* Fluid biopsy in patients with metastatic prostate, pancreatic and breast cancers. *Phys Biol* **9**, 016003, doi:10.1088/1478-3975/9/1/016003S1478-3975(12)97631-6 [pii] (2012).
69 Newman, A. M. *et al.* An ultrasensitive method for quantitating circulating tumor DNA with broad patient coverage. *Nature medicine*, doi:10.1038/nm.3519nm.3519 [pii] (2014).
70 Robins, H. S. *et al.* Comprehensive assessment of T-cell receptor beta-chain diversity in alphabeta T cells. *Blood* **114**, 4099-4107, doi:10.1182/blood-2009-04-217604blood-2009-04-217604 [pii] (2009).
71 Cha, E. *et al.* Improved survival with T cell clonotype stability after anti-CTLA-4 treatment in cancer patients. *Sci Transl Med* 6, 238ra270, doi:10.1126/scitranslmed.30082116/238/238ra70 [pii] (2014).
72 Keizman D, Zahurak M, Sinibaldi V, et. al. Lenalidomide in nonmetastatic biochemically relapsed prostate cancer: results of a phase I/II double-blinded, randomized study. Clin Cancer Res. 2010 Nov 1;16(21):5269-76.
73. Pound C, Partin A, Eisenberger M, Chan DW, Pearson JD, Walsh PC. Natural history of progression after PSA elevation following radical prostatectomy. JAMA 1999;281:1591–7.
74. Antonarakis ES, Trock BJ, Feng Z, et al. The natural history of metastatic progression in men with PSA-recurrent prostate cancer after radical prostatectomy: 25-year follow-up. J Clin Oncol 2009;27: 15s, (suppl; abstr 5008.
75. Scher HI, Halabi S, Tannock I, et. al. Design and End Points of Clinical Trials for Patients With Progressive Prostate Cancer and Castrate Levels of Testosterone: Recommendations of the Prostate Cancer Clinical Trials Working Group. J Clin Oncol. 2008 Mar 1;26(7):1148-59.
76. Decaestecker K, De Meerleer G, Lambert B, *et al.* Repeated stereotactic body radiotherapy for oligometastatic prostate cancer recurrence. Radiat Oncol. 2014 Jun 12;9:135.
77. Vickers AJ. How to randomize. J Soc Integr Oncol 2006;4:194-8.
78. Zeng, J., Harris, T. J., Lim, M., Drake, C. G. & Tran, P. T. Immune modulation and stereotactic radiation: improving local and abscopal responses. *Biomed Res Int* **2013**, 658126, doi:10.1155/2013/658126 (2013).
79. Pritchard CC, Mateo JM, Walsh MF, et al. Inherited DNA-Repair Gene Mutations in Men with Metastatic Prostate Cancer. N Engl J Med 2016; 375:443-453

80. SEER Stat Fact Sheets: Colon and Rectum Cancer. Published November 2015. Updated April 2016. Accessed August 7 2016. http://seer.cancer.gov/statfacts/html/colorect.html
81. Color Genomics. (2016). *Color Genomics Democratizes Access to Clinical-Grade Genetic Testing Worldwide*. [Press release]. Retrieved from http://www.prnewswire.com/news-releases/color-genomics-democratizes-access-to-clinical-grade-genetic-testing-worldwide-300248913.html

APPENDIX I: Brief Pain Inventory Form

STUDY ID# ____________ HOSPITAL # ____________

DO NOT WRITE ABOVE THIS LINE

Brief Pain Inventory (Short Form)

Date: _____/_____ /_____ Time: _____

Name: ______________________ ______________________ ______________________

Last First Middle Initial

1. Throughout our lives, most of us have had pain from time to time (such as minor headaches, sprains, and toothaches). Have you had pain other than these every-day kinds of pain today?

   1. Yes 2. No

2. On the diagram, shade in the areas where you feel pain. Put an X on the area that hurts the most.

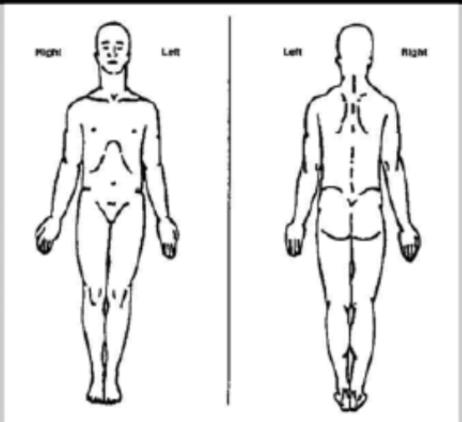

3. Please rate your pain by circling the one number that best describes your pain at its worst in the last 24 hours.

0	1	2	3	4	5	6	7	8	9	10
No Pain										Pain as bad as you can imagine

4. Please rate your pain by circling the one nuimber that best describes your pain at its least in the last 24 hours.

0	1	2	3	4	5	6	7	8	9	10
No Pain										Pain as bad as you can imagine

5. Please rate your pain by circling the one number that best describes your pain on the average.

0	1	2	3	4	5	6	7	8	9	10
No Pain										Pain as bad as you can imagine

6. Please rate your pain by circling the one number that tells how much pain you have right now.

0	1	2	3	4	5	6	7	8	9	10
No Pain										Pain as bad as you can imagine

7. What treatments or medications are you receiving for your pain?

8. In the last 24 hours, how much relief have pain treatments or medications provided? Please circle the one percentage that most shows how much relief you have received.

0% No Relief	10%	20%	30%	40%	50%	60%	70%	80%	90%	100% Complete Relief

9. Circle the one number that describes how, during the past 24 hours, pain has interfered with your:

A. General Activity

0 Does not Interfere	1	2	3	4	5	6	7	8	9	10 Completely Interferes

B. Mood

0 Does not Interfere	1	2	3	4	5	6	7	8	9	10 Completely Interferes

C. Walking Ability

0 Does not Interfere	1	2	3	4	5	6	7	8	9	10 Completely Interferes

D. Normal Work (includes both work outside the home and housework)

0 Does not Interfere	1	2	3	4	5	6	7	8	9	10 Completely Interferes

E. Relations with other people

0 Does not Interfere	1	2	3	4	5	6	7	8	9	10 Completely Interferes

F. Sleep

0 Does not Interfere	1	2	3	4	5	6	7	8	9	10 Completely Interferes

G. Enjoyment of life

0 Does not Interfere	1	2	3	4	5	6	7	8	9	10 Completely Interferes

Copyright 1991 Charles S. Cleeland, PhD
Pain Research Group
All rights reserved.
Used by permission.

APPENDIX II: Performance Status Criteria

ECOG Performance Status Scale		Karnofsky Performance Scale	
Grade	Descriptions	Percent	Description
0	Normal activity. Fully active, able to carry on all pre-disease performance without restriction.	100	Normal, no complaints, no evidence of disease.
		90	Able to carry on normal activity; minor signs or symptoms of disease.
1	Symptoms, but ambulatory. Restricted in physically strenuous activity, but ambulatory and able to carry out work of a light or sedentary nature (e.g., light housework, office work).	80	Normal activity with effort; some signs or symptoms of disease.
		70	Cares for self, unable to carry on normal activity or to do active work.
2	In bed <50% of the time. Ambulatory and capable of all self-care, but unable to carry out any work activities. Up and about more than 50% of waking hours.	60	Requires occasional assistance, but is able to care for most of his/her needs.
		50	Requires considerable assistance and frequent medical care.
3	In bed >50% of the time. Capable of only limited self-care, confined to bed or chair more than 50% of waking hours.	40	Disabled, requires special care and assistance.
		30	Severely disabled, hospitalization indicated. Death not imminent.
4	100% bedridden. Completely disabled. Cannot carry on any self-care. Totally confined to bed or chair.	20	Very sick, hospitalization indicated. Death not imminent.
		10	Moribund, fatal processes progressing rapidly.
5	Dead.	0	Dead.

APPENDIX III: CAPP-Seq Sample Collection & Processing

Diehn Lab Blood Processing Protocol (version 122314)

❖ **Materials** (check to make sure you have all of it before you start)

- Filtered P2000 pipette tips
- P-2000 pipette
- 2.0-mL Eppendorf tubes
- Patient's blood - usually 30 mL in 3 "purple top" tubes (EDTA) per time point. Blood should be kept on ice or in refrigerator after drawing and processed as soon as possible to minimize lysis of WBCs and release of cellular genomic DNA into plasma

❖ **Methods**

1. Spin samples in the clinical centrifuge using the settings: 3,500rpm, 10min, 4C
2. While you wait, label tubes
3. After spinning, carefully remove lavender top tubes from centrifuge. Do not disturb the separated plasma and cell-free whole blood
    i. Tip: It helps to put all the tubes into one holder and carefully carry the holder to the hood
4. Using a filtered tip and p-2000 pipet aliquot ~1.8 mL clear plasma (not all the way to the top since tops of tubes tend to pop open upon freezing if filled all the way) into a 2.0 mL Eppendorf tube. Repeat until you have aliquoted the plasma from all the purple-top tubes into 2.0 mL Eppendorf tubes. With the tubes that have only the buffy coat and RBC remaining, mix the buffy coat and cell-free whole blood using a pipette tip and aliquot ~1.8 mL into a 2.0 mL Eppendorf tube. Repeat so you have a second 2.0 mL Eppendorf tube containing the buffy coat and RBC mixed together.
5. Put Eppendorf tubes into -80C freezer.
6. Write down **# of plasma tubes stored, box number, date of blood draw, time of storage** on the top of patients' requisition forms.
7. Enter the blood draw information into REDCap database.

APPENDIX IV: EPIC-HD CTC Sample Collection & Processing

EPIC SCIENCES

EPIC SCIENCES, INC.
9381 Judicial Dr, Suite 200
San Diego, CA 92121

CTC Sample Collection in Streck Cell-Free DNA BCT Tubes

IMPORTANT: The first 5 mL of blood collected from the fresh venipuncture **cannot** be used for the collection into the Streck tubes due to possibility of contaminating epithelial cells during venipuncture. **Please ensure that at least one blood tube of 5 mL or more is collected prior to collection of the CTC sample to avoid adversely affecting the test results.**

Prevention of Backflow:

Since Streck Cell-Free DNA BCT tubes contain chemical additives, it is important to avoid possible backflow from the tube. To guard against backflow, observe the following precautions:

- Keep patient's arm in the downward position during the collection procedure.
- Hold the tube with the stopper uppermost.
- Release tourniquet once the blood starts to flow into the tube, or within 2 minutes of application.
- Tube contents should not touch stopper or the end of the needle during the collection procedure.

Blood Collection Instructions:

**Schedule courier for same-day sample pic-up prior to collection

1. Confirm blood tube is not expired. Expired tubes should not be used for blood collection.
2. Draw whole blood sample into 10 mL Streck Cell-Free DNA BCT tube (*see note regarding prevention of backflow). Fill tube until blood flow stops. NOTE: Epic requires a minimum of 4mL blood per sample, but a full 10 mL tube of blood should be provided when possible.
3. Remove tube from adapter and immediately mix by gentle inversion 8 to 10 times. Tube inversion prevents clotting. Inadequate or delayed mixing may result in inaccurate test results.
4. Label the tube with subject's identification and date and time of blood draw.
5. Keep sample at ***room temperature*** and ***ship on day of collection.***

Sample Shipment Instructions:

Samples are processed the same day as receipt. Priority is given to the samples that had notification the day prior. Epic is able to process samples up to 96 hours after the initial blood draw, but it is preferable to process the blood before the 48 hour mark for optimal CTC retention and data integrity purposes.

EPIC SCIENCES

EPIC SCIENCES, INC.
9381 Judicial Dr, Suite 200
San Diego, CA 92121

The total number of slides created is dependent upon the patient's WBC count and amount of blood provided. Each tube should be labeled with the Study and Subject ID. Each sample sent should be accompanied by a requisition form, filled out by the site, listing the study ID, subject ID, draw date, draw time, and time point (if applicable). To avoid weekend delays, mark Saturday delivery if shipping on Friday.

Samples should be sent overnight in ambient shippers on the day the blood sample is drawn.

Epic Sciences receives samples Monday through Saturday from 8:00 a.m. to 5:00 p.m., excluding U.S. holidays. Incoming sample notifications should be sent during normal business hours on the day of shipment by email to partners@epicsciences.com or by fax at 858-356-5852. For samples that will be processed at LabCorp Belgium, please send the sample notification email to partners.eu@epicsciences.com. For samples that will be processed at LabCorp Singapore, please send the sample notification email to LabCorp.Singapore@epicsciences.com.

This email/fax should include:

1) Study ID
2) Subject ID (and timepoint if applicable)
3) Tracking Number
4) Number of samples being shipped
5) Date and time of each blood sample draw

APPENDIX V: ImmunoSEQ Sample Collection & Processing

immunoSEQ® Service *amplify* **discovery™**

Human Sample Preparation Guidelines

Sample Types Accepted
- Sorted T and B cells
- Peripheral blood mononucleated cells (PBMCs)
- Whole blood
- Tissue (including Fresh Frozen [FF] or formalin-fixed, paraffin-embedded [FFPE] tissue)
- gDNA and cDNA

Assays available:
- T-cell receptor beta (TCRB)
- T-cell receptor alpha/delta (TCRA/D)
- T-cell receptor gamma (TCRG)
- IGH
- IG kappa/lambda (IGK/L)

cDNA GUIDELINES

- A minimum of 150 ng of RNA is recommended as starting material for the reverse transcription step
- Adaptive targets 10-fold sequencing coverage of each TCR or BCR template
- Using cDNA as an input source will limit available applications

TCR IMMUNOSEQ ASSAY SAMPLE GUIDELINES

Sample type	Resolution			
	Survey	Deep	Ultra Deep	Max Depth
Sorted T cells	60,000 cells[a] 1 μg DNA 50 μL vol.	200,000 cells 3 μg DNA 125 μL vol.	800,000 cells 12 μg DNA 400 μL vol.	4,000,000 cells 48 μg DNA 700 μL vol.
PBMCs	120,000 cells 2 μg DNA 50 μL vol.	400,000 cells 6 μg DNA 125 μL vol.	1,600,000 cells 24 μg DNA 400 μL vol.	8,000,000 cells 96 μg DNA 700 μL vol.
Whole blood	2 mL blood 4 μg DNA	4 mL blood 12 μg DNA	10 mL blood 48 μg DNA	Contact Technical Support
Lymphoid tissue[b]	25 microns FFPE 10 mg FF tissue 1 μg DNA	3 μg DNA	Contact Technical Support	
Non-lymphoid tissue	25 microns FFPE 10 mg FF tissue 3 μg DNA	9 μg DNA	Not recommended	

[a]1000 cells is the absolute minimum number of cells accepted.
[b] Deep resolution is recommended for lymphoid tissue samples.

NOTE: In order to sequence only rearranged TCRG receptors originating from gamma/delta T cells in PBMCs and tissue samples, a cell sort must be performed prior to receipt of samples by Adaptive Biotechnologies. Unsorted cells will result in the amplification of rearranged TCRG receptors from both gamma/delta and alpha/beta T cells.

BCR IMMUNOSEQ ASSAY SAMPLE GUIDELINES

Sample type	Resolution			
	Survey	Deep	Ultra Deep	Max Depth
Sorted B cells	60,000 cells[a] 1 μg DNA 50 μL vol.	200,000 cells 3 μg DNA 125 μL vol.	800,000 cells 12 μg DNA 400 μL vol.	4,000,000 cells 48 μg DNA 700 μL vol.
PBMCs/bone marrow[b]	600,000 cells 4 μg DNA 50 μL vol.	2,000,000 cells 12 μg DNA 125 μL vol.	Contact Technical Support	
Lymphoid tissue	10 mg FF tissue 1 μg DNA	3 μg DNA		
Non-lymphoid tissue	10 mg FF tissue 3 μg DNA	9 μg DNA	Not recommended	

[a]1000 cells is the absolute minimum number of cells accepted.
[b]B cells represent a small fraction of the total PBMC population; this resolution may not be appropriate for all projects.

DESCRIPTION OF PROFILING RESOLUTIONS: SURVEY VS. DEEP

Resolution	Considerations for choosing resolution
Survey	• Clonal samples<br>• Samples with low numbers of T or B cells (<100,000 estimated T or B cells cells)<br>• Samples derived from most non-lymphoid tissues
Deep	• Studying the peripheral immune repertoire (e.g. whole blood, peripheral blood mononuclear cells [PBMCs], or lymphoid tissue)<br>• Samples requiring greater sensitivity (detection of rare clones)<br>• Experiments assessing a broader range of the repertoire

amplify **discovery**™

RECOMMENDATIONS FOR SAMPLE PREPARATION

Isolating DNA from different sample types

Sorted cells

- Sorting fixed cells into HEPES buffer (PBS with 2% FBS and 0.025M HEPES) can boost the DNA yield from the cell pellets
- When preparing fixed cells for fluorescence-activated cell sorting (FACS), a concentration of 0.5%–2.0% PFA is recommended. Higher concentrations of PFA can fragment the DNA, which will result in reduced PCR amplification efficiency
- Cells should arrive in no more than 200 µL of buffer

Tissue

- A tissue homogenizer with homogenization buffer is recommended for disruption of fresh or frozen tissue samples
- Example kit for DNA extraction:
  - Qiagen DNeasy™ Blood & Tissue Kit (Mini Spin Columns)
  - Qiagen QIAamp™ DNA FFPE Tissue Kit

Blood, PBMCs, or bone marrow

- ACD or EDTA is recommended as anticoagulant for whole blood or bone marrow collection
- Sodium heparin and sodium citrate are compatible with the immunoSEQ Assay. However, excessive amounts of sodium heparin can inhibit PCR
- Roughly 50% of cells frozen in DMSO will lyse during the thawing process. To recover all DNA do not centrifuge the sample after thawing. Instead, extract DNA from the entire thawed sample
- Possible extraction kits:
  - Qiagen DNeasy™ Blood & Tissue Kit (Mini Spin Columns): Bone marrow and <1 mL blood
  - Qiagen QIAamp™ DNA Blood Maxi Kit: 1–10 mL blood

Shipping Samples

- Cells and tissue: 1.5 or 2.0 mL Eppendorf tubes/ cryotubes (snap top or screw cap)
- Whole blood: Vacutainer ACD or EDTA tube, completely filled
- gDNA or cDNA: Uniquely barcoded sample containers will be provided by Adaptive Biotechnologies.

Quality of input DNA

Once DNA is isolated, quantification using a spectrophotometer or comparable method is highly recommended. For optimal results the absorbance ratios of DNA samples should be:

- A260/280 = 1.8–2.0
- A260/230 = 2.0–2.2

Potential PCR inhibitors

Sample source(s) containing any of the following may inhibit PCR steps used in the immunoSEQ Assay:

- **Heparin, EDTA,** common anticoagulants in blood and bone marrow samples
- **Melanin,** common to skin and melanoma tissue samples
- **B5 Reagent,** commonly used for bone marrow storage
- **Collagen,** can be at high levels in some tissue samples
- **Myoglobin,** common to muscle tissue
- **Bacterial contamination** from all sample sources
- **Phenol, ethanol,** and **other organic contaminants** remaining after DNA extraction

For questions or Technical Support contact:
techsupport@adaptivebiotech.com, or (855) 466-8667

For Research Use Only. Not for use in diagnostic procedures.
Copyright © 2015 Adaptive Biotechnologies Corp. All rights reserved.
MRK-10003-01 AC

Adaptive biotechnologies®
adaptivebiotech.com

APPENDIX VI: RECTAL SWAB COLLECTION FOR MICROBIOME ANALYSIS

Reagents and Supplies Needed:

Regular flocked swab, plastic applicator, sterile in dry tube (Copan, catalog #552C)

Protocol:

1. Remove swab from plastic tube. Hold the swab by the end of the plastic handle and take care not to touch the cotton tip of the swab to any surfaces or with fingers.

2. Insert the swab through the rectal sphincter and approximately 3 cm into the anal canal. Rotate the swab and withdraw.

3. Place the swab back into the plastic tube.

The swab samples can be kept at 4°C or -20°C prior to transport to the Sfanos lab.

APPENDIX VII: COLOR SALIVA KIT SAMPLE COLLECTION

KIT INCLUDES:

Saliva Tube, Detailed Instructions, and Blue Cap.

Protocol:

1. In order to not delay results or ruin sample, the patient must wait 30 minutes if he has:
    a. Had any food or drink
    b. Brushed his teeth
    c. Used mouthwash
    d. Smoked or chewed gum

2. Remove tube from clear tray. Do not remove the plastic film from the lid attached to the funnel. It contains stabilizing liquid that will mix with the saliva later once you close the lid.

3. Spit into the funnel until the amount of saliva reaches the “fill to” line. This might take a few minutes (thinking of candy or rubbing your cheeks can speed up the process). Remember saliva bubbles do not count.

4. Once you have reached the “fill to” line, hold the tube upright with one hand, and close the funnel lid with the other hand by firming pushing until you hear a loud click. Do not be surprised when the fluid from the lid is released into the tube. Make sure it is closed tightly.

5. Take the small blue cap out of the clear tray. Holding the tube upright, unscrew the funnel to remove it completely, then screw the small cap onto the tube tightly.

6. Shake the tube for the 5 seconds, to mix the liquid and saliva together. This will preserve the sample across a wide range of temperatures.

7. Visit www.getcolor.Com/activate to link sample to color account. You will need the bar code found on saliva tube.

    IT IS ABSOLUTELY NECESSARY THAT YOU ACTIVATE YOUR KIT OR COLOR WILL NOT BE ABLE TO ANALYZE THE SAMPLE.

8. Place tube into the provided plastic bag, seal the bag, then place it back into the cardboard box. Seal the box and drop it off in any USPS mailbox. Postage has already been paid. Everything else can be thrown away, **as long as you have activated your kit successfully.**

    You will receive an email once the sample has been received by the lab and another email once results are ready.
