# Supplementary material for: Outcomes of Observation vs Stereotactic Ablative Radiation for Oligometastatic Prostate Cancer: The ORIOLE Phase 2 Randomized Clinical Trial
Source: JAMA Oncol. 2020 Mar 26;6(5):650–9. doi: 10.1001/jamaoncol.2020.0147 (PMC7225913; doi:10.1001/jamaoncol.2020.0147)
Supplement: Supplement 2. — eMethods. eResults. eFigure 1. Total gross tumor volume in patients treated with SABR was not different in patients with or without progression at 180 days eFigure 2. Baseline circulating tumor DNA concentration is not associated with outcome eTable 1. Treatment volume characteristics for SABR arm eTable 2. Distribution of lesions by treatment arm eTable 3. CTCAE v4.0 grade 1 adverse events eTable 4. CTCAE v4.0 grade 2 adverse events eTable 5. Genes fully or partially covered in prostate cancer focused CAPP-Seq selector eTable 6. Mutations identified by CAPP-Seq in baseline plasma samples eReferences. [file jamaoncol-6-650-s002.pdf]

Supplementary Online Content

Phillips R, Shi WY, Deek M, et al. Outcomes of observation vs stereotactic ablative radiation for oligometastatic prostate cancer: the ORIOLE phase 2 randomized clinical trial. *JAMA Oncol*. Published online March 26, 2020. doi:10.1001/jamaoncol.2020.0147

eMethods.

eResults.

eFigure 1. Total gross tumor volume in patients treated with SABR was not different in patients with or without progression at 180 days

eFigure 2. Baseline circulating tumor DNA concentration is not associated with outcome

eTable 1. Treatment volume characteristics for SABR arm

eTable 2. Distribution of lesions by treatment arm

eTable 3. CTCAE v4.0 grade 1 adverse events

eTable 4. CTCAE v4.0 grade 2 adverse events

eTable 5. Genes fully or partially covered in prostate cancer focused CAPP-Seq selector

eTable 6. Mutations identified by CAPP-Seq in baseline plasma samples

eReferences.

This supplementary material has been provided by the authors to give readers additional information about their work.

© 2020 Phillips et al. *JAMA Oncology.*

eMethods

Inclusion Criteria

PSA values at enrollment were ≥ 0.5 ng/mL but ≤ 50 ng/mL with testosterone ≥ 125 ng/dL and a PSA doubling time (PSADT) < 15 months. PSA doubling time was calculated using all available PSA values from time of relapse utilizing the Memorial Sloan Kettering Cancer Center PSA Doubling Time Calculator (https://www.mskcc.org/nomograms/prostate/psa_doubling_time). Patients also had leukocytes > 2,000/μL, an absolute neutrophil count >1,000/μL, and platelets >50,000/μL. Finally, patients were at least 18 years of age, had an Eastern Cooperative Oncology Group performance status of ≤ 2, and had capacity to understand and provide informed consent for treatment.

Exclusion Criteria

Patients were not eligible for enrollment if they had previously received more than three years of ADT, received ADT in the prior six months, or developed castration-resistant disease. Patients could not receive other investigational agents during participation in this trial or participate in concurrent treatment protocols. Patients who previously underwent PSMA-targeted PET/MRI or PET/CT within the prior six months were not eligible for enrollment if they possessed radiotracer-avid lesions not appreciable on conventional imaging. Suspected pulmonary and/or liver metastases greater > 10 mm in largest axis, spinal cord compression, and impending spinal cord compression were grounds for exclusion. Additional grounds for exclusion included serum creatinine > threefold the upper limit of normal, total bilirubin > threefold the upper limit of normal, transaminases > fivefold the upper limit of normal, or the inability to lie flat during or otherwise physically tolerate PET/CT, MRI, or SABR. Refusal to sign an informed consent document was also grounds for exclusion.

© 2020 Phillips et al. *JAMA Oncology*.

Follow up and Crossover

Participants were advised to continue routine follow-up visits every 3 months in the absence of progression. Participants who progressed at any point were evaluated and referred as appropriate for further therapy which could include additional SABR to new sites of metastatic disease. Participants on the observation arm were allowed to cross over at the time of progression or at day 180.

Sample Size Estimation/Power Analysis

The primary endpoint was rate of progression at 6-months. Historical data on this patient population indicated that >80% would show progression as defined above within a 6-month period without treatment, and thus this was the progression rate we expected in subjects in the control/observation arm (ref). We hypothesized that SBRT will be able to reduce the progression at 6-months by 50% (ref). A sample size using a 1:2 randomization scheme of 18 patients in the control group and 36 in the SBRT arm provided 85% power to detect a decrease relapse rate from 80% to 40% with a type I error = 0.05 using one-sided Fisher's exact test. Thus, we accrued a total of 54 patients. No interim analysis for futility was planned since the progression endpoint would not have been reached by a meaningful number of patients before full accrual.

Early Stopping Guidelines

This study monitored site-specific grade 4/5 toxicity in the SBRT arm. A proportion of grade 4/5 toxicity at specific sites convincingly exceeding 20% would trigger a study halt for a safety consultation. Specifically, we applied a Bayesian toxicity monitoring rule that would suspend the

© 2020 Phillips et al. *JAMA Oncology.*

enrollment if the posterior probability of toxicity being larger than 20% threshold is 75% or higher. The monitoring rule used Beta (0.5, 5.5) as prior distribution. This means that our prior guess of the proportion of toxicity was 8.3%, and there was 90% chance that this proportion was 0.04%-30.6%. The monitoring started from the first patient, and the decision rule for safety stopping was as follows:

Stop if:

**# grade 4/5 toxicity >=**	3	4	5	6	7	8	9	10
**Out of # patients**	3 - 5	6 - 10	11 - 14	15 - 18	19 - 23	24 - 27	28 - 32	33 - 36

The operating characteristics of the stopping rule are shown below and are based on 5000 simulations:

**True AE rate**	**% simulated trials declaring unsafe**	**Average sample size**
0.10	2.6	35.3
0.2	31.5	29.6
0.25	56.4	24.6
0.3	78.4	19.2
0.35	91.9	14.7

© 2020 Phillips et al. *JAMA Oncology*.

Randomization

A dynamic minimization algorithm from a web-based system was utilized; no pre-determined sequence was created for allocation to arm A (Observation) and B (SABR). Instead this dynamic system taking into account the stratification factors assigned the study participants in a 1:2 fashion to Observation: SABR. To further reduce the ability of care providers to guess the arm in which the patient is ultimately assigned, a “Probability of Study Arm Assignment” function was introduced to allow for a small, random chance that a patient could still be assigned to the under enrolled arm. There was a 15% chance a patient may be assigned to the study arm that had more patients. Another parameter, “Maximum Imbalance”, was given a value of 4 so that the imbalance between arms could not be more than a difference of 4 patients. Prior to randomization of study participants, simulations were run and allocations were consistently balanced for stratification factors and absolute patients numbers. The details and entirety of the code for this minimization algorithm will be provided upon request.

Correlative Study Procedures

On Day 1 after randomization, participants in both arms had blood collected for standard tests (CBC, serum chemistry, LDH, PSA, testosterone) as well as for T-cell receptor sequencing (ImmunoSEQ) and CAPP-Seq analysis. Participants in both arms also underwent rectal swab for gut microbiome analysis and cheek swab for Color Genomics determination of germline DNA repair mutations. Participants randomized to SABR had additional blood drawn for circulating tumor cell (CTC) enumeration and underwent ^{18}F-DCFPyL (PSMA) PET/CT if not already performed.

© 2020 Phillips et al. *JAMA Oncology.*

At Day 90, both arms were seen for history and physical and had blood drawn for standard labs and CAPP-Seq and ImmunoSEQ. At day 180, all participants were seen for history and physical, had blood drawn for standard labs and CAPP-Seq, and underwent CT chest/abdomen/pelvis and bone scan. Participants in the SABR arm also had blood drawn for CTC enumeration and underwent ^{18}F-DCFPyL PET/CT.

CAPP-Seq ctDNA analysis was performed as described previously[1] using a 303kb custom panel covering all or a subset of exons of 299 genes recurrently somatically altered in prostate cancer (Supplemental Table X). Briefly, paired-end sequencing was performed on the Illumina HiSeq4000 platform using custom adapters for sample multiplexing and molecular barcoding. Sequencing reads were mapped to hg19 followed by removal of PCR duplicates and technical artifacts. Cell-free DNA (cfDNA) was extracted from 6.6-7.9 mL of plasma using the QiaAmp Circulating Nucleic Acid Kit according to manufacturer's instructions. cfDNA and paired leukocyte DNA were sequenced to median unique depths of 6,432 and 5,213, respectively for participant samples, and 5,254 and 3,374 for healthy controls. All paired leukocyte DNA samples were used as a background reference to identify recurrent technical artifacts. Somatic mutations were identified by paired analysis of pretreatment plasma and leukocyte DNA to eliminate mutations due to clonal hematopoiesis. Variant filtering criteria for the prostate cancer CAPP-Seq selector were optimized on control samples to ensure high specificity prior to application to participant samples. These filters were tuned for 100% specificity for plasma genotyping when testing 18 non-cancer, male control subjects.

Additional Statistical Methods

Rstudio (Rstudio Inc, Boston, MA) was used for the following calculations using Fisher's exact test: composite progression at 6 months, biochemical progression at 6 months, composite

© 2020 Phillips et al. *JAMA Oncology.*

progression at 6 months for total vs subtotal consolidation with SABR, and new metastatic lesions at 6 months for total vs subtotal consolidation with SABR. Executable code for these calculations is available in the separate Supplementary Statistical Methods document.

GraphPad Prism 8 (GraphPad Software Inc, San Diego, CA) was used for the following calculations: survival curves (Kaplan-Meier method with p-values calculated using log-rank test), brief pain inventory response comparisons (Holm-Sidak method for multiple t-tests).

Clonality and differential abundance were determined using the cloud-based Adaptive Biosciences ImmunoSEQ Platform. Parameters for differential abundance determination as well as analysis code executable in Rstudio for plotting differential abundance and clonality are included in the separate Supplementary Statistical Methods document. Clustering analysis was performed as previously published by Dash et al.[2]

CAPP-Seq analysis was performed using previously published methods.

© 2020 Phillips et al. *JAMA Oncology.*

eResults

Total gross tumor volume was not significantly different in patients treated with SABR who progressed at 180 days and those who were progression-free (eFigure 1; P = 0.10 by Mann Whitney test).

No difference in node-only vs bone-involved disease was identified between the observation and SABR arms (P = 0.77 by Fisher's exact test). To evaluate the effect of distribution of metastasis on progression-free survival, comparisons between node-only and bone-involved patients were performed using the Kaplan-Meier method and no difference in progression-free survival was seen in either the observation (P = 0.39 by Log-rank) or SABR arms (P = 0.96 by Log-rank).

A strength of our study design was that baseline PSMA-PET/CT images were not used to inform the treatment plan and were evaluated in a blinded fashion. Ninety evaluable lesions were identified in radiation treatment fields within the SABR arm. Local control of evaluable SABR-treated lesions was 98.9% at 6 months, as 89 out of 90 evaluable SABR-treated lesions exhibited stable disease (SD), partial response (PR) or complete response (CR) at 6 months. To be more specific, of 90 evaluable lesions treated with SABR, 25 (27.8%) achieved CR, 39 (43.3%) PR, 25 (27.8%) SD, and 1 (1.1%) PD at 6 months. Additional 49 evaluable lesions were identified in the SABR arm which were not included in radiation treatment fields. By size, of 49 lesions untreated with SABR, 4 (8.2%) demonstrated CR, 3 (6.1%) PR, 19 (38.8%) SD, and 23 (46.9%) PD at 6 months.

The relevance of changes in SUVmax on PSMA-PET after treatment with SABR are not well understood. In our cohort, of 109 PET-avid lesions treated with SABR, 27 (24.8%) completely

© 2020 Phillips et al. *JAMA Oncology.*

resolved, 50 (45.9%) showed an SUVmax decrease of at least 30%, 22 (20.2%) experienced less than 30% decrease and less than 20% increase, and 10 (9.2%) increased by at least 20%. Of 54 PET-avid untreated lesions, 4 (7.4%) completely resolved, 3 (5.6%) decreased at least 30%, 14 (25.9%) experienced less than 30% decrease and less than 20% increase, and 33 (61.1%) increased at least 20%.

The total number of lesions present on baseline PSMA-PET was also associated with clinical outcomes. Progression at 180 days by composite endpoint occurred in 0 of 20 (0%) participants with 1-3 PET-avid lesions at baseline as compared to 7 of 15 (46.7%) men with 4 or more lesions (p-value = 0.001 by Fisher's exact test). Similarly, newly detectable lesions were seen on PSMA-PET at 180 days in 2 of 20 (10.0%) men with 1-3 baseline lesions and 11 of 15 (73.3%) men with 4 or more baseline lesions (p-value = 0.002 by Fisher's exact test).

Contingency analyses were performed to compare new metastases at 6 months in patients receiving total consolidation by SABR whose lesions all demonstrated CR/PR compared to those with at least one lesion demonstrating SD/PD at 180 days. No differences were seen using size (P = 0.53 by Fisher's exact test), SUV (P = 0.22 by Fisher's exact test), or a combined threshold (P = 0.55 by Fisher's exact test) for defining lesion response.

© 2020 Phillips et al. *JAMA Oncology.*

Executable Code from Analysis

1. Fisher's Exact Test Calcuation using Rstudio

```
knitr::opts_chunk$set(echo = TRUE, eval=F)
rm(list=ls())


inline_hook <- function(x) {
  if (is.numeric(x)) {
    format(x, digits = 5)
  } else x
}
knitr::knit_hooks$set(inline = inline_hook)


## load library
library(readxl)
library(dplyr)
library(tidyr)
library(tidyverse)
library(survival)
library(survminer)
library(kableExtra)
library(rmarkdown)


## load updated datasets
summary <- read_excel("ORIOLE Master Sheet 010919.xlsx",sheet=1)%>%as.data.frame()
imaging.summary <- read_excel("ORIOLE Master Sheet 010919.xlsx",sheet=2)%>%as.data.fr
```

© 2020 Phillips et al. *JAMA Oncology*.

```
ame()
immuno.seq <- read_excel("ORIOLE Master Sheet 010919.xlsx",sheet=3)%>%as.data.frame()
ctc.newmets <- read_excel("ORIOLE Master Sheet 010919.xlsx",sheet=4)%>%as.data.frame()
ctcs <- read_excel("ORIOLE Master Sheet 010919.xlsx",sheet=5)%>%as.data.frame()
imaging <- read_excel("ORIOLE Master Sheet 010919.xlsx",sheet=6)%>%as.data.frame()
lesion.summary <- read_excel("ORIOLE Master Sheet 010919.xlsx",sheet=7)%>%as.data.fra
me()
patient.char <- read_excel("ORIOLE Master Sheet 010919.xlsx",sheet=8)%>%as.data.frame()

## tidy up datasets
valid.summary <- summary[c(2:19,21:56),]
valid.immuno.seq = immuno.seq[2:42,]
```

2. Composite progression at 6 months, SABR vs observation

```
## compute Agresti-Coull 95% confidence interval for proportion
agresti.ci = function(x = x, n = n){
  pseudo.x = x+2
  pseudo.n = n+4
  pseudo.p = pseudo.x/pseudo.n
  ci = round(pseudo.p + c(-1,1)*qnorm(0.975)*sqrt(pseudo.p*(1-pseudo.p)/pseudo.n),3)*100
  print(ci)
}

## generate 2x2 table
table.prog.arm = table(arm = valid.summary$Arm, prog6mo = factor(valid.summary$`Any progr
```

© 2020 Phillips et al. *JAMA Oncology.*

```
ession at 180 days?`, levels = c(0, 1), labels = c("Not progressed","Progressed")))


## compute 95% CI

agresti.ci(7,36) # SABR

agresti.ci(11,18) # OBS


## fisher test

fisher.test(table.prog.arm)
```

3. PSA progression at 6 months, SABR vs observation

```
## generate 2x2 table

table.psa.arm = table(arm = valid.summary$Arm, biochem6mo = factor(valid.summary$`Bioche
mical progression at 180 days?`, levels = c(0, 1), labels = c("Not progressed", "Progressed")))


## compute 95% CI

agresti.ci(4,36) # SABR

agresti.ci(9,18) # OBS


## fisher test

fisher.test(table.psa.arm)
```

© 2020 Phillips et al. *JAMA Oncology*.

4. Composite progression at 6 months, SABR arm only, no untreated lesions vs at least 1 untreated lesion

```
# 0: no untreated, 1: > 0 untreated
imaging.summary$untreated.lesion = ifelse(imaging.summary$`Untreated PyL-avid lesions` > 0
, 1, 0)
table(imaging.summary$untreated.lesion)

## generate 2x2 table
table.prog.untreated = table(untreated.lesion = factor(imaging.summary$untreated.lesion, level
s = c(0, 1), labels = c("No untreated","Any untreated")), prog = factor(imaging.summary$`Any p
rogression at 180 days?`, levels = c(0, 1), labels = c("Not progressed", "Progressed")))

## compute 95% CI
agresti.ci(1,19) ## no untreated
agresti.ci(6,16) ## any untreated

## fisher.test
fisher.test(table.prog.untreated)
```

5. New metastatic lesions at 6 months, SABR arm only, no untreated lesions vs at least 1 untreated lesion

```
imaging.summary$new.lesion = ifelse(imaging.summary$`New PyL Lesions` > 0, 1, 0)
```

© 2020 Phillips et al. *JAMA Oncology.*

```r
## generate 2x2 table
table.untreated.new.lesion = table(untreated.lesion = factor(imaging.summary$untreated.lesion, levels = c(0, 1), labels = c("No untreated","Any untreated")), new.lesion = factor(imaging.summary$new.lesion, levels = c(0, 1), labels = c("No new lesions", "Any new lesions")))

## compute 95% CI
agresti.ci(3,19) ## no untreated
agresti.ci(10,16) ## any untreated lesion

## fisher.test
fisher.test(table.untreated.new.lesion)
```

6. Parameters for Differential Abundance Determination using Cloud-based Adaptive Biosciences ImmunoSEQ Platform

```
[union]

# for anything related to 2 rxn ffpe samples, use CDR3 AA seq 'aminoAcid'

# nucleotide or aminoAcid

count = nucleotide

# threshold for whether or not a pvalue will be calculated (usually 5-10)

minTotal = 5

productiveOnly = True

# True if three samples are provided/trivariate analysis is desired
```

© 2020 Phillips et al. *JAMA Oncology.*

```
trivariate = False

[abundance] # added 4/5/17
# repertoire or nucleated
source = repertoire

[p value]
# fisher, binomial, or betabinomial
method = betabinomial
# two-sided, greater (sample 2 > sample 1), less (sample 1 > sample 2)
alternative = two-sided

[significance]
# bh, bonferroni, by, none
correction = bh
# can use alpha = 0.05
alpha = .01

[output]
# verbose = True (Union table will contain all sequences, instead of just significant ones)
```

© 2020 Phillips et al. *JAMA Oncology.*

verbose = trivariate: only trivariate plots are output

verbose = extra

reverse samples = no

pdf, svg, png are standard ones. Check OS for supported file types using matplotlib.pyplot.gcf().canvas.get_supported_filetypes(). readConfig function accepts ['eps', 'jpg', 'jpeg', 'pdf', 'png', 'ps', 'raw', 'svg', 'svgz'].

graphics format = pdf

[batch]

count the full repertoire in batch output (regardless of minTotal value)

count minTotal excluded sequences = True

[histogram]

log or linear

base = linear

labels = on

[scatter graph]

only applies to diffab plot, not trivariate

scale = frequency

© 2020 Phillips et al. *JAMA Oncology.*

scatter base = log

scatter verbose = extra

points = spwo

scatter point size = 10

axis label font size = 10

line of equality comparison = frequency

threshold for statistical comparison line = on

legend verbose = True

6. Plotting of Clonality and Differential Abundance using Rstudio

```
# load packages

library(tidyverse)


# create color palettes

cbPalette <- c("#0072B2", "#D55E00", "#009E73", "#56B4E9", "#E69F00", "#999999",
"#F0E442")

proPalette <- c("#009AA6", "#FECB00")


# read in table with clonal expansion and clonality values

values <- read.table('./values.tsv', sep = '\t', header = T)
```

© 2020 Phillips et al. *JAMA Oncology.*

```
# plot baseline clonality split by progression at 180 days

ggplot(data=values, aes(x=progression_180days, y=baseline_simpson_clonality,
fill=progression_180days)) +
  geom_boxplot(outlier.shape=NA) +
  geom_point(size = 3) +
  ylab('Baseline Simpson Clonality') +
  xlab('Day 180 Outcome') +
  theme_bw() +
  facet_grid(~Tx, scales = 'free', space = 'free') +
  scale_fill_manual(values=proPalette, name="Outcome") +
  theme(legend.position = "none",
        legend.background = element_rect(colour = "black"),
        legend.title = element_text(face = "italic")) +
  theme(axis.text.x = element_text(size=12,angle=0,hjust=.5,vjust=0,face="plain"),
        axis.text.y = element_text(colour="grey20",size=12,angle=0,hjust=1,vjust=0,face="plain"),
        axis.title.x = element_blank(),
        axis.title.y =
element_text(colour="grey20",size=15,angle=90,hjust=.5,vjust=.5,face="plain")) +
  theme(strip.text.x = element_text(size = 15))
```

© 2020 Phillips et al. *JAMA Oncology.*

plot the number of expanded and contracted clones for each subject, split by treatment group

```
ggplot() +
  geom_bar(data=values, aes(x=factor(subject_id), y=number_expanded), fill=cbPalette[2],
stat="identity") +
  geom_bar(data=values, aes(x=factor(subject_id), y=-number_contracted), fill=cbPalette[1],
stat="identity") +
  scale_y_continuous(limits=c(-max(values$number_contracted, values$number_expanded),
max(values$number_contracted, values$number_expanded))) +
  theme_bw() +
  facet_wrap(~Tx, scales = 'free_x') +
  ylab(expression(paste(symbol('\254'), " Contracted Clones | Expanded Clones ",
symbol('\256'), "  "))) + xlab("Subject") +
  theme(axis.text.x =
element_text(colour="grey20",size=10,angle=0,hjust=0.5,vjust=0,face="plain"),
        axis.text.y =
element_text(colour="grey20",size=10,angle=0,hjust=1,vjust=0.5,face="plain"),
        axis.title.x = element_text(colour="grey20",size=12,angle=0,hjust=.5,vjust=0,face="plain"),
        axis.title.y =
element_text(colour="grey20",size=12,angle=90,hjust=1.05,vjust=.5,face="plain")) +
  geom_hline(yintercept=0, linetype="dotted", colour="black")
```

© 2020 Phillips et al. *JAMA Oncology.*

eFigure 1. Total gross tumor volume in patients treated with SABR was not different in patients with or without progression at 180 days

Box and whisker plot depicting total gross tumor volume (cm^3) for patients treated with SABR, stratified by progression at 180 days. The solid box represents the interquartile range and whiskers represent 10th and 90th percentile values. P-value was calculated using Mann-Whitney test.

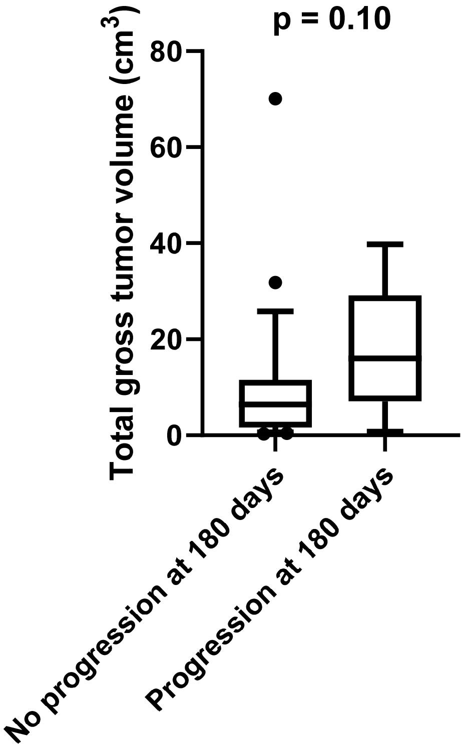

© 2020 Phillips et al. *JAMA Oncology.*

eFigure 2. Baseline circulating tumor DNA concentration is not associated with outcome

Box and Whisker plots depicting ctDNA allele fractions for patients on: **(A)** the SABR arm (n = 36), or **(B)** the observation arm (n = 18). Empty circles represent patients with undetectable ctDNA. The solid box represents interquartile range of values and whiskers represent 10th and 90th percentile values. P-values calculated using Mann-Whitney U Test.

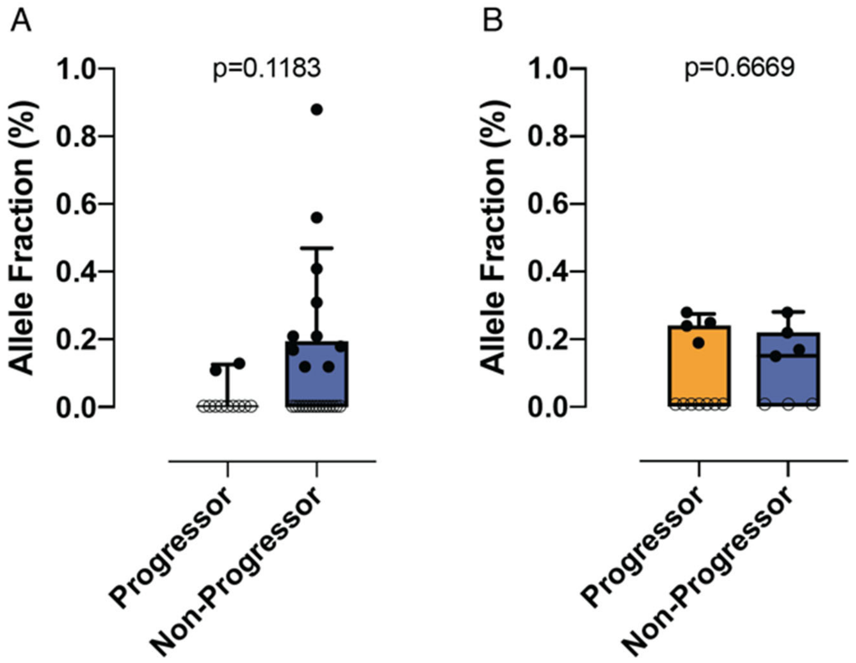

© 2020 Phillips et al. *JAMA Oncology.*

eTable 1. Treatment volume characteristics for SABR arm

Anatomical Site	Gross tumor volume (cm^3)	Radiation fractions	Total Dose (Gy)	BED3 (Gy)	Local Failure at 6 months
Obturator node	0.16	3	31.5	141.75	-
Obturator node	0.23	3	28.5	118.75	-
Rib	0.25	3	36	180.00	-
Common iliac node	0.29	5	36.25	123.85	-
Paraaortic node	0.32	3	27	108.00	-
Presacral node	0.43	5	35.25	123.85	-
Ischium	0.43	3	24	88.00	-
Rib	0.47	3	27	108.00	-
Rib	0.47	3	27	108.00	-
Acetabulum	0.58	3	30	130.00	-
Obturator node	0.61	5	40	146.67	-
Ischium	0.7	3	30	130.00	-
Inguinal node	0.72	5	40	146.67	-
Lumbar spine	0.76	3	21	70.00	-
Lumbar spine	0.78	4	48	240.00	-

© 2020 Phillips et al. *JAMA Oncology.*

Sacrum	0.9	3	33	154.00	-
Rib	0.93	3	27	108.00	-
Rib	0.93	3	27	108.00	-
Iliac node	1.22	3	33	154.00	-
Iliac node	1.28	5	45	180.00	-
Iliac node	1.42	4	44	205.33	-
Frontal bone	1.42	3	21	70.00	-
Obturator node	1.46	5	40	146.67	-
Retroperitoneal node	1.56	5	40	146.67	-
Inguinal node	1.58	5	35	116.67	-
Iliac node	1.65	5	45	180.00	-
Rib	1.68	3	30	130.00	-
Scapula	1.82	3	36	180.00	-
Rib	1.83	3	30	130.00	-
Rib	2.24	3	27	108.00	-
Iliac node	2.25	5	40	146.67	-
Pelvic node	2.27	3	30	130.00	-
Rib	2.28	3	30	130.00	-
Retroperitoneal node	2.46	5	45	180.00	-

© 2020 Phillips et al. *JAMA Oncology.*

Sternum	2.65	3	28.5	118.75	-
Iliac node	2.81	5	35	116.67	-
Supraclavicular node	2.99	5	37.5	131.25	-
Iliac bone	3.07	3	30	130.00	-
Iliac node	3.08	3	28.5	118.75	-
Iliac node	3.25	5	40	146.67	-
Retroperitoneal node	3.41	5	36.25	123.85	-
Mesorectal node	3.47	5	36.25	123.85	-
Presacral node	3.48	5	35	116.67	-
Iliac node	3.67	5	27.25	76.75	-
Iliac node	3.71	5	40	146.67	-
Iliac bone	4.09	3	33	154.00	-
Pelvic node	4.19	3	30	130.00	-
Retroperitoneal node	4.24	5	40	146.67	-
Iliac node	4.34	5	40	146.67	-
Iliac node	4.64	5	36.25	123.85	-
Iliac node	4.97	5	36.5	125.32	-

© 2020 Phillips et al. *JAMA Oncology.*

Subcarinal node	5	5	32	100.27	-
Common iliac/ retroperitoneal node	5.22	5	36.25	123.85	-
Iliac node	5.92	5	36.25	123.85	-
Retroperitoneal node	6.63	5	40	146.67	-
Presacral node	6.73	5	36.25	123.85	-
Retroperitoneal node	7.08	5	30	90.00	Yes
Iliac node	7.26	5	27.25	76.75	-
Sacrum/iliac bone	9.51	3	27	108.00	-
Iliac node	10.6	5	40	146.67	-
Paraaortic node	11	4	48	240.00	-
Rib	11.7	3	27	108.00	-
Retroperitoneal node	12	5	30	90.00	-
Iliac node	12.96	5	27.25	76.75	-

© 2020 Phillips et al. *JAMA Oncology*.

Rib	14.07	3	19.5	61.75	-
Sternum	15.63	3	28.5	118.75	-
Pubic symphasis	16.37	3	30	130.00	-
Paraaortic node	18.06	5	30	90.00	-
Acetabulum	24.53	3	24	88.00	-
Paraaortic node	26.1	5	35	116.67	-
Pubic bone	39.74	3	27	108.00	-
Retroperitoneal node	64.29	5	25	66.67	-

© 2020 Phillips et al. *JAMA Oncology*.

eTable 2. Distribution of lesions by treatment arm

	Observation (n = 18)	SABR (n = 36)	P-Value
**Lesions (mean)**			
Bone	0.44	0.64	
Nodal	1.22	1.39	
Total	1.66	2.03	
**Patients**			0.77
Node-only	12 (67%)	21 (58%)	
Bone-involved	6 (33%)	15 (42%)	

© 2020 Phillips et al. *JAMA Oncology.*

eTable 3. CTCAE v4.0 grade 1 adverse events

	New at 90 days		New at 180 days	
	Observation (n = 16)	SABR (n = 36)	Observation (n = 11)	SABR (n = 36)
Urinary Incontinence	2 (13%)	2 (6%)	1 (9%)	1 (3%)
Bruising	2 (13%)	0 (0%)	0 (0%)	1 (3%)
Fatigue	1 (6%)	5 (14%)	1 (9%)	1 (3%)
Anxiety	1 (6%)	2 (6%)	0 (0%)	0 (0%)
Urinary Retention	1 (6%)	1 (3%)	0 (0%)	1 (3%)
Localized Edema	1 (6%)	2 (6%)	0 (0%)	0 (0%)
Depression	1 (6%)	0 (0%)	0 (0%)	1 (3%)
Tremor	1 (6%)	0 (0%)	0 (0%)	0 (0%)
Pruritis	1 (6%)	0 (0%)	0 (0%)	0 (0%)
Proctitis	1 (6%)	0 (0%)	0 (0%)	0 (0%)
Anorexia	0 (0%)	0 (0%)	1 (9%)	0 (0%)
Cough	0 (0%)	3 (8%)	0 (0%)	1 (3%)
Diarrhea	0 (0%)	2 (6%)	0 (0%)	0 (0%)
Weight Loss	0 (0%)	2 (6%)	0 (0%)	1 (3%)
Constipation	0 (0%)	2 (6%)	0 (0%)	1 (3%)
Hemorrhoids	0 (0%)	1 (3%)	0 (0%)	0 (0%)
Neuralgia	0 (0%)	1 (3%)	0 (0%)	1 (3%)
Anemia	0 (0%)	1 (3%)	0 (0%)	0 (0%)
Bloating	0 (0%)	1 (3%)	0 (0%)	0 (0%)

© 2020 Phillips et al. *JAMA Oncology.*

Dizziness	0 (0%)	1 (3%)	0 (0%)	0 (0%)
Dehydration	0 (0%)	1 (3%)	0 (0%)	0 (0%)
Gastritis	0 (0%)	1 (3%)	0 (0%)	0 (0%)
Nausea	0 (0%)	1 (3%)	0 (0%)	0 (0%)
Weight Gain	0 (0%)	0 (0%)	0 (0%)	1 (3%)
Dry Mouth	0 (0%)	0 (0%)	0 (0%)	1 (3%)
Ascites	0 (0%)	0 (0%)	0 (0%)	1 (3%)
Esophageal Pain	0 (0%)	0 (0%)	0 (0%)	1 (3%)
Perineal Pain	0 (0%)	0 (0%)	0 (0%)	1 (3%)
Insomnia	0 (0%)	0 (0%)	0 (0%)	1 (3%)

© 2020 Phillips et al. *JAMA Oncology.*

eTable 4. CTCAE v4.0 grade 2 adverse events

	New at 90 days		New at 180 days	
	Observation (n = 16)	SABR (n = 36)	Observation (n = 11)	SABR (n = 36)
Urinary Incontinence	0 (0%)	1 (3%)	0 (0%)	1(3%)
Esophagitis	0 (0%)	1(3%)	0 (0%)	0 (0%)
Dizziness	0 (0%)	1(3%)	0 (0%)	0 (0%)
Bladder Infection	0 (0%)	0 (0%)	0 (0%)	1(3%)

© 2020 Phillips et al. *JAMA Oncology.*

eTable 5. Genes fully or partially covered in prostate cancer focused CAPP-Seq selector

A1CF	**BRCA2**	DDI1	GPR33	LIMCH1	ODF1	**PTEN**	STOML3
AADACL4	BSN	DDN	GRIA1	LMCD1	OSGIN2	PXDNL	TDRKH
ABCA13	C10orf11	DLG5	GRIN2B	LPPR4	OTOGL	RARS2	TIE1
ACPT	C14orf23	DNAH10	GUCY1A2	LRP1B	OTUD6B	**RB1**	TIGD3
ACTBL2	C1orf100	DNAH17	HEATR5A	LRRC69	PABPC1	RET	TMEM132D
ADHFE1	CACNA1E	DPP6	HFM1	LRRC8B	PAG1	RIMKLA	TMPRSS2
AGMO	CADM1	DSP	HHAT	LRRTM1	PAX9	RNF213	**TP53**
AIM2	CALB1	DUSP13	HMCN1	MAGEB6	PCDH15	RNF32	TPR
AKAP6	CASKIN1	EAPP	HOXC4	MAP3K1	PCDHA4	RP1	TRAF3IP2
**AKT1**	CCDC110	ECM1	**HRAS**	MATN2	PCDHA5	RTKN2	TRIM9
AL359195.1	CCL11	EP300	HRNR	MBIP	PCDHA9	RYR2	TRPA1
ALDH1A3	CCND1	EPHA10	HTR1E	MDGA2	PCDHB16	S100A7L2	TSSK6
ALPP	CDC42BPB	ERG	HTRA4	MDN1	PCDHB9	SALL1	UBTF
AMER3	CDK12	ETV1	**IDH1**	**MED12**	PCDHGA1	SALL3	UQCRB
AMZ1	**CDKN1B**	ETV4	IDH2	MIER3	PCDHGB1	SCN7A	USH2A
ANK1	**CDKN2A**	EXO1	IL31RA	MLL2	**PIK3CA**	SDCCAG8	USP28
ANK3	CDKN2B	EYA1	INHA	MLL3	**PIK3CB**	SEL1L3	VNN2
ANKFN1	CECR2	EYS	INTS8	MPEG1	PIK3CG	SEMA7A	VPS13B
ANKH	CENPE	FABP4	IRF4	MSC	**PIK3R1**	SETD9	VPS13C
ANKRD55	CFH	FAM196A	ISLR	MYC	PIKFYVE	SFTPD	WDR60
ANKRD6	**CHD1**	FAT3	JMJD1C	MYO15A	PLAT	SGK196	WDR64
**APC**	CHEK2	FBN1	KAT6A	MYOZ1	PLD5	SHANK1	WHSC1L1
**AR**	CHMP4C	FBN3	KAT6B	MYT1L	PLEKHA2	SIAH3	WSCD1
ARID2	CHRNA6	FCN1	KCNB2	NAMPT	PLEKHH2	SKIV2L2	XRCC2
ARID5B	CHRNB3	FGA	KCNN3	NAV2	PLK2	SLC10A5	ZBTB16
ASH1L	CLDN3	FGFR1	KCNT2	NDUFA13	PMP2	SLC20A2	ZCCHC14
ASIC2	CNOT3	**FOXA1**	KCNU1	NECAB1	POLE2	SLC26A7	ZEB1
ASTN1	COCH	FOXL1	KDM2B	NEUROD6	POLR3A	SLCO3A1	ZFAT
**ATM**	COL18A1	FREM2	KIF1A	NHLRC3	POM121L1	SLCO5A1	ZFHX3
ATP1B2	CPQ	FRMPD3	KLF12	NIN	POSTN	SORCS1	ZMYM3
AVPR1A	CREB3L4	FSCN3	KLF5	**NKX3-1**	POTEA	SOS2	ZNF142
BACH2	CSMD1	GABRG1	KLK3	NLRP4	POU5F1B	SPHKAP	ZNF292
BCHE	CSMD2	GBX1	KMT2C	NOM1	PREX2	SPINK1	ZNF704
BLACE	CTC-554D6.1	GJB7	KMT2D	NRXN3	PROSER1	**SPOP**	
BNC2	**CTNNB1**	GLI1	**KRAS**	NTM	PRPF8	SPTA1	
BORA	DACH1	GNAS	KRT25	NUDT13	PRUNE	SRFBP1	
**BRAF**	DCHS2	GPR124	KRT78	NXPE4	PTCHD2	SSTR4	
**BRCA1**	DCSTAMP	GPR158	LHFP	OBSCN	PTCHD3	STAB2	

*Putative driver genes in prostate cancer are listed in bold.

© 2020 Phillips et al. *JAMA Oncology.*

eTable 6. Mutations identified by CAPP-Seq in baseline plasma samples

Patient	Chromosome	Position[a]	Mutant allele	Ref. allele	Gene	Mutation type	Amino acid change[b]	Amino acid position[c]	Total depth[d]	Mutant AF (%)[e]
PRCA07	chr14	31346780	T	C	COCH	missense	PRO>SER	29/551	7770	0.21%
PRCA10	chr14	34985574	A	G	EAPP	missense	ALA>VAL	267/286	5150	0.14%
PRCA10	chr17	7578467	G	T	TP53	missense	THR>PRO	155/394	2780	0.43%
PRCA11	chr11	108142088	A	C	ATM	missense	THR>LYS	1011/3057	5319	0.13%
PRCA16	chr5	67575480	A	G	PIK3R1	missense	ALA>THR	185/725	5909	0.12%
PRCA20	chr9	21974792	A	G	CDKN2A	missense	SER>LEU	12/168	5322	0.19%
PRCA20	chr8	82665418	A	G	CHMP4C	missense	GLU>LYS	104/234	5525	0.14%
PRCA22	chr11	113934895	G	T	ZBTB16	missense	SER>ARG	291/674	7431	0.15%
PRCA25	chr7	150846019	C	A	GBX1	missense	VAL>GLY	250/364	4680	0.21%
PRCA26	chr8	128428278	G	A	POU5F1B	missense	GLU>GLY	56/360	2450	0.41%
PRCA29	chr8	2808683	C	A	CSMD1	missense	VAL>GLY	3385/3565	4541	0.15%
PRCA29	chr8	135577643	C	A	ZFAT	missense	VAL>GLY	970/1244	4470	0.18%
PRCA32	chr17	7573976	A	T	TP53	stop-gained	LYS>stop	351/394	4427	0.88%
PRCA33	chr10	76867759	G	T	DUSP13	missense	THR>PRO	120/189	4420	0.25%
PRCA34	chr5	98192348	A	T	CHD1	missense	LEU>PHE	1623/1711	4240	0.12%
PRCA36	chrX	66943627	A	C	AR	missense	GLN>LYS	903/921	3233	0.19%
PRCA42	chr8	42044954	C	G	PLAT	missense	ASP>GLU	167/563	3368	0.18%
PRCA43	chr5	14711343	C	A	ANKH	missense	VAL>GLY	481/493	3424	0.26%
PRCA43	chr8	3253730	G	A	CSMD1	missense	LEU>PRO	860/3565	4913	0.22%
PRCA45	chr14	51226692	C	T	NIN	missense	GLU>GLY	761/2134	6685	0.31%
PRCA46	chr17	7578518	G	C	TP53	missense	ALA>PRO	138/394	5638	0.09%
PRCA46	chr17	7578404	C	A	TP53	missense	CYS>GLY	176/394	4647	0.13%
PRCA47	chr11	108216635	A	G	ATM	missense-near-splice	VAL>ILE	2862/3057	4626	0.22%
PRCA48	chr12	129558602	G	A	TMEM132D	missense	SER>PRO	1040/1100	3872	0.28%
PRCA55	chr9	21974750	C	T	CDKN2A	missense	GLU>GLY	26/168	2879	0.56%

[a]Genomic coordinates are per hg19

[b]Predicted amino acid change resulting from mutation

[c]Position of predicted amino acid change / Length of full protein

[d]De-duplicated sequencing depth in plasma DNA at specified genomic position. De-duplication was performed as previously described by Newman et al, Nature Medicine, 2014 and Newman et al, Nature Biotechnology, 2016.

[e]Percentage of mutant reads in tumor or plasma (de-duplicated mutant reads / de-duplicated sequencing depth at that position)

© 2020 Phillips et al. *JAMA Oncology.*

eReferences

1. Chaudhuri AA, Chabon JJ, Lovejoy AF, et al. Early Detection of Molecular Residual Disease in Localized Lung Cancer by Circulating Tumor DNA Profiling. *Cancer Discov.* 2017;7(12):1394-1403.
2. Dash P, Fiore-Gartland AJ, Hertz T, et al. Quantifiable predictive features define epitope-specific T cell receptor repertoires. *Nature.* 2017;547(7661):89-93.

© 2020 Phillips et al. *JAMA Oncology.*
