# Supplementary material for: Outcomes of Observation vs Stereotactic Ablative Radiation for Oligometastatic Prostate Cancer: The ORIOLE Phase 2 Randomized Clinical Trial
Source: JAMA Oncol. 2020 Mar 26;6(5):650–9. doi: 10.1001/jamaoncol.2020.0147 (PMC7225913; doi:10.1001/jamaoncol.2020.0147)
Supplement: Supplement 3. — Data Sharing Statement [file jamaoncol-6-650-s003.pdf]

# Data Sharing Statement

Phillips. Outcomes of Observation vs Stereotactic Ablative Radiation for Oligometastatic Prostate Cancer. *JAMA Oncol*. Published March 26, 2020. 10.1001/jamaoncol.2020.0147

**Data**
**Data available:** Yes
**Data types:** Deidentified participant data
**How to access data:** tranp@jhmi.edu
**When available:** With publication

**Supporting Documents**
**Document types:** None

**Additional Information**
**Who can access the data:** Researchers whose proposed use of the data has been approved
**Types of analyses:** For approved future correlative analyses or for pooled or meta-analysis
**Mechanisms of data availability:** After approval of a proposal
